# Linear Vibronic Coupling Approach for Surface-Enhanced Raman Scattering: Quantifying the Charge-Transfer Enhancement Mechanism

**DOI:** 10.1021/acs.jctc.4c00061

**Published:** 2024-04-30

**Authors:** Francisco García-González, Juan Carlos Otero, Francisco J. Ávila Ferrer, Fabrizio Santoro, Daniel Aranda

**Affiliations:** † Andalucía Tech, Facultad de Ciencias, Departamento de Química Física, Universidad de Málaga, 29071 Málaga, Spain; ‡ Istituto di Chimica dei Composti Organometallici (ICCOM-CNR), Area della Ricerca del CNR, Via Moruzzi 1, I-56124 Pisa, Italy

## Abstract

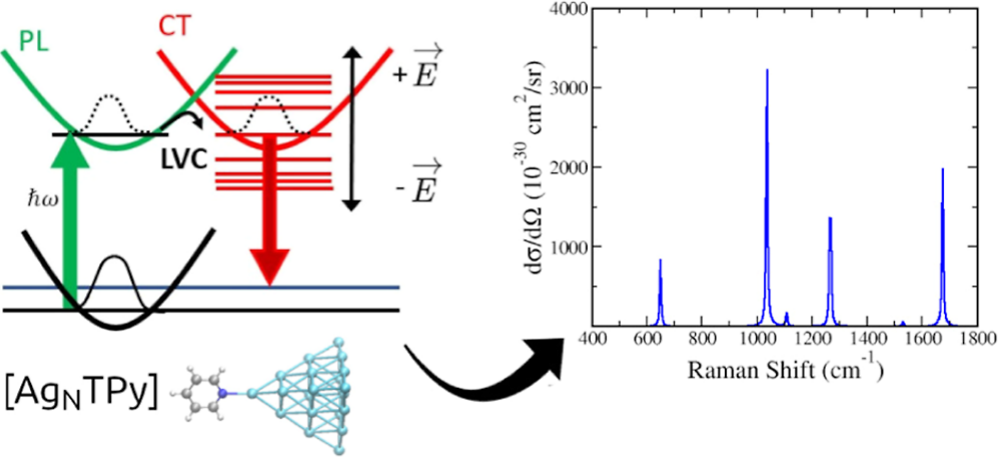

The outstanding amplification observed in surface-enhanced Raman scattering (SERS) is due to several enhancement mechanisms, and standing out among them are the plasmonic (PL) and charge-transfer (CT) mechanisms. The theoretical estimation of the enhancement factors of the CT mechanism is challenging because the excited-state coupling between bright plasmons and dark CT states must be properly introduced into the model to obtain reliable intensities. In this work, we aim at simulating electrochemical SERS spectra, considering models of pyridine on silver clusters subjected to an external electric field *E⃗* that represents the effect of an electrode potential *V*_el_. The method adopts quantum dynamical propagations of nuclear wavepackets on the coupled PL and CT states described with linear vibronic coupling models parametrized for each *E⃗* through a fragment-based maximum-overlap diabatization. By presenting results at different values of *E⃗*, we show that indeed there is a relation between the population transferred to the CT states and the total scattered intensity. The tuning and detuning processes of the CT states with the bright PLs as a function of the electric field are in good agreement with those observed in experiments. Finally, our estimations for the CT enhancement factors predict values in the order of 10^5^ to 10^6^, meaning that when the CT and PL states are both in resonance with the excitation wavelength, the CT and PL enhancements are comparable, and vibrational bands whose intensity is amplified by different mechanisms can be observed together, in agreement with what was measured by typical experiments on silver electrodes.

## 1 Introduction

Surface-enhanced Raman scattering (SERS) has been solidly established as a powerful technique for ultrasensitive analysis, thanks to the huge amplification of the Raman signal of molecules in the proximities of nanostructured surfaces of metal, inorganic, and even organic substrates.^1−3^ Several mechanisms contribute to this amplification, and standing out among them are the plasmonic (PL) and charge-transfer (CT) mechanisms.^4^ These two contributions refer to resonance Raman (RR) processes involving either the excitation of the strongly absorbing localized surface-plasmons of the metal nanostructure or excited states of the surface complex with metal/molecule charge-transfer characteristics.

The PL mechanism is nowadays recognized as the main contribution to the SERS enhancement, and numerous theoretical models have greatly improved the understanding of this fundamental phenomenon responsible for the Raman amplification and other related surface events, like surface-enhanced infrared absorption, surface-enhanced fluorescence, surface-enhanced Raman optical activity, or plasmon-enhanced two-photon absorption.^5,6^ Recent advances based on multimodel approaches and a combination of discrete interaction model/quantum mechanics have brought the simulations to nanoparticle sizes very close to realistic experimental conditions,^7−11^ overcoming a limitation that has hampered the understanding of SERS phenomenon for many years.

On the other hand, rationalization of the CT amplification remains more obscure because metal-to-molecule CT states are dark and therefore cannot be directly excited. As a matter of fact, it is very difficult to quantify the degree of contribution of the CT mechanism to the SERS amplification by experiments, since any change in the experimental conditions, nature of the substrate, solvent, etc., can greatly affect the CT excitation energies and, ultimately, its relative contribution to the overall enhancement.^12−14^

The most promising experimental approach to shed light on the CT mechanism is to carry out electrochemical SERS experiments (EC-SERS) in which the nanostructured substrate is used as an electrode.^15^ The applied electrode potential *V*_el_ confers direct control of the CT state energy, tuning or detuning it with respect to the plasmon resonance and the laser excitation.

By analyzing the changes in the relative Raman intensities for several *V*_el_’s, it is possible to detect the features of the resonance CT states, a mechanism that typically enhances specific bands. On the contrary, the PL mechanism is general, and for a given surface complex or adsorbate orientation to the surface, it equally would amplify all the modes of the spectrum aligned with the surface plasmon direction.^16^ Unfortunately, an experimental discernment of the PL and CT mechanism contributions is not possible without notably altering the experimental conditions. The aid of computational predictions can allow us to overcome these experimental limitations, and they have indeed proved to be an invaluable tool to obtain information on the surface complex and its electronic structure.^17,18^ The main evidence confirming the presence of CT processes in a particular SERS experimental spectrum is therefore given by qualitative individuation, at certain electrode potentials, of the bands of the molecule predicted by assuming a hypothetical resonance with a CT state.

However, theoretical simulations of EC-SERS systems require a suitable microscopic model that is able to take into account the main role of the electrode potential *V*_el_, a macroscopic parameter. Several strategies for modeling EC-SERS experiments have been reported in the literature. Such models introduce *V*_el_ in different ways (as effective charges of different metal clusters, external electric fields, combination of both, bimetallic clusters, semiempirical methods, or via electrolytes), and they allowed us to successfully confirm the participation of CT resonances in many SERS systems.^19−24^ Theoretical Raman intensities in resonance conditions are easily estimated by calculating the Franck–Condon (FC) factors (A-term in RR)^25^ for an electronic transition between the ground state of the surface complex and excited states with CT character.^13^ The calculated relative intensities have shown their ability to recognize and confirm the presence of CT processes in the SERS of benzene-like systems, accounting for the dependence observed on the applied potential,^16^ the nature of the molecule (pyridine, pyridazine),^16,26^ or on the symmetry of the surface complex (pyrimidine, 3-methylpyridine),^27,28^ for instance. Therefore, the calculated relative intensities of the bands can be considered as semiquantitative selection rules for SERS-CT processes.

Despite their success, these computational estimates hide the theoretical inconsistency. They are based on the A-term, a regime that is adequate to describe RR spectra of bright transitions. Therefore, their intensity is expected to be vanishingly small for dark or almost dark transitions like these CT states, which have a nearly zero transition dipole, due to the nearly zero differential overlap between the involved occupied and virtual orbitals. On the other hand, it is a matter of fact that many experimental SERS spectra are dominated by the strong enhancement of particular bands immensely related to such an hypothetical resonance with CT states. In order to solve this inconsistency, recent works incorporate the intensity-borrowing effects due to the Herzberg–Teller (HT) mechanism, described by the so-called Placzek’s B, C, and D terms. The inclusion of HT terms could successfully explain the intensification of several bands.^29−31^ Despite these results, invoking the perturbative HT approach does not appear to provide a fully robust framework for the estimation of enhancement factors due to the CT mechanism since Born–Oppenheimer (BO) approaches could break down when the CT state strongly couples with the plasmon, i.e., when the largest intensity borrowing is expected. Such a breakdown, which causes unreliable estimates of spectral intensities has been recently clearly shown in molecular systems for absorption and electronic circular dichroism spectroscopies,^32,33^ and also RR.^34^ Therefore, the development of suitable theoretical models that are able to properly describe the interaction between PL and CT excited states for different coupling regimes is needed to obtain a clearer picture of the CT enhancement mechanism and its relative contribution to the SERS amplification.

**Scheme 1:**
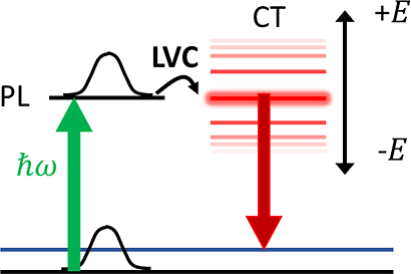
Schematic Summary of the Proposed Model The energy of a CT state is tuned with the applied electric field *E⃗*. When it becomes degenerate with a bright PL state in resonance with the excitation line, the features of the CT state can be observed in the SERS spectra due to excited-state coupling included in our model with a LVC Hamiltonian.

In this contribution, we focus on molecular models, which have been very often used in the literature,^19,20,24,35^ made up by an organic molecule, pyridine (Py) in this case, adsorbed on small metallic clusters. The novelty of the current contribution is that, in order to compute the EC-SERS spectra, we introduce a nonadiabatic approach inspired to those typically employed in the description of the spectroscopy and photophysics of molecules and molecular aggregates,^36−38^ aiming to reproduce resonance processes and intensity borrowing between PL and CT states (see Scheme 1). In practice, thanks to a suitable diabatization technique, we build up a generalized linear vibronic coupling (LVC) model which describes explicitly the bright states of the metal cluster (the “PL states”, PL) and the cluster/molecule CT states and how their respective energies and couplings are affected by the external electric fields representing *V*_el_. In this framework, the EC-SERS signals are retrieved by Fourier transforming correlation functions obtained by propagating nuclear wavepackets on the coupled PL and CT potential energy surfaces (PESs). The selective Raman enhancement of vibrational bands of the molecule related to metal-to-molecule CT resonant processes is explained by the very fast and extremely efficient population transfer from PL to CT states. The effectiveness of this nonadiabatic approach in a simpler intramolecular context has been recently proved for the RR spectra of several molecules exhibiting contributions from nearby dark states.^39,40^

On the other hand, being based on a variational approach, it can naturally describe both regimes of strong and weak couplings and also introduce an intensity-borrowing mechanism triggered by molecular vibrations. In this way, it is expected to solve possible issues encountered by adiabatic FC and HT vibronic approaches when applied to adiabatic states whose PL/CT mixing is variable and depends on the different tuning sensitivities of the respective energies caused by the applied bias. The approach presented here focuses on simulating the spectral features of CT states and obtaining rigorous estimates for the CT enhancement factors, given that the intensities obtained from this method are fully reliable. Therefore, the contribution of PL mechanism, which is the main source of intensity in most of the SERS experiments, is not explicitly addressed as several methods are well suited to this purpose,^7−11^ different to the CT mechanism.

## 2 Molecular Model

We selected as a molecular model a Py molecule attached to a triangular Ag_6_ cluster in order to mimic the microscopic structure of metallic adclusters located on the surface of rough macroscopic electrodes used in standard EC-SERS experiments. Ag_6_ has the advantage of conserving the Py C_2*v*_ point group if a proper orientation is selected, and this simplifies the vibrational analysis because normal modes do not mix between different symmetries. Of course, the small size of the cluster is very far from a true nanostructure. Therefore, to analyze the impact of the cluster dimension on our results, we also considered a larger Ag_20_ tetrahedron. This model has been previously adopted in the literature and provides a good balance between adclusters and cost for the necessary electronic structure computations.^21,35,41^

**Figure 1:**
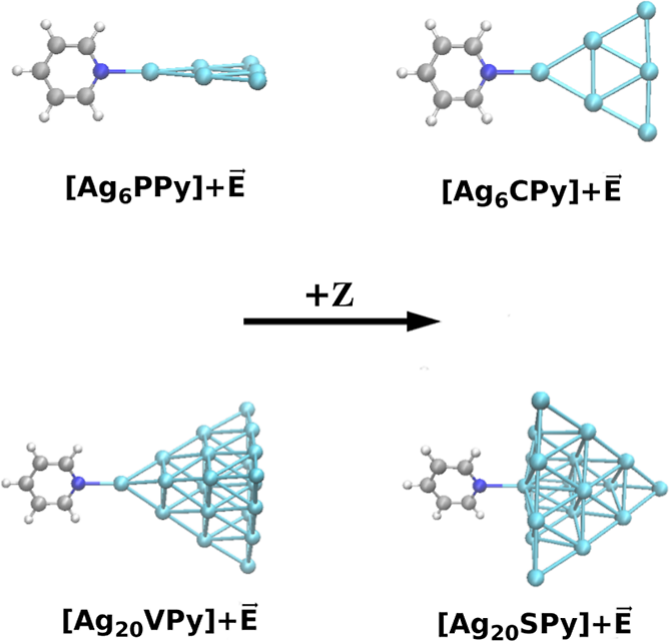
Molecular models for EC-SERS studied in this work and orientation of the *Z* Cartesian axis along with the applied electric field *E⃗*.

The nitrogen of Py is bonded to a single silver atom of the respective clusters, and we use the label [Ag_N_TPy]+*E⃗* to specify the system under investigation, where N denotes the number of atoms and the shape of the metallic moiety, *T* the conformation of the metal–molecule surface complex, and *E⃗* the external electric field along the *Z* Cartesian axis of module *E* (Figure 1); therefore, *E⃗* can be either *E*·*ẑ* or −*E*·*ẑ* when the field is applied parallel or antiparallel to the *Z* axis. This axis is aligned with the N–Ag bond in all cases, and *E⃗* mimics the effects of applied electrode potential *V*_el_. As it will be shown, *E⃗* mainly affects the energy of the CT states while barely modifying the energies of states of the metal cluster. According to the arrangement shown in Figure 1, an applied field +*E* shifts the CT energies to higher values, while −*E* decreases them. Therefore, calculated results with positive fields can be compared with the SERS spectra recorded at a more positive *V*_el_, whereas negative fields are related to the more negative values of *V*_el_. Triangular Ag_6_ (*N* = 6) and tetrahedral Ag_20_ (*N* = 20) have been studied as depicted in Figure 1. Both types of metal clusters have been previously used in the literature and have proved to be a reasonable approach to EC-SERS systems.^20,21^ For triangular complexes, we considered two cases: with Py and the silver cluster being perpendicular to each other (T=P) and with both moieties in the same plane (T=C). Also, two configurations for tetrahedral clusters have been studied, namely, with Py attached to a vertex (T=V) and to the center of a face (T=S); in both cases, Py is oriented so as to lay close to a symmetry plane of the tetrahedron.

## 3 Theory

The excited states of the supramolecular metal–molecule cluster relevant for the SERS phenomenon arise from the mixing of very strongly absorbing (PL states) and weaker local excitations (LE states) on the metal cluster with metal-to-molecule charge-transfer states (CT states). The role of these localized states can be investigated explicitly by performing a fragment-based diabatization of the molecule + cluster electronic states. In more detail, we built a fragment diabatization–linear vibronic coupling (FrD–LVC) model, analogous to the one proposed in ref (36) for excitonic systems. In the metal + molecule cluster, the mixing of PL, LE, and CT states is described by constant coupling terms, whereas linear couplings on normal coordinates introduce the effect of vibrational normal modes which are able to not only modulate the mixing among PL, LE, and CT but also describe the interaction between CT states of different nature. A further novelty in the present approach is that the FrD–LVC Hamiltonian also depends parametrically on the applied electrostatic field. This means that different values of both constant and linear terms are determined for each specific *E⃗*, tuning the energy position of the CT and therefore its mixing with PL states.

In practice, for each specific value of the applied potential *V*_el_, which is introduced in the calculations through the external electric field *E⃗*, we constructed a different FrD–LVC Hamiltonian **H** to describe the coupling between the diabatic localized states |*d*_*i*_⟩
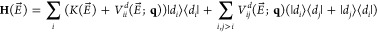
1where the parametric dependence on  is explicit. The kinetic energy term *K* depends on the diagonal matrix Ω, containing the vibrational frequencies of the ground electronic state |*g*⟩ associated with dimensionless normal coordinates **q** computed at each  and their conjugate momenta **p** (both **q** and **p** are column vectors)
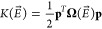
2

Notice that the normal coordinates (and therefore their associated momenta) are also recomputed for each *E⃗*, but in practice, from a qualitative point of view, the same Py normal modes are clearly distinguishable at each *E⃗*. Therefore, for the sake of simplicity, we avoid introducing a label to make such a dependence explicit.

PESs for each diabatic state *V*_ii_^d^ (;**q**) are described by the gradients along each coordinate η, , the frequencies , and the vertical excitation energies  at the equilibrium geometry of |*g*⟩, explicitly identified by the superscript “0”. On the other hand, interstate couplings *V*_*ij*_^*d*^(;**q**) are described as a linear function of **q** by means of the coefficients  and the constant coupling terms *E*_*ij*_^*0*^()
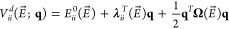
3
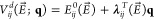
4where  and  are the (column) vectors of the coefficients  and , respectively. The diabatic states are defined taking as a reference the local excited states of the cluster and the CT states formed by single excitations from occupied orbitals of the cluster to virtual orbitals of the molecule. The parameters of the FrD–LVC Hamiltonian are determined by employing a maximum-overlap FrD.^36,42^ More details are given in the Supporting Information.

### 3.1 Computation of the RR Spectra in a Time-Dependent Framework

The intensity of a RR process from the ground vibrational state |*g*; 0⟩ to the final vibrational state |*g*; *f*⟩ depends on the corresponding transition polarizability tensor components.^25^ Their sum-overstate expression can be transformed into a time-dependent framework (see the Supporting Information for full derivation and refs (39 and 40) for details) obtaining the following expression
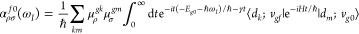
5where ρ and σ are Cartesian indices, ω_*I*_ is the incident radiation frequency, *k* and *m* are indices for all the relevant excited electronic states (i.e., those approximately resonant with the excitation frequency and all those coupled to them), *E*_*g*0_ is the energy of the initial state, and γ is the damping factor related to the lifetime of the electronic excited states, which in the following is considered identical for all states for simplicity. Notice that for the diabatic states of our basis set, ideally independent of the coordinates, we adopted the Condon approximation, which assumes that the Cartesian components of transition dipole moments are independent of the coordinates. This does not mean that the Condon approximation is invoked for the adiabatic states as well. Actually, as shown in the next section, their transition dipoles do change with the nuclear coordinates.
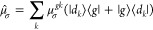
6

Due to the couplings between the electronic states, no analytical expression exists for the correlation functions in the integral of eq 5. However, they can be computed numerically by propagating in time |*d*_*m*_; *v*_*g*0_⟩, the vibrational ground state vertically photoexcited to the electronic state *m*, on the coupled PESs. Afterward, the polarizability components are obtained by a Fourier transform with Lorentzian damping.

RR intensities are obtained as the differential cross section σ′ for the incident frequency ω_*I*_ and the scattered frequency ω_*S*_.^25^ Different possible experimental settings exist, and in this specific case we consider one of the most commonly adopted, considering an incident light with perpendicular polarization and irradiance  and a scattered light of any polarization at an angle θ = π/2^25^
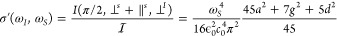
7where *c*_0_ and ϵ_0_ are the speed of light and the electric permittivity in vacuum, respectively, and *a*^2^, *g*^2^, and *d*^2^ are the so-called rotational invariants whose expressions are given in the Supporting Information.

### 3.2 Connection with Standard Adiabatic FC and HT Approaches

In order to establish a connection with usual adiabatic approaches to the computation of RR in FC or FC + HT (FCHT) approximation, it is useful to write down the expression of the transition polarizability in a time-independent sum-overstates approach.
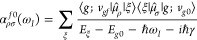
8where ξ are the vibronic eigenstates resulting from the coupling of the diabatic states described by the LVC Hamiltonian |ξ⟩ = *∑*_*k*,*n*_|*d*_*k*_; *v*_*kn*_⟩*C*_*kn*,ξ_. We notice that this expression is only formal because, in practice, it is not possible to obtain all vibronic eigenstates of the system we are investigating by diagonalization of the LVC Hamiltonian, and this is the reason we run the computation in the time domain with the expression in eq 5. If we now assume the validity of BO approximation (approximation *i*), we can get the vibronic eigenstates of the system by first diagonalizing the electronic Hamiltonian matrix, obtaining the adiabatic electronic states |*a*_*l*_(**q**)⟩ = *∑*_*k*,*l*_|*d*_*k*_⟩*D*_*k*,*l*_(**q**) and then computing their associated vibrational states |*v*_*l*,*n*_⟩. Notice that, for clarity, we explicitly indicate that the transformation matrix **D**(**q**) and therefore the adiabatic states |*a*(**q**)⟩ do depend on the nuclear coordinates. The BO transition polarizability becomes
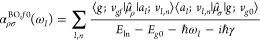
9

From eq 6, we can obtain the matrix elements of the transition dipole with the adiabatic states
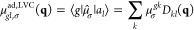
10

They are in general a nonlinear function of the normal coordinates since the coefficients *D*_*kl*_(**q**) are obtained by a diagonalization. If we expand such functions up to the linear terms (approximation ii), we obtain
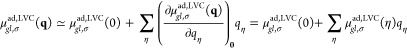
11

Finally, substitution into eq 9 leads us to an expression in which it is possible to recognize the usual A–D Albrecht’s terms^25^
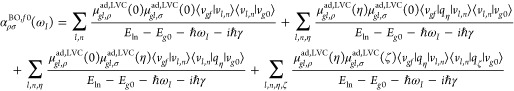
12

In conclusion, under the approximations i and ii, our method becomes equivalent to the standard adiabatic FC + HT approach,^25^ in which the derivatives of the transition dipole moment can be related to the coupling terms *h* defined by Lombardi and Birke.^1^ In a more general case, the method here presented is expected to be more accurate since it describes the possible existence of vibronic states with contributions from different electronic states, as usual in a strong regime or quasi-degeneracy regime, and implicitly it also accounts for beyond-HT nonlinearities in the dependence of the adiabatic transition dipoles on the nuclear coordinates. It is noteworthy that, adopting analytical sum rules, it has been shown in the literature that the total intensity of absorption and electronic circular dichroism spectra can be artificially and remarkably increased by the application of the FC + HT perturbative approach in cases of strong coupling.^32,33^ Similar artifacts should also be expected in RR even if they are more difficult to evidence with a simple analytical expression.

## 4 Computational Details and Methodology

All electronic structure calculations were performed with DFT and TD-DFT as implemented in Gaussian16^43^ using the functional CAM-B3LYP,^44^ due to its capability to predict reasonably good the energy of CT states, and the LANL2DZ basis set,^45−47^ for both Py and silver clusters, which utilizes an effective core potential for the metal atoms.

Our protocol consists of calculating optimized geometries for each [Ag_N_TPy]+ *E⃗* system, considering a sufficiently large range of *E⃗* to displace the CT excitation energies so as to tune and detune them with respect to the bright PL states. Herein, we adopted the FrD scheme described in ref (36) and implemented in the Overdia code to obtain the LVC Hamiltonian parameters.^48^ For the larger [Ag_20_Py] complex, in order to mitigate the computational cost, only the constant terms of the Hamiltonian were recomputed for all *E⃗*, whereas the parameters λ_*ii*_ and λ_*ij*_ were actually only computed for the case of maximum PL/CT mixing (*E⃗* = 10^–4^ a.u. and *E⃗* = 20 × 10^–4^ a.u., respectively, for S and V complexes), and the same values were used for the whole set of simulations. The normal modes localized on the metal moiety are not relevant in the spectral range of interest and were not included in the LVC model, except for some tests reported in the Supporting Information. Solvent effects were introduced with a polarizable continuum model^49^ in equilibrium solvation as implemented in Gaussian16 package only for Ag_6_ complexes, since solvation of the metal highly overestimates the effect of *E⃗* on the cluster in Ag_20_ complexes.

Nuclear wavepacket propagations on all bright PL states were performed for 150 fs, with 0.5 fs steps, using the multilayer extension of the multiconfigurational time-dependent Hartree (ML-MCTDH) method, as implemented in the Quantics code.^50−54^ A variable mean field with a fifth order Runge–Kutta and an integrator of 10^–7^ accuracy was employed during the propagations. See the Supporting Information for the ML-MCTDH trees. A typical γ value of 0.0544 eV ∼ 20 fs was used for the calculation of the RR spectra.^55^ This value was selected on the basis of our experience on other RR studies. Unfortunately, there is no experimental information providing reliable estimates of γ, especially for CT states in a roughened electrochemical surface. To gain insight of its impact on the excitation profiles, additional results are presented in the Supporting Information (Section S9.7) using a damping of 800 cm^–1^ (∼0.1 eV ∼ 15 fs).^35^

For the computation of adiabatic spectra, the vertical gradient (VG) model was used in combination with the FC or FCHT approaches for the adiabatic transition dipole moments.^56^ The dipole moment derivatives were computed as described in Section S1.4 by numerical differentiation in the proximity of the initial state structure, i.e., the ground state, which is known in the literature as FCHTi. Vibronic calculations of spectra were computed in the time-dependent formalism with the FCclasses3 code.^57−59^

### 4.1 Electric Field as an Analogue to *V*_el_

The comparison of computed SERS spectra with those measured experimentally is not straightforward, given that the applied electric potential in an electrochemical interface is a macroscopic parameter with no direct analogy at the atomic scale. Applied potentials modify the chemical composition and the electrical forces in electrode–electrolyte interfaces that modulate the properties of the metal–molecule hybrid system in the ground (strength of adsorption, charge redistribution between the metal and the adsorbate, etc.) and excited electronic states (mainly energies of the CT states). *V*_el_ controls the electrical charge separation in the double layer and, consequently, modulates the effective electric fields and chemical interactions affecting the metal–molecule system. Therefore, several strategies can be used to compare a set of EC-SERS spectra recorded at different potentials with calculated spectra for a series of theoretical models of the surface complex as reported in the literature. The approach followed in this work was to sample a set of *E⃗* and identify the case for which the mixing between PL and CT is maximum, as evidenced by the magnitude of the parameter *E*_ij_^0^ with respect to the energy gap between both states. This field can be related to the Py experimental spectrum at *V*_el_ = −0.6 V, in which the intensity of the aromatic CC stretching at 1600 cm^–1^ (mode 8a in Wilson’s nomenclature) is maximum, both in absolute and relative terms along all *V*_el_ values. The intensification of this mode has been related to the CT process in previous works.^19^ Then, the spectra for several nearby fields to this value were also computed to observe the tuning–detuning process and its impact on the intensities.

### 4.2 Diabatic States Definition

**Figure 2:**
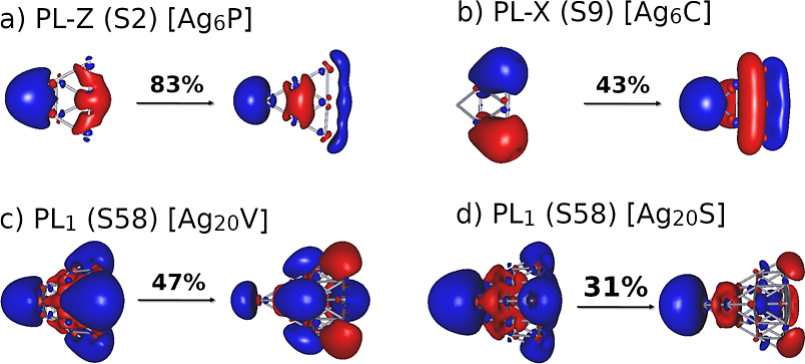
Main natural transition orbitals of relevant PL states for [Ag_6_P] (a), [Ag_6_C] (b), [Ag_20_V] (c), and [Ag_20_S] (d) clusters. Surface isovalues of 0.018 e/bohr^3^ for [Ag_6_P,C] and 0.016 e/bohr^3^ for [Ag_20_V,S] systems.

Table 1 shows the specific definition of the selected excited states: all local excitations on the metal cluster, both bright (PL) and weak/dark (LE), were taken to be identical to the TD-DFT states of the isolated cluster. The diabatic states were constructed from fragments with *E⃗* = 0, and the parameters of the LVC Hamiltonians were obtained by projecting them on a set of 20, 30, and 80 adiabatic excited states, respectively, for [Ag_6_PPy]+*E⃗*, [Ag_6_CPy]+*E⃗*, and [Ag_20_V,SPy]+*E⃗*. The LE states were included because they can indirectly couple the PL and CT states. Metal-to-molecule CT states were defined as a single transition from an occupied orbital from the metal cluster to a virtual orbital of Py. Several CT excitations from different occupied orbitals toward the lowest unoccupied molecular orbital (LUMO) and LUMO + 1 of Py were included for completeness. Molecule-to-metal CT states and local excitations on Py are too high in energy to interact with the PL states; therefore, they were not considered in the model. Specifically, the states included were 3 PL, 3 LE, and 3 CT for [Ag_6_PPy]; 1 PL and 1 CT for [Ag_6_CPy] (symmetry prevents most of PL and CT from interacting); and 3 PL and 5 CTs for both [Ag_20_VPy] and [Ag_20_SPy]. Figure 2 shows the NTOs for some PL states of each model.

**Table 1:** Definition of Diabatic States (*k*) for [Ag_6_PPy] Systems and Components (in atomic units) of the Electric Transition Dipole Moment with Respect to the Ground State (*g*), μ_σ_^*gk*^^a^

[Ag_6_PPy]+*E⃗*				
diabatic state (*k*)	definition	μ_*x*_^*gk*^	μ_*y*_^*gk*^	μ_*z*_^*gk*^
PL–X	S1 Ag_6_	5.51	0	0
PL–Z	S2 Ag_6_	0	0	5.66
LE–X1	S3 Ag_6_	0.90	0	0
LE–X2	S4 Ag_6_	2.19	0	0
LE–Z1	S5 Ag_6_	0	0	1.40
LE–Z2	S6 Ag_6_	0	0	0.73
LE–Y	S8 Ag_6_	0	0.07	0
PL–Y	S9 Ag_6_	0	–4.51	0
CT0–Z	HOMO Ag_6_ → LUMO Py	0	0	–0.08
CT1–Y	HOMO Ag_6_ → LUMO + 1 Py	0	0.01	0
CT0–X	HOMO–1 Ag_6_ → LUMO Py	0.03	0	0

a Symmetry imposes that μ is oriented along a single axis as specified in the table, while both CT and LE interact only with the PL state of a particular polarization as indicated by its label. The reference states read from Ag_6_ fragment were taken from the structure without applying any external field.

### 4.3 Normal Mode Selection

λ_*ii*_ and λ_*ij*_ parameters were computed for all normal modes of the metal–molecule systems; however, only modes with at least one |λ_*ii*_| or |λ_*ij*_| > 0.005 eV were taken into account for wavepacket propagations (see Figure S11 for the most relevant modes in Wilson’s nomenclature), leading to a total number of normal modes between 23 and 27 (full-dimensional Py). PL states show neither relevant gradients nor linear coupling parameters for Py modes, with the exception of mode 6a (elongation along the *C*_2_ axis). However, this is more likely an artifact of the model because of the proximity of Py to the metal cluster. In a true nanoparticle where the PL excitation is less localized among the atoms nearby the adsorption point, this effect would be mitigated. Because all displacements of Py normal modes on the PL and LE states were set to zero in the model, the RR intensity predicted for these modes arises uniquely from the coupling between PL and CT states. Therefore, this approach only focuses on the CT enhancement mechanism,^60^ thus disentangling it from the PL contribution, which is not considered in our model. More specifically, all nonvanishing *E*_*ij*_^0^ terms were included, while λ_*ii*_ and λ_*ij*_ parameters were only considered for CT states (λ_*ii*_) or between pairs of CT states (λ_*ij*_) because PL/CT and LE/CT linear terms were all predicted to be very small. Gradients and couplings along the normal modes of the metal moiety on PL and LE states were included in the model for only some tests as reported in the Supporting Information (Figures S29 and S30).

## 5 Results

Although we studied 4 systems, for the sake of brevity, the following discussion mainly focuses on one of them, [Ag_6_PPy]. The results obtained for [Ag_6_CPy], [Ag_20_VPy], and [Ag_20_SPy] complexes are reported in the Supporting Information, and they support the conclusions obtained for [Ag_6_PPy].

### 5.1 LVC Parameters

**Figure 3:**
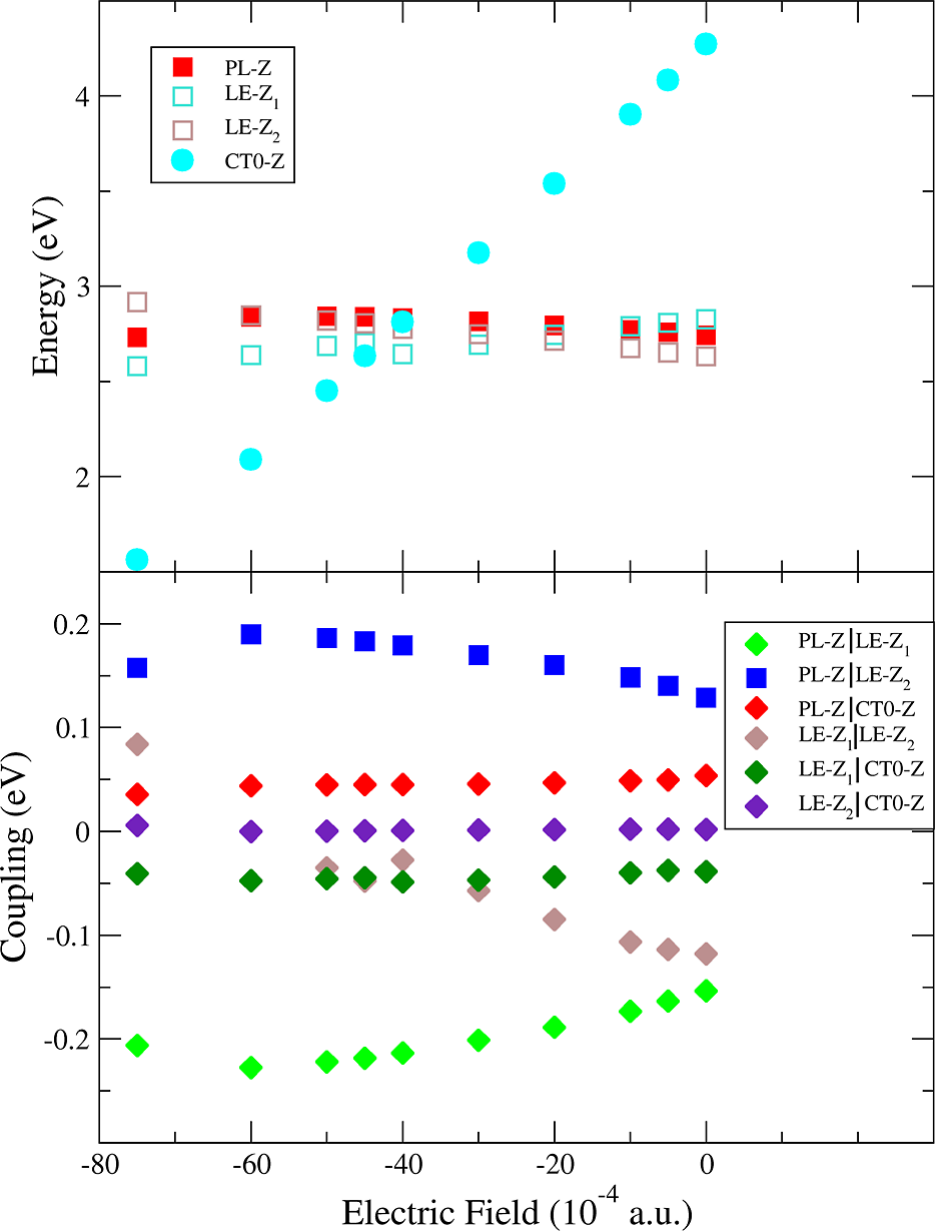
Dependence of excitation energies *E*_*ii*_^0^ for the diabatic states (top) and interstate coupling *E*_*ij*_^0^ (bottom) with *E⃗* for the *Z*-polarized set of complex [Ag_6_PPy].

The interactions among excited states are restricted by symmetry in [Ag_6_TPy] clusters. In the case of [Ag_6_PPy], the only nonzero couplings between diabatic states involve sets of states containing one PL, several LE, and one CT, all of them labeled by the axis along which the transition dipole moment of the PL is oriented (*X*, *Y*, and *Z*). The states belonging to different sets do not interact among them and, therefore, we can analyze separately each set parameters and discuss the population dynamics in three separated computations for photoexcitation to *X*-, *Y*-, and *Z*-polarized PL states. Nevertheless, for RR intensities, they all contribute to the polarizability invariants and must be considered together. Figure 3 shows the dependence of the diabatic energies *E*_*ii*_^0^ and zeroth-order coupling *E*_*ij*_^0^ on *E⃗* for the *Z*-polarized set. As expected, the energy of the metal PL and LE states is mostly insensitive to *E⃗*, while the CT ones show a linear dependence. Analogous behavior is observed for other excited states and systems (see Figure S21).

Concerning the *E*_*ij*_^0^ parameters, the coupling between PL/CT and LE/CT states basically remains constant with *E⃗*, and the *E*_*ij*_^0^ values range ±0.1 eV in most of the cases (see Figures 3 and S22). According to these results, the largest nonadiabatic effects are expected in [Ag_6_PPy]-40 and [Ag_6_PPy]-45, for which the bright PL, LE, and CT states are all almost degenerate. Therefore, the [Ag_6_PPy] complex and *E⃗* values in the range *E⃗* = −30 to −60 (10^–4^ a.u.) will be considered for the subsequent analysis of RR intensities during the tuning/detuning process between PL and CT.

For other complexes, [Ag_6_CPy], [Ag_20_VPy], and [Ag_20_SPy], the behavior is very similar, although the dependence on *E⃗* slightly changes, and the particular value of *E⃗* corresponding to the crossing point between PL and CT states is specific for each complex and orientation. These systems with less symmetry feature several interacting CT states, and their CT–CT *E*_*ij*_^0^ couplings are also nearly independent of *E⃗*. The CT0–CT1 coupling is always dominated by linear terms λ_*ij*_ through *b*_2_ symmetry modes.

Finally, because the PL and LE states are close in energy and have a sizable coupling, strong nonadiabatic effects can be expected between them. In fact, the LE/CT coupling is also large, and an alternative channel between PL and CT through LE states cannot be discarded. This is evaluated in the next section.

### 5.2 Population Dynamics of the Diabatic States and Raman Excitation Profiles

**Figure 4:**
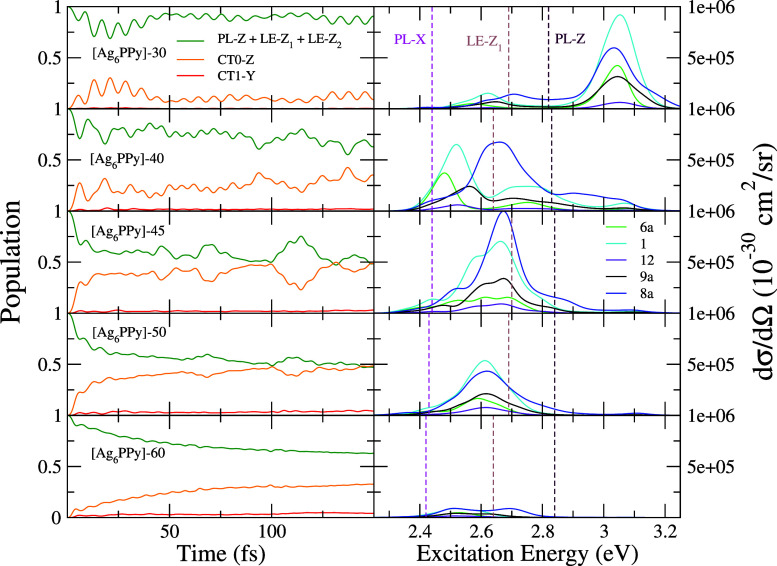
Left: population dynamics of diabatic states for the *Z* set of [Ag_6_PPy]+*E⃗* (*E* in 10^–4^ a.u.) after photoexcitation to PL-*Z*. All the states localized in the Ag_6_ fragment, both PL and LE, were added together for simpler visualization, see the Supporting Information for the individual populations. Right: RR excitation profiles for the main *a*_1_ modes of [Ag_6_PPy]+*E⃗*. The vertical lines represent the diabatic excitation energies *E*_*ii*_^0^ of the PL-*X* (magenta), LE-*Z*_1_ (brown), and PL-*Z* (maroon) excited states.

The time evolution of the diabatic state populations can provide an insight on the effectiveness of PL/CT coupling. Figure 4 (left) shows the population dynamics of the diabatic states after vertical excitation of the initial wavepacket to the PL-*Z* state for several [Ag_6_PPy]+*E⃗* systems (see Figures S26 and S27 for photoexcitation to PL-*X* and PL-*Y*). It is clearly observed that, as expected, the population transfer from the metal states to CT0 progressively increases (note that we sum together different metal states; see Figure S25 for individual populations) and shows a steeper initial slope when the energy difference between the CT0 and PL states Δ*E*_*CT*0|PL_ = *E*_*CT*0_ – *E*_PL_ becomes smaller and within the range of a vibrational quantum (ca. 0.20 eV for aromatic CC stretching). Due to the selected value of γ ∼ 20 fs, only the ultrafast regime of population dynamics contributes to the spectrum. Considering the CT populations at 50 fs, about 2.5 γ for different systems, they range from ∼8% in [Ag_6_PPy]-30 (Δ*E*_*CT*0|PL_ = 0.36 eV) to ∼26, ∼38, and ∼41% in [Ag_6_PPy]-40, [Ag_6_PPy]-45, and [Ag_6_PPy]-50 with Δ*E*_*CT*0|PL_ = −0.02, −0.20, and −0.39 eV, respectively. Once the CT is notably below PL, the population transfer is reduced as observed for [Ag_6_PPy]-60 (Δ*E*_*CT*0|PL_ = −0.75 eV and ∼24% population), evidencing the effect of the detuning. A similar pattern is observed for the *X* set and other complexes (Figures S26–S28 and S31–S36), which also reproduces the tuning/detuning process as *E⃗* becomes more negative and attains the largest population transfer to CT0 in [Ag_6_PPy]-45 (up to 43% for the *X*-set).

To investigate the role of the LE states, as shown in Figure S30, we performed a test by turning off the PL/CT direct couplings so that the CT is only accessible thanks to the couplings through LE states. As a result, for photoexcitation to PL-*X*, the population transfer to the CT states is halved, suggesting both channels are equally important. However, when photoexciting PL-*Z* (Figure S30), the population transfer is even larger than that when the PL/CT channel is active, pointing that the PL–LE–CT channel can be more efficient than the direct PL/CT transfer and that destructive interference between different channels may play a role. In any case, these results suggest that the dark LE states of the metal can be relevant in populating the CT states. It should be noted that such effect is expected to be attenuated in true nanostructured systems on which both bright PL and weakly absorbing LE states form degenerate bands, rather than discrete states as those found in small complexes like those studied here.

Interestingly, a very small population transfer to the CT1 state is observed in all cases. The only possible mechanism that can explain this finding in our model is the CT1/CT0 vibronic coupling through nontotally symmetric modes, mainly vibration 8b. In fact, the *E*_*ij*_^0^ coupling terms between PL-*Z* and CT1 are vanishing. However, the population transfer is too small to impact the SERS spectra as will be shown in the next section. On the other hand, CT1 state of the larger Ag_20_ cluster can be notably populated (until 10–15% on [Ag_20_SPy]-10 and [Ag_20_SPy]-20 complexes) due to the reduced symmetry and the stronger mixing of CT states with PL excitations as shown in Figures S34–S36.

Figure 4 (right) shows the SERS excitation profiles of the main totally symmetric modes actually observed in the experiments. Notably, the total Raman intensity (i.e., the area of the excitation profile) in the range between the resonance with the bright PL-*X* and PL-*Z* states (ca. 2.41–2.83 eV) correlates well with the population transfer to the CT states from all possible PL states: the maximum intensity in terms of differential cross section for a single mode is achieved for [Ag_6_PPy]-45 (10^–24^ cm^2^/sr) whose CT states receive most of the population from PL-*Z* and PL-*X* photoexcitations, while it is halved in [Ag_6_PPy]-40 and [Ag_6_PPy]-50 systems where population transfer is smaller, especially photoexciting up to PL-*X*, being greatly reduced in the [Ag_6_PPy]-30 and [Ag_6_PPy]-60 detuned cases.

It is also worth mentioning that the maximum intensity is mainly located in the energy region where the dark LE states lay instead of the bright PL states. This is due to the very strong coupling and mixing of both kinds of states, resulting in a large intensity transfer from the energy region of the PL states (ca. 2.42–2.83 eV) to the LE states (∼2.6 and ∼3.0 eV). However, this is a limitation of our model because the Ag_6_ cluster is small and the excited states are discrete instead of continuum bands like it is expected for larger nanoparticles with hundreds to thousands of atoms for a metal electrode. These cases would contain close-lying bright PL and dark states of the metal; therefore, the intensity transfer between nearly degenerate states has no practical effects on the spectrum. In principle, this intensity transfer between discrete states has to be taken into account when analyzing the spectra at specific ℏω_I_ energies on small clusters because the energies of PL and LE states are not totally insensitive to *E⃗* but shows a rather small dependence. However, in practice, it is shown in Figure S49 that moving slightly along the excitation profile when the CT is tuned, that is, *E⃗* from −40 to −50 (10^–4^ a.u.), does not remarkably affect the relative intensities and, in absolute terms, the intensities are in the same order of magnitude. Therefore, our results can be considered robust with respect to the selection of the excitation line.

Before concluding this section, it is worth revising our results in terms of the SERS selection rules. Figures S41–S45 show the transition polarizability components for the same five normal modes shown in Figure 4. Interestingly, the only nonvanishing components are mainly α_*zz*_ and to a minor extent α_*xx*_. In fact, for the energy range selected to analyze the spectra discussed in the next section (2.6–3.0 eV), the intensity is due almost exclusively to the α_*zz*_ component, which in our orientation is aligned perpendicular to the surface, in total agreement with the SERS selection rules.^1^

### 5.3 EC-SERS Spectra

#### 5.3.1 Discussion of Experimental Spectra and the Enhancement Mechanism of Py Bands

**Figure 5:**
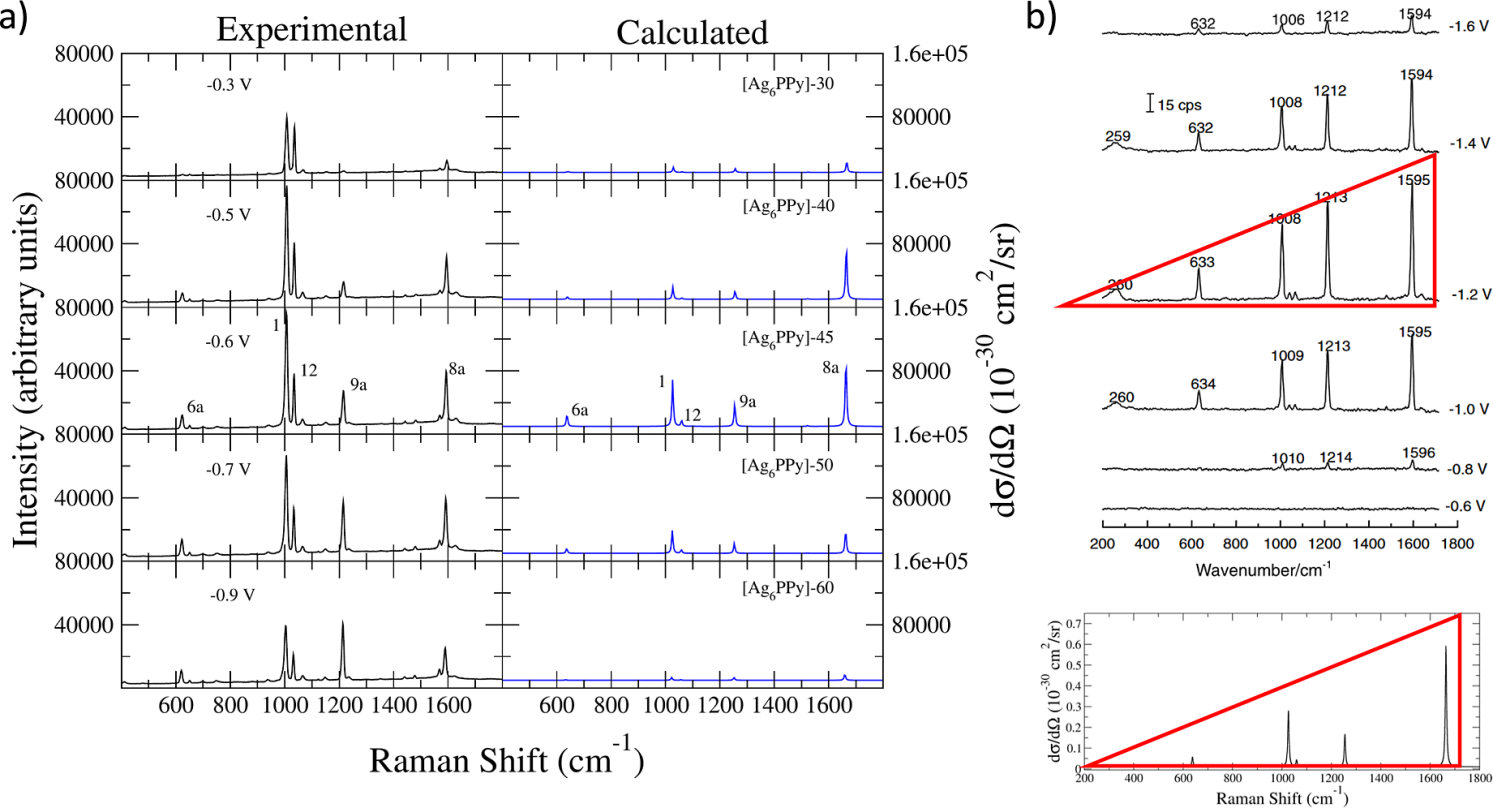
(a) Comparison of pyridine experimental EC-SERS spectra on a silver electrode from ref (61) using a 514.5 nm excitation line with calculated spectra of selected [Ag_6_PPy]+*E⃗* at the excitation energy of the LE-*Z*_1_ state. (b) Top: experimental EC-SERS spectra recorded on a nickel electrode where the triangular-like shape of the CT spectrum is highlighted. Adapted with permission from ref (63). Copyright 2004 Elsevier. Bottom: SERS spectrum for a pure CT photoexcitation obtained using the gradients λ_*ii*_ of the CT0-*Z* state calculated for [Ag_6_PPy]-45. All stick transitions for the spectra from a) and b) (bottom)were convoluted with a Lorentzian of half-width at half-maximum of 3 cm^–1^.

The analysis of the SERS spectra is complex. Therefore, it is worth putting in context the experimental observations. Resonant CT processes are mainly detected in SERS by analyzing the selective enhancement of particular bands when the energy of the laser or the electrode potential in this case is changed. Raman spectra of pure liquid pyridine or the aqueous solution are dominated by the very strong intensities of modes 1 and 12 recorded at ca. 1000 cm^–1^.^13^ This normal Raman spectrum resembles the EC-SERS of Py recorded at positive electrode potentials, on which the CT contribution is expected to be negligible, as shown by the spectrum registered at −0.3 V on a silver electrode^61^ (Figure 5a, top left). The complete set of EC-SERS recorded between 0.0 and −1.3 V from ref 61 is reported in Figure S46 for convenience. This behavior is expected for a general PL mechanism, assuming that the effect of adsorption on the static polarizability of Py in its ground electronic state is almost negligible in comparison with the PL-SERS contribution, that is, the relative intensities of the Raman bands for the surface complex are supposed to be very similar to the dissolved species.^6^

CT resonant processes are identified in EC-SERS experiments of benzene-like molecules by specific and selective enhancement of the highly characteristic 1600 cm^–1^ aromatic CC stretching band (mode 8a), accompanied by a stronger intensity of the overall spectrum. This is just the case of the EC-SERS spectra of Py recorded at −0.6 V, where the band of mode 8a shows its maximum intensity, decaying at more negative potentials (Figure 5a). These experimental results document that the CT mechanism is tuned by the applied potential and can reach enhancement factors comparable to those of the PL-SERS contribution. In fact, the 8a band is as intense as mode 12 and reaches half that of mode 1 in this experimental spectrum.^13,26,27,29^ The CT-SERS spectrum of many benzene-like molecules is also characterized by a particular shape^62^ since the peaks of all vibrational modes are approximately aligned along a straight line (see Figure 5b), resembling a triangle if one considers the band at higher wavenumbers and the baseline as the sides. Such shape has been observed in experiments using a nickel electrode and with an excitation line far from the PL resonance.^63^ The PL-SERS enhancement using this metal and this region is much weaker than in the cases of gold or silver PL surfaces, providing spectra almost exclusively dominated by the CT contribution (Figure 5b), where vibrations 1 and 12 appear to be much less intense than those recorded in silver, clearly highlighting the differences between the two mechanisms. Specifically, from this analysis, it can be concluded that in the silver electrode bands 1 and 12 benefit from the PL mechanism, as highlighted by the region −0.0 to −0.3 V, whereas band 1 can also gain intensity from the CT mechanism as shown in pyridine EC-SERS spectra on a nickel electrode.

The relative intensities of these CT-SERS spectra recorded on nickel nicely match those computed at the FC level (A-term) for the metal-to-molecule CT transition (see ref (19) and Figure 5b). However, at this level of theory, nothing can be said on the absolute intensities, which actually, strictly speaking, should be zero since the electronic transition is dark. The results discussed in the previous section have explained how CT states can achieve significant populations through nonadiabatic processes and, therefore, the next objective is to investigate if the calculated intensities are able to reproduce the overall and relative enhancement of the CT-bands observed in EC-SERS.

#### 5.3.2 Comparison of Experimental and Calculated Results

Experimental EC-SERS of Py recorded in the range −0.3 to −0.9 V are compared with the calculated LVC-SERS spectra with *E⃗* ranging from −30 to −60 (10^–4^ a.u.) (Figure 5a), using as incident photon energy for the calculated spectra the excitation energy of state LE-*Z*_1_ of each complex, which is nearby the excitation profile maximum as previously discussed. The intensity axes for all computed spectra are identical and adjusted so that the intensity of mode 8a in the [Ag_6_PPy]-45 complex has equal height to that in the experimental spectra recorded at −0.6 V.

The calculated CT-SERS spectra are characterized mainly by the strong intensity of mode 8a, with some contribution also from modes 1 and 9a, although in our model, the intensity of the latter band is underestimated with respect to the triangular shape previously discussed. The detuning process for more positive potentials (fields) is nicely reproduced by the calculations, and the behavior of the intensity of mode 8a agrees with the experimental trend. In addition, the intensity of modes 1 and 12 in experiments at these potentials mainly arises from the PL-SERS mechanism. This is documented by the fact that spectra recorded on the nickel electrode far from PL resonances, i.e., when only CT-SERS mechanism is active, shows a triangle-like shape and small intensities for modes 1 and 12.^63^ Moving to more negative potentials and fields, the detuning is also observed in our results, although the correspondence with experiment is less evident than for more positive *V*_el_. This can be due to the resonance with different PL states not considered here and occurring at strongly negatively charged adsorption sites or to the contribution of CT states of different nature (Rydberg). Such possibilities have been proposed in the literature to explain the intensification of mode 9a.^21,62^ Resonance with either PL states of charged adsorption sites or Rydberg CT states is also associated to a small reinforcement of mode 8a.^21,62^ Because such states are not included in the neutral, i.e., not charged, simple models we considered here, it can be expected that the behavior at more negative potentials is not fully reproduced in our calculations.

Concerning absolute intensities, approaches based on the HT mechanism have not been fruitful to estimate enhancement factors because the total intensities are extremely sensitive to *E⃗* and provide enhancement factors in the range 1–10^2^, peaking up to 10^10^ for specific cases and therefore making them unreliable.^21^ None of these enhancement factors would allow the CT contribution to be comparable to the PL mechanism, as actually observed in the experiments. On the other hand, as shown in Figure S56, the calculated intensity of the normal Raman spectrum of isolated Py, expressed as the differential cross section, is in the order of 10^–31^ cm^2^/sr, in agreement with previous values reported in the literature.^8,21,41^ Because the intensities shown in Figure 5b are in the order of 10^–26^ to 10^–25^ cm^2^/sr, the enhancement factor is of about 10^5^ to 10^6^, slightly above common experimental estimates for the CT mechanism^6^ (10^1^ to 10^4^, although some studies^64^ extend it to 10^7^). The CT enhancement factor value obtained from our calculations is close to the typical order of PL-SERS enhancement factors amounting to 10^6^ and therefore it is able to explain why the CT contribution can compete with the PL enhancement as actually observed in experiments during the tuning process between −0.3 and −0.9 V. In addition, Figures S57 and S58 show excitation profiles and spectra computed with a γ of 0.1 eV, showing a very similar shape, although intensities are scaled by about 1/5. Therefore, the expected enhancement factors are on the order of 10^4^ to 10^5^, still comparable with the PL mechanism for a reasonable range of γ values.

**Figure 6:**
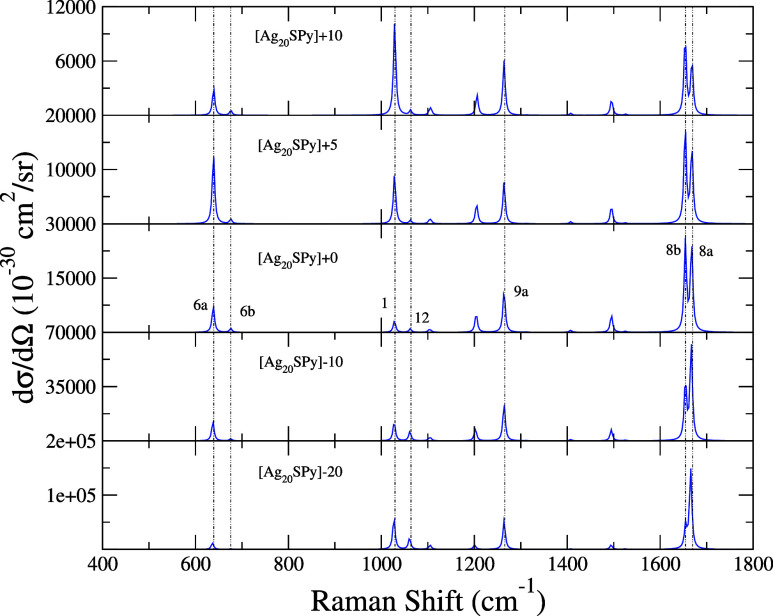
RR spectra for [Ag_20_SPy]+*E⃗* systems. The model includes 3 PL and 5 CT states. The value of ℏω_I_ was taken identical to the excitation energy of state PL_1_ of each system. All stick transitions were convoluted with a Lorentzian of half-width at half-maximum of 3 cm^–1^.

The results obtained for [Ag_6_CPy] are analogous to those of [Ag_6_PPy], as shown in Figure S48. The maximum intensity is found for the field value triggering the fastest and largest population transfer (*E* = 10^–4^ a.u.) with the enhancement factor reaching up to 10^6^. Similarly, the complexes with the Ag_20_ cluster also behave in this way. The largest population transfer and intensity are found for [Ag_20_VPy]+10 (see Figures S31 and S50) with an enhancement factor of 10^5^. Interestingly, these results also predict/document that the nontotally symmetric mode 8b can also attain very large intensities (see Figure 6 for [Ag_20_SPy]). Once again, the complex with the largest population transfer to the CT states ([Ag_20_SPy]-20) shows the largest intensity with an enhancement factor of 10^6^.

Finally, it is worth comparing these results with those based on the adiabatic FCHT approach for which the Albrecht terms are defined. Following the procedure described in Section 3, the derivatives of the transition dipole moment with respect to the normal coordinates are obtained. These derivatives are related to the vibronic coupling constants *h* defined by Lombardi and Birke^1^ and are responsible for the intensity arising from terms B, C, and D. At the adiabatic level (Tables S8–S11), the states of the metal cluster strongly mix, distributing the intensity between the PL and LE states. In addition, depending on the value of the electric field *E*, the CT is also mixed with the metal states, resulting in a strong coupling which affects the calculated intensities. In fact, the adiabatic states with the largest CT character acquire a non-negligible transition dipole moment due to the mixing with metal states. Figure S59 compares the excitation profiles computed with LVC and its adiabatic analogue VG including the HT expansion, i.e., FCHT|VG, for the [Ag_6_PPy]+*E⃗* set of complexes. Notably, when the mixing of CT and metal states is weak (*E* = −30 × 10^–4^ au), the adiabatic and LVC methods predict similar shapes. However, once the mixing is strong and the BO approach does not hold, the results are dramatically different. The same happens for the magnitudes of intensity: *E* = −40 and *E* = −45 (in 10^–4^ au), with the largest coupling showing profiles 10 to 1000 times larger for FCHT|VG than for LVC. Once the mixing decreases, both results are in a similar order of magnitude. Spectra taken at equivalent energies to LVC profiles (Figure S61) show very similar shape to the LVC ones in Figure 5; however, the intensities are much larger when *E* = −40 to −60 (in 10^-4^ a.u.), demonstrating once again that approaches based on the HT approach fulfill the SERS selection rules and qualitatively reproduce the spectral shapes but are not able to reproduce the results quantitatively.^21^ Therefore, the additional intensity with respect to LVC is actually an artifact, as reported in the literature for absorption and electronic circular dichroism spectroscopies.^32,33^ Finally, results based exclusively on the A term or FC|VG approach (Figures S60 and S62) reveal that this term contributes up to factors of 10^6^ due to the increase of the transition dipole moment of the mixed CT/metal state, but it rapidly decays as the CT state decouples of the metallic transitions, meaning that the B, C, and D terms are the relevant ones in the later situation. This is also in perfect agreement with what was described previously by Lombardi and Birke.^1^

## 6 Conclusions

In this work, we have presented a theoretical model to compute SERS intensities associated with the CT mechanism and reproduce the corresponding features of EC-SERS spectra. It considers metal–pyridine clusters under the influence of an external electric field and describes the couplings of the excited states through a nonadiabatic LVC Hamiltonian parametrized with a fragment-based diabatization approach. The extension of similar approaches developed to study the photophysics of molecules and to investigate the optical properties of adsorbates with metal nanostructures has not been reported up to now. Actually, while we were completing this article, a paper appeared in the literature which used a similar model for simulating absorption spectra of a molecule in a nanocavity.^65^ Still, the application of this approach to EC-SERS documenting its ability to interpret experimental spectra is a novelty of the present contribution. In particular, the model presented here allowed us to identify and dissect the role of PL/CT and CT/CT couplings at both the electronic and vibronic levels. The first coupling is independent of the vibrational motion and strong enough to give rise to the mixing of states when PL and CT become nearly degenerate, while the second coupling has mainly a vibronic origin, being activated through nontotally symmetric modes. The automatization of the proposed computational protocol is straightforward, and in fact it was repeated at different values of *E⃗*, providing a reliable dependence of intensities on *E⃗* and therefore allowing us to analyze the tuning and detuning processes of a specific CT state with a bright PL excitation.

Our results show a clear correspondence between the total Raman intensity estimated in the excitation profile with the transfer of population from PL to CT states in the ultrafast regime, which affects the spectra as determined by the selected value of γ. Specifically, the largest intensities are obtained for those systems showing the fastest population flow from bright PLs toward the dark CT states. The magnitude of such transfer is directly related to the magnitude of the nonadiabatic effects, estimated as the ratio of the electronic PL/CT coupling and the energy gap between both states. The computed spectra nicely reproduce the tuning and detuning processes and allow us to predict reliable enhancement factors for the CT mechanism in the order of 10^5^–10^6^, i.e., comparable to the PL enhancement factors. This finding nicely agree with the experimental observations, showing that the bands due to the CT compete in intensity to those amplified by the PL mechanism. Therefore, the CT-SERS mechanism can dominate the absolute and relative intensities of the spectra for some specific applied potentials *V*_el_. The four metal–pyridine systems studied here provide similar estimations, supporting the robustness of our predictions.

It is worth mentioning that although the procedure presented here accounts for excited-state coupling with a nonperturbative quantum dynamical approach with many differences with respect to the one described by Lombardi and Birke,^1,66^ based on the HT approximation, our approach predicts that vibrational modes showing the largest enhancement are of symmetries *a*_1_ and *b*_2_, in perfect agreement with the qualitative selection rules derived by them. In fact, our results point out that the totally symmetric modes become active because of the flow of population toward the CT states, whose geometry displacements along the totally symmetric modes result in the activation of *a*_1_ fundamentals. On the other hand, the reason for *b*_2_ mode enhancement is ascribed to the coupling between different CT states where the metal electron is placed either on the LUMO or the LUMO + 1 of Py. Additionally, the nonperturbative treatment of nonadiabatic effects allowed us to go beyond previous models and appropriately quantify the enhancement factors of the CT mechanism.

Finer details like the band structure of the CT are not perfectly reproduced by our model; in particular, the calculated relative intensity of mode 9a is smaller than what is observed in pure CT-SERS spectra recorded on a nickel electrode, i.e., it does not align with the maxima of other bands. Such subtle discrepancy in relative intensities is a common limitation to any model adopting an adcluster approach, where the metal is represented by small clusters with few atoms. On a more general ground, it should be highlighted that despite sharing the limitations intrinsic to the adcluster model with adiabatic models reported in the literature, our method overcomes the additional problems of these methods which make them not suitable to compute CT enhancement factors. In addition, the nice overall agreement between the calculated spectra for the different silver clusters employed here suggests that our results are robust. Noteworthily, to reproduce the full spectral shape recorded in the silver electrode, the PL contribution not introduced in our model should be accounted for. To this aim, it is necessary to simulate larger nanoparticles with hundreds to thousands of atoms; therefore, semiclassical or QM/MM approaches are better suited to estimate the enhancement factors due to this mechanism.^7,8,11^

SERS spectra are very complex to analyze and predict theoretically since they involve metal–molecule systems with very different macro/nanometric-molecular size scales. For this reason, the origin of the enormous enhancement of the Raman signal observed in SERS conditions has been the subject of controversy for the last 50 years. In this respect, EC-SERS experiments present a unique opportunity to easily control *V*_el_, which is one of the key parameters in electrified interfaces. *V*_el_ is able to tune the relative energy of the CT states of the surface complex and, therefore, modulate the coupling between states of different nature. Therefore, we foresee that the development of theoretical models able to describe EC-SERS experiments, like the one presented here, will pave the way for a better interpretation of SERS spectra and will allow one to improve the understanding of the electronic structure of small molecules adsorbed on surfaces, information only available to very few experimental techniques under mild conditions of pressure and temperature, or to computational methods. In the future, we will also test the possibility to extend the computational model presented here to investigate related processes like the energy and electron transfer between organic molecules and surfaces or in organometallic complexes. Some possible applications related to these processes are worth mentioning, like luminescent devices,^67^ phototherapy,^68,69^ heterogeneous catalysis,^70^ or transfer phenomena between surfaces and adsorbed molecules.^71^

## Supporting Information Available

Additional theoretical details, ML-MCTDH trees for all the studied systems, definition of main normal modes of SERS spectra, properties of most relevant excited states included in the diabatization, definition of diabatic states and their properties, natural transition orbitals of the main TD-DFT states, dependence of the LVC Hamiltonian parameters with *E⃗*, additional population dynamics results, and additional spectra and excitation profiles (PDF)
