# Supplementary material for: Linear Vibronic Coupling Approach for Surface-Enhanced Raman Scattering: Quantifying the Charge-Transfer Enhancement Mechanism
Source: J Chem Theory Comput. 2024 Apr 30;20(9):3850–63. doi: 10.1021/acs.jctc.4c00061 (PMC11099975; doi:10.1021/acs.jctc.4c00061)
Supplement: Supplementary file 1 — ct4c00061_si_001.pdf [file ct4c00061_si_001.pdf]

Supporting Information: A Linear Vibronic Coupling Approach for Surface-Enhanced Raman Scattering: Quantifying the Charge-Transfer Enhancement Mechanism

Francisco García-González,† Juan Carlos Otero,† Francisco J Ávila Ferrer,† Fabrizio Santoro,*,‡ and Daniel Aranda*,†

†*Andalucía Tech, Facultad de Ciencias, Departamento de Química Física, Universidad de Málaga, 29071-Málaga, Spain*

‡*Istituto di Chimica dei Composti Organometallici (ICCOM-CNR), Area della Ricerca del CNR, Via Moruzzi 1, I-56124 Pisa, Italy*

E-mail: fabrizio.santoro@pi.iccom.cnr.it; aranda@uma.es

Contents

1 Additional Theoretical Remarks **S-4**

1.1 Details on the Diabatization . . . S-4

1.2 Derivation of the Time-Dependent Expression of the Polarizability . . . S-4

1.3 Full Expression of Rotational Invariants . . . S-6

1.4 Details on the Computation of Energy Gradients and Transition Dipole Derivatives for Adiabatic States . . . S-6

2 Multi-Layer MCTDH Trees **S-8**

2.1 [Ag_6P,CPy]+$\vec{E}$ . . . S-8

2.2 [Ag_{20}V,SPy]+$\vec{E}$ . . . S-16

3 Normal Modes of Pyridine **S-18**

4 TD-DFT Excited States **S-19**

4.1 Excited States for [Ag_6P,CPy] Fragments . . . S-19

4.2 Excited States for [Ag_{20}V,SPy] Fragments . . . S-20

5 LVC Diabatic States **S-21**

5.1 Definitions for LVC Diabatic States . . . S-21

5.2 Transition Dipole Moments for LVC Diabatic States . . . S-22

6 Natural Transition Orbitals **S-23**

6.1 Natural Transition Orbitals for [Ag_6P,C] . . . S-23

6.2 Natural Transition Orbitals for [Ag_{20}V,S] . . . S-26

7 Dependence of the LVC Hamiltonian with the Electric Field **S-31**

7.1 PL, LE and CT Diagonal terms . . . S-31

7.2 PL/CT and LE/CT constant off-diagonal terms . . . S-32

7.3 PL/PL, PL/LE and LE/LE constant off-diagonal terms . . . S-33

7.4 CT-CT constant off-diagonal terms . . . S-34

8 Populations Dynamics **S-35**

8.1 [Ag_6P,CPy]+$\vec{E}$ Wavepacket Propagations . . . S-35

8.2 [Ag_{20}V,SPy]+$\vec{E}$ Wavepacket Propagations . . . S-42

9 Additional Spectra and Excitation Profiles **S-49**

9.1 Additional Excitation Profiles S-49

9.2 Transition Polarizability Components S-53

9.3 Experimental Spectra S-58

9.4 [Ag_6P,CPy]+$\vec{E}$ Spectra S-59

9.5 [Ag_{20}V,SPy]+$\vec{E}$ Spectra S-62

9.6 Py Normal Raman Spectrum S-68

9.7 Results with $\gamma = 0.1$ eV S-69

9.8 Comparison with adiabatic methods S-71

References **S-76**

1 Additional Theoretical Remarks

1.1 Details on the Diabatization

Performing a maximum-overlap fragment diabatization,[S1,S2] the diabatic states were described as a combination of the adiabatic states of each system described by the transformation $\mathbf{D}$, from which vertical energies $E^0_{ii}(\vec{E})$ and constant couplings $E^0_{ij}(\vec{E})$ for the LVC Hamiltonian can be retrieved, where the superscript 0 specifies that they are computed at the reference geometry, i.e., at the ground state minimum. To include the effect of vibrational modes q_η in the Hamiltonian, couplings $\lambda_{ij}(\vec{E};\eta)$ (and gradients $\lambda_{ii}(\vec{E};\eta)$) were calculated by displacing the systems along the normal coordinates by $\Delta_\eta = \pm 0.1$, calculating the adiabatic potential energies $\mathrm{V}^{\mathrm{ad}}(\vec{E};\pm\Delta_\eta)$, then transforming them to diabatic state potentials, and finally computing the couplings as numerical derivatives:

$$V^d(\vec{E};\Delta_\eta) \quad = \quad \mathbf{D}^{\mathrm{T}} V^{ad}(\vec{E};\Delta_\eta)\mathbf{D} \tag{1}$$

$$\lambda_{ij}(\vec{E};\eta) \quad = \quad \frac{V^d_{ij}(\vec{E};\Delta_\eta) - V^d_{ij}(\vec{E};-\Delta_\eta)}{2\Delta_\eta} \tag{2}$$

1.2 Derivation of the Time-Dependent Expression of the Polarizability

The polarizability tensor components can be expressed in a time-independent framework as follows:

$$\alpha^{f0}_{\rho\sigma}(\omega_I) \quad = \quad \sum_\xi \frac{\langle g; v_{gf}|\hat{\mu}_\rho|\xi\rangle \langle \xi|\hat{\mu}_\sigma|g; v_{g0}\rangle}{E_\xi - E_{g0} - \hbar\omega_I - i\hbar\gamma} \tag{3}$$

where ρ and σ are Cartesian indices, ω_I is the incident radiation frequency and the sum runs over all the possible intermediate vibronic eigenstates ξ. Notice that in principle these vibronic states are mixed and do not belong to any particular diabatic state. Therefore, E_{g0} and E_ξ are, respectively, the energy of the initial and intermediate states.

The parameter γ is the damping factor related with the lifetime of the electronic excited states, which in the following is considered independent of k. Exploiting the limit for small γ:

$$\lim_{\gamma\to 0^+} \frac{1}{E_\xi - E_{g0} - \hbar\omega_I - i\hbar\gamma} \quad = \quad \frac{1}{\hbar}\int_0^\infty dt e^{-it(E_\xi - E_{g0} - \hbar\omega_I - i\hbar\gamma)/\hbar} \tag{4}$$

we can obtain a time-dependent expression for the polarizability tensor:[S3]

$$\alpha^{f0}_{\rho\sigma}(\omega_I) = \frac{1}{\hbar}\int_0^\infty dt e^{-it(-E_{g0}-\hbar\omega_I-i\hbar\gamma)/\hbar}\sum_\xi \langle g; v_{gf}|\hat{\mu}_\rho e^{-iHt/\hbar}|\xi\rangle \langle\xi|\hat{\mu}_\sigma|g; v_{g0}\rangle \tag{5}$$

and taking into account the closure relation $\sum_\xi |\xi\rangle\langle\xi| = 1$ we get:

$$\alpha^{f0}_{\rho\sigma}(\omega_I) = \frac{1}{\hbar}\int_0^\infty dt e^{-it(-E_{g0}-\hbar\omega_I-i\hbar\gamma)/\hbar}\sum_k \langle g; v_{gf}|\hat{\mu}_\rho e^{-iHt/\hbar}\hat{\mu}_\sigma|g; v_{g0}\rangle \tag{6}$$

Since we use diabatic states, ideally independent of the coordinates, we can adopt the Condon approximation, assuming that the Cartesian components of transition dipole moments are independent of the coordinates.

$$\hat{\mu}_\sigma = \sum_k \mu^{gk}_\sigma(|d_k\rangle\langle g| + |g\rangle\langle d_k|) \tag{7}$$

So that Eq. 6 becomes

$$\alpha^{f0}_{\rho\sigma}(\omega_I) = \frac{1}{\hbar}\sum_{km}\mu^{gk}_\rho\mu^{gm}_\sigma\int_0^\infty dt e^{-it(-E_{g0}-\hbar\omega_I)/\hbar-\gamma t}\langle d_k; v_{gf}|e^{-iHt/\hbar}|d_m; v_{g0}\rangle \tag{8}$$

The correlation functions:

$$\varphi^{f0}_{km}(t) = \langle d_k; v_{kf}|e^{-iHt/\hbar}|d_m; v_{g0}\rangle \tag{9}$$

can be computed by propagating with QD methods the vibrational ground state photoexcited to electronic state m $|d_m; v_{g0}\rangle$ on the coupled potential energy surfaces. Then, it is then possible to obtain the total correlation function tensor:

$$\Phi^{f0}_{\rho\sigma}(t) = \sum_{k,m}\mu^{gk}_\rho\mu^{gm}_\sigma\varphi^{f0}_{km}(t) \tag{10}$$

whose Fourier transform gives $\alpha^{f0}_{\rho\sigma}$ at excitation frequency ω_I:

$$\alpha^{f0}_{\rho\sigma}(\omega_I) = \frac{i}{\hbar}\int_0^\infty dt e^{t(iE_{g0}/\hbar+iw_I-\gamma)}\Phi^{f0}_{\rho\sigma}(t) \tag{11}$$

1.3 Full Expression of Rotational Invariants

$$
\begin{aligned}
a^2 &= \left|\frac{\alpha_{xx}^{fi}+\alpha_{yy}^{fi}+\alpha_{zz}^{fi}}{3}\right|^2 && (12)\\
g^2 &= \frac{1}{2}\Big[|\alpha_{xx}^{fi}-\alpha_{yy}^{fi}|^2+|\alpha_{xx}^{fi}-\alpha_{zz}^{fi}|^2+|\alpha_{yy}^{fi}-\alpha_{zz}^{fi}|^2 \\
&\quad +\frac{3}{2}\Big(|\alpha_{xy}^{fi}+\alpha_{yx}^{fi}|^2+|\alpha_{xz}^{fi}+\alpha_{zx}^{fi}|^2+|\alpha_{yz}^{fi}+\alpha_{zy}^{fi}|^2\Big)\Big] && (13)\\
d^2 &= \frac{3}{2}\Big[|\alpha_{xy}^{fi}-\alpha_{yx}^{fi}|^2+|\alpha_{xz}^{fi}-\alpha_{zx}^{fi}|^2+|\alpha_{yz}^{fi}-\alpha_{zy}^{fi}|^2\Big] && (14)
\end{aligned}
$$

1.4 Details on the Computation of Energy Gradients and Transition Dipole Derivatives for Adiabatic States

As described in the main text, the electronic adiabatic states can be obtained by diagonalization of the LVC Hamiltonian with the transformation:

$$|\mathbf{a}^{(r,LVC)}(\mathbf{q})\rangle = |\mathbf{d}^{(r)}\rangle\,\mathbf{D}(\mathbf{q}) \tag{15}$$

where $\mathbf{D}(\mathbf{q})$ is the transformation matrix and $|\mathbf{a}^{(r,LVC)}(\mathbf{q})\rangle$ are the adiabatic states, where the r superscript explicitly indicates that it is a row vector. The energies of the states is obtained from the diagonal elements $\mathbf{E}^{ad,LVC}$

$$\mathbf{E}^{ad,LVC}(\mathbf{q}) = \mathbf{D}^T(\mathbf{q})\mathbf{V}^{dia}(\mathbf{q})\mathbf{D}(\mathbf{q}) \tag{16}$$

For small displacements, the adiabatic gradients at $\mathbf{q}=\mathbf{0}$ (Franck-Condon point, FC) can be obtained from the transformation above and the numerical differentiation:

$$\lambda_{ii}^{ad,LVC}(\eta) = \left(\frac{\partial V^{ad,LVC}(\mathbf{q})}{\partial q_\eta}\right)_{\mathbf{0}} \simeq \frac{V^{ad,LVC}(+\Delta_\eta)-V^{ad,LVC}(-\Delta_\eta)}{2\Delta_\eta} \tag{17}$$

Notice that because of the constant terms E_{ii}^0 the adiabatic and diabatic gradients are not identical.

The transition dipoles of the adiabatic states are calculated again from applying the transformation matrix in the FC structure to the diabatic transition dipole moments, which are considered independent of the coordinates and equal to the values they have at the FC position. Therefore, to obtain the values of the adiabatic transition dipole moments at the displaced geometries the rotation matrices $\mathbf{D}(\pm\Delta_\eta)$ must be used on each case at $\pm\Delta_\eta$ for each q_η. The adiabatic states in the displaced geometries and derivatives are calculated by

$$|\mathbf{a}^{(r,LVC)}(\pm\Delta_\eta)\rangle = |\mathbf{d}^{(r)}\rangle\,\mathbf{D}(\pm\Delta_\eta) \tag{18}$$

$$\left(\frac{\partial \boldsymbol{\mu}_{gl}^{ad,LVC}(\mathbf{q})}{\partial q_\eta}\right)_{\mathbf{0}} \simeq \frac{\boldsymbol{\mu}_{gl}^{ad,LVC}(+\Delta_\eta) - \boldsymbol{\mu}_{gl}^{ad,LVC}(-\Delta_\eta)}{2\Delta_\eta} \tag{19}$$

these data are all what needed to run FC|VG and FCHT|VG because both assume that the normal modes and frequencies of all modes are identical to those of the ground state.

2 Multi-Layer MCTDH Trees

2.1 [Ag_6P,CPy]+$\vec{E}$

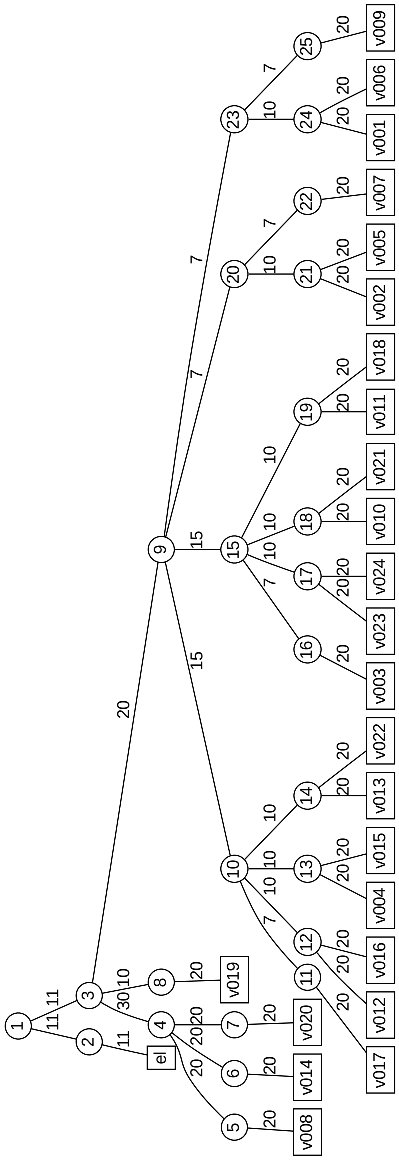

Figure S1: ML-MCTDH tree for the [Ag_6PPy]+0 system.

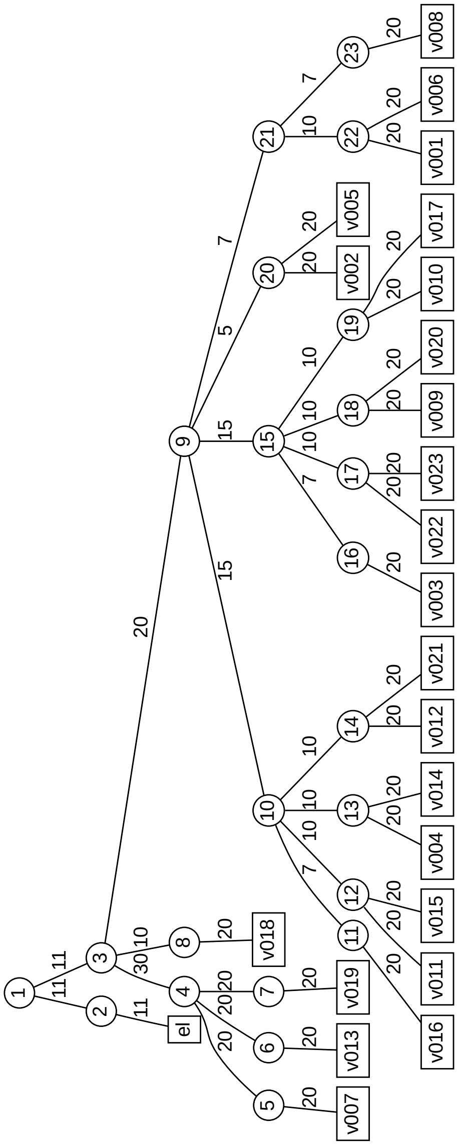

Figure S2: ML-MCTDH tree for the [Ag_6PPy]-5 and [Ag_6PPy]-10 systems.

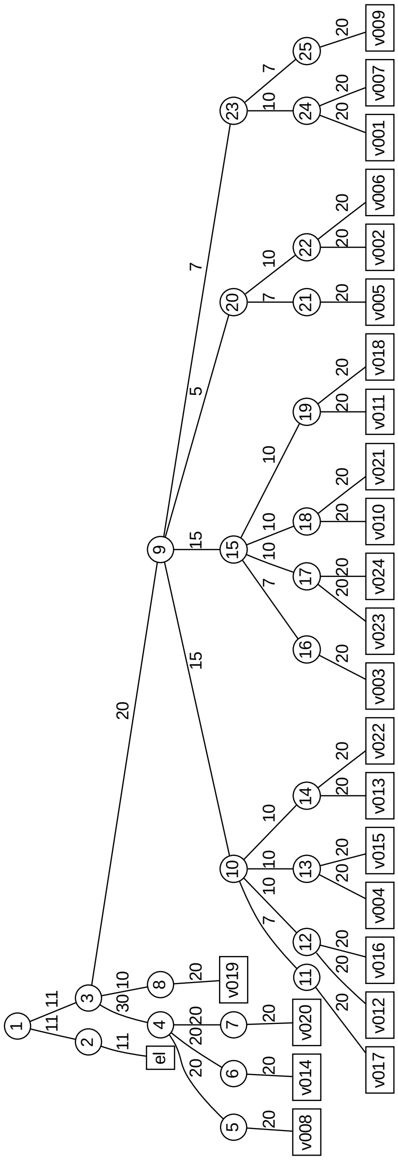

Figure S3: ML-MCTDH tree for the $[Ag_6PPy]$-20 system.

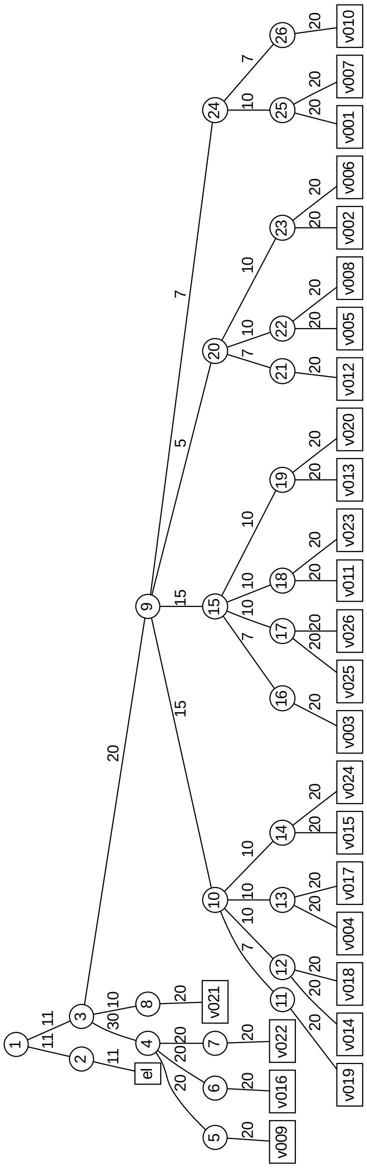

Figure S4: ML-MCTDH tree for the $[Ag_6PPy]$-30 system.

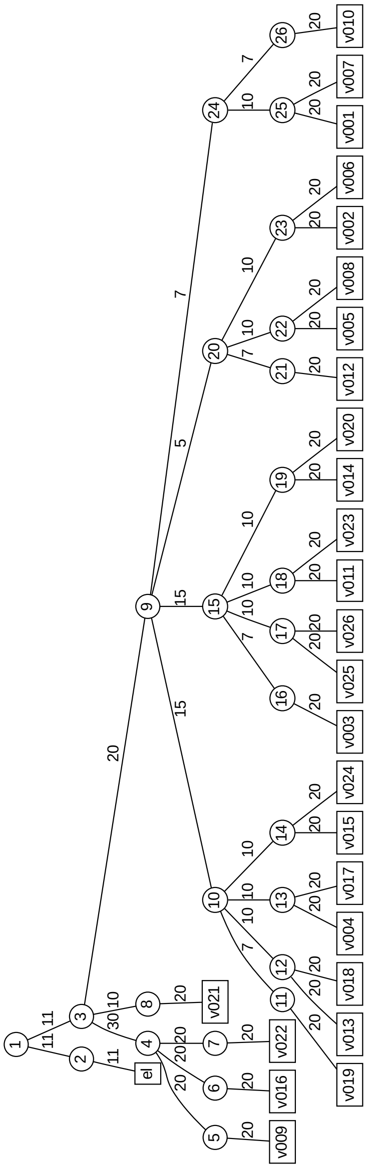

Figure S5: ML-MCTDH tree for the [Ag_6PPy]-40, [Ag_6PPy]-45 and [Ag_6PPy]-50 systems.

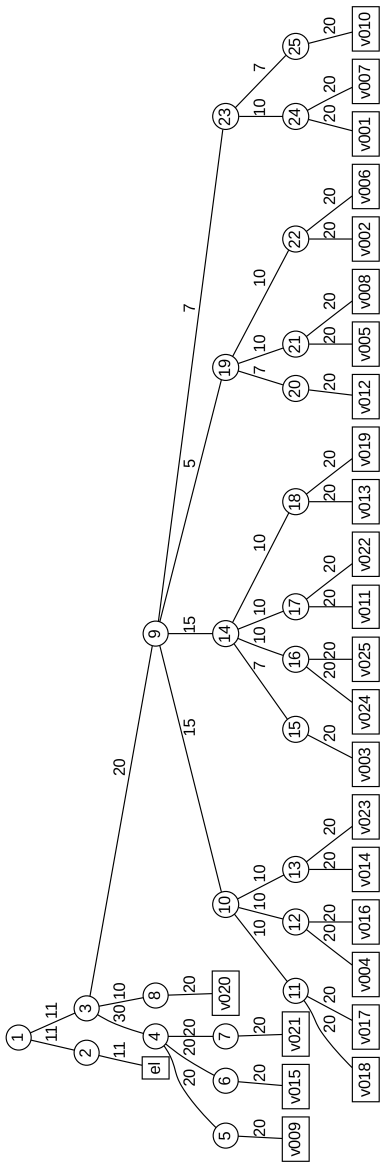

Figure S6: ML-MCTDH tree for the [Ag_6PPy]-60 system.

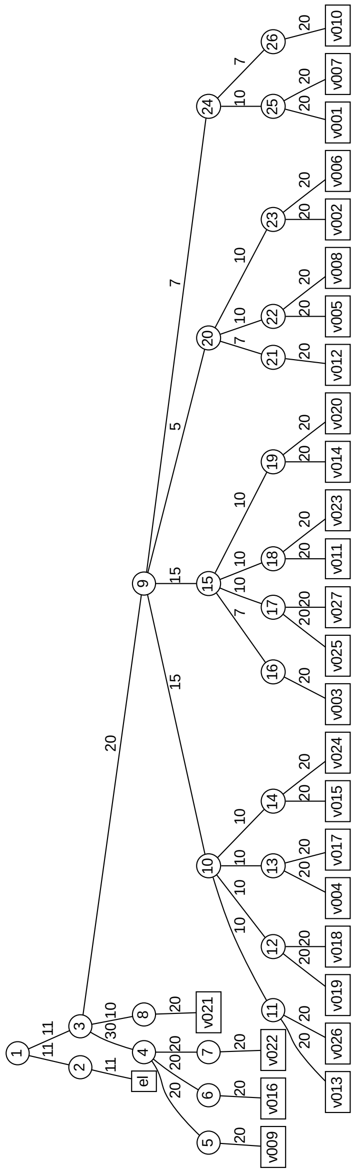

Figure S7: ML-MCTDH tree for the [Ag_6PPy]-75 system.

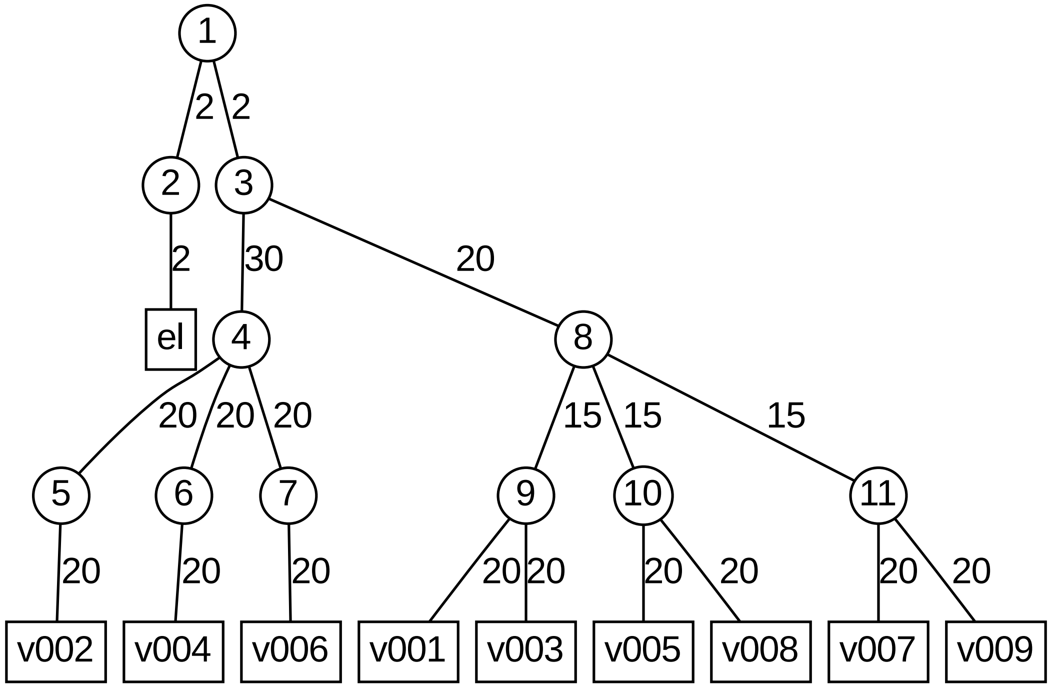

Figure S8: ML-MCTDH tree for all [Ag_6CPy]+$\vec{E}$ systems.

2.2 [Ag_{20}V,SPy]+$\vec{E}$

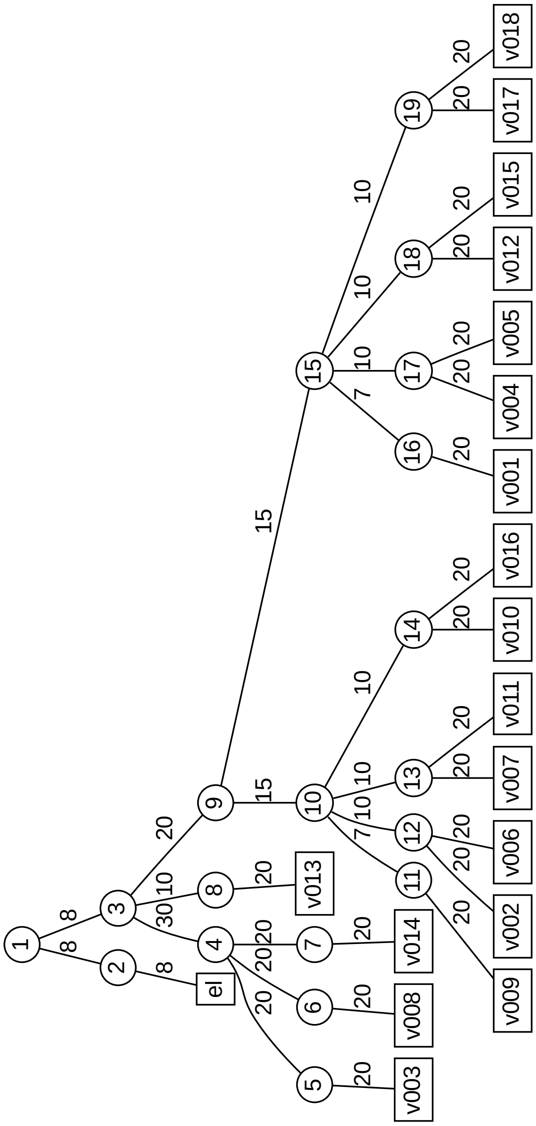

Figure S9: ML-MCTDH tree for all [Ag_{20}VPy]+$\vec{E}$ systems.

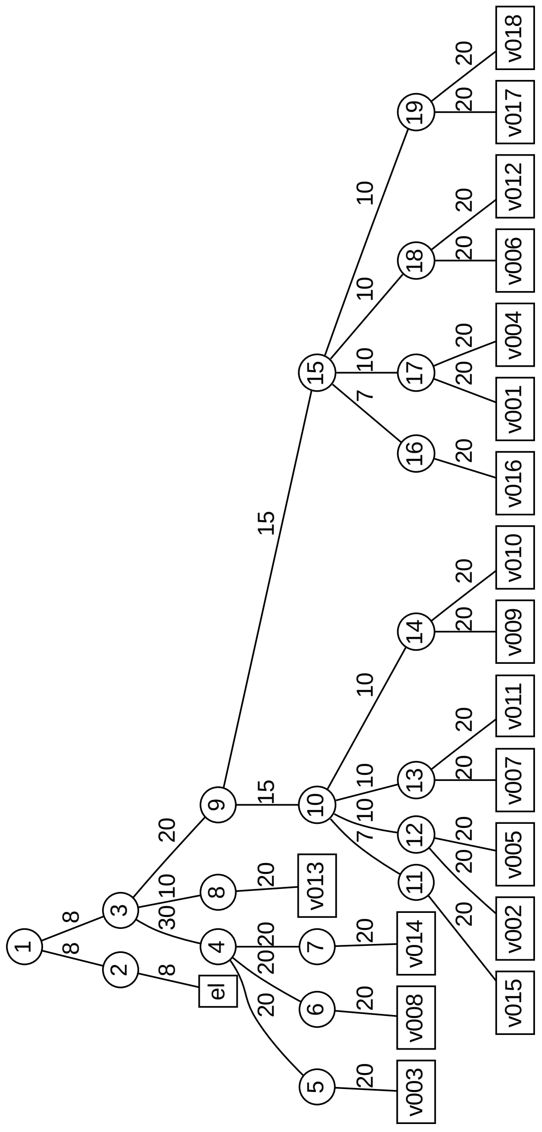

Figure S10: ML-MCTDH tree for all $[Ag_{20}SPy]+\vec{E}$ systems.

3 Normal Modes of Pyridine

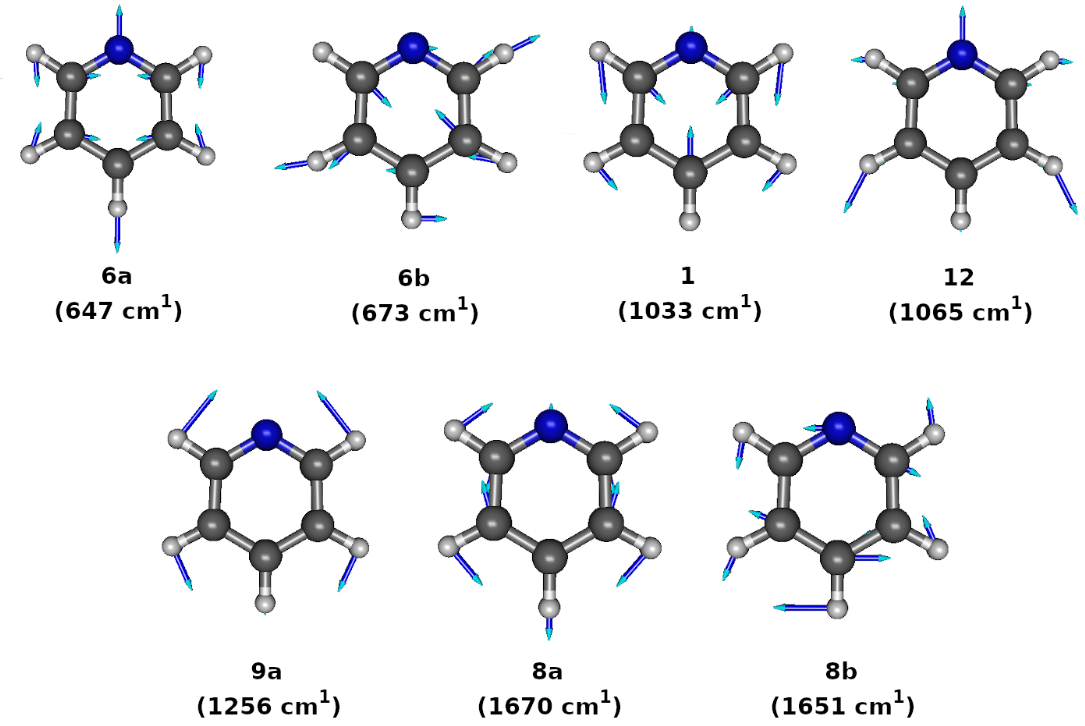

Figure S11: Relevant Py Normal Modes for EC-SERS Spectra. Wavenumbers from calculations for isolated Py at the CAM-B3LYP/LANL2DZ/PCM(H_2O) level of theory.

4 TD-DFT Excited States

4.1 Excited States for [Ag_6P,CPy] Fragments

Table S1: Energies and Oscillator Strengths for the fist 5 excited states of the electric field-free [Ag_6P,CPy]+0 and Py fragments, at the CAM-B3LYP/LanL2DZ/PCM(H_2O) Equilibrium level of theory.

Pyridine [Ag_6PPy]+0			Pyridine [Ag_6CPy]+0		
State	Vertical Energy (eV)	Oscillator Strength	State	Vertical Energy (eV)	Oscillator Strength
S1	4.92	0.01	S1	4.92	0.01
S2	5.39	0.00	S2	5.39	0.00
S3	5.61	0.08	S3	5.61	0.08
S4	6.52	0.05	S4	6.52	0.05
S5	7.12	0.77	S5	7.12	0.77

Table S2: Energies and Oscillator Strengths for the fist 10 excited states of the electric field-free [Ag_6P,CPy]+0 complex, at the CAM-B3LYP/LanL2DZ/PCM(H_2O) Equilibrium level of theory.

Silver Cluster [Ag_6PPy]+0			Silver Cluster [Ag_6CPy]+0		
State	Vertical Energy (eV)	Oscillator Strength	State	Vertical Energy (eV)	Oscillator Strength
S1	2.39	1.80	S1	2.39	1.81
S2	2.40	1.99	S2	2.40	1.99
S3	2.43	0.06	S3	2.43	0.05
S4	2.76	0.29	S4	2.76	0.29
S5	2.79	0.13	S5	2.79	0.13
S6	2.84	0.05	S6	2.84	0.04
S7	2.91	0.00	S7	2.91	0.00
S8	2.96	0.00	S8	2.96	0.00
S9	4.04	2.09	S9	4.04	2.09
S10	4.27	0.00	S10	4.27	0.00

4.2 Excited States for [Ag_{20}V,SPy] Fragments

Table S3: Energies and Oscillator Strengths for the first 5 excited states of the electric field-free [Ag_{20}V,SPy]+0 and Py fragments, at the LanL2DZ/CAM-B3LYP level of theory.

Pyridine [Ag_{20}VPy]+0			Pyridine [Ag_{20}SPy]+0		
State	Vertical Energy (eV)	Oscillator Strength	State	Vertical Energy (eV)	Oscillator Strength
S1	4.73	0.01	S1	4.76	0.01
S2	5.09	0.00	S2	5.10	0.00
S3	5.68	0.03	S3	5.68	0.03
S4	6.63	0.01	S4	6.63	0.02
S5	7.58	0.55	S5	7.58	0.55

Table S4: Energies and Oscillator Strengths for the bright PL excited states of the electric field-free [Ag_{20}V,SPy]+0 complexes, at the LanL2DZ/CAM-B3LYP level of theory.

Silver Cluster [Ag_{20}VPy]+0			Silver Cluster [Ag_{20}SPy]+0		
State	Vertical Energy (eV)	Oscillator Strength	State	Vertical Energy (eV)	Oscillator Strength
S58	3.77	1.79	S58	3.76	2.38
S59	3.78	1.85	S59	3.78	2.15
S60	3.79	1.85	S60	3.78	2.13

5 LVC Diabatic States

5.1 Definitions for LVC Diabatic States

Table S5: Definition of Diabatic States for [Ag_NTPy] systems (N=6,20 and T=P,C for N=6 and T=V,S for N=20). For [Ag_6TPy] complexes symmetry imposes that the transition dipole moment is oriented along a single axis as specified in the table while each CT and LE can only interacts only with PL state of a particular polarization as indicated by its label. For [Ag_{20}TPy] complexes only PL states are included due to the very large number of dark LE states.

[Ag_6PPy]+$\vec{E}$		[Ag_{20}VPy]+$\vec{E}$	
Diabatic State	Definition	Diabatic State	Definition
PL−X	S1 Ag_6	PL_1	S58 Ag_{20}
PL−Z	S2 Ag_6	PL_2	S59 Ag_{20}
LE−X_1	S3 Ag_6	PL_3	S60 Ag_{20}
LE−X_2	S4 Ag_6	$CT0_1$	HOMO Ag_{20} → LUMO Py
LE−Z_1	S5 Ag_6	$CT1_1$	HOMO Ag_{20} → LUMO+1 Py
LE−Z_2	S6 Ag_6	$CT0_2$	HOMO-1 Ag_{20} → LUMO Py
LE−Y	S8 Ag_6	$CT0_3$	HOMO-2 Ag_{20} → LUMO Py
PL−Y	S9 Ag_6	$CT1_2$	HOMO-1 Ag_{20} → LUMO+1 Py
CT0−Z	HOMO Ag_6 → LUMO Py		
CT1−Y	HOMO Ag_6 → LUMO+1 Py		
CT0−X	HOMO-1 Ag_6 → LUMO Py		
[Ag_6CPy]+$\vec{E}$		[Ag_{20}SPy]+$\vec{E}$	
Diabatic State	Definition	Diabatic State	Definition
PL−X	S9 Ag_6	PL_1	S58 Ag_{20}
CT0−X	HOMO-1 Ag_6 → LUMO Py	PL_2	S59 Ag_{20}
		PL_3	S60 Ag_{20}
		$CT0_1$	HOMO Ag_{20} → LUMO Py
		$CT1_1$	HOMO Ag_{20} → LUMO+1 Py
		$CT0_2$	HOMO-1 Ag_{20} → LUMO Py
		$CT0_3$	HOMO-2 Ag_{20} → LUMO Py
		$CT1_2$	HOMO-1 Ag_{20} → LUMO+1 Py

5.2 Transition Dipole Moments for LVC Diabatic States

Table S6: Transition Dipole Moments for the Diabatic States for the [Ag_6PPy]+0 and [Ag_6CPy]+0 systems.

[Ag_6PPy]+0			
Diabatic State	X Component	Y Component	Z Component
PL-X	5.51	0.00	0.00
PL-Z	0.00	0.00	5.66
LE-X_1	0.90	0.00	0.00
LE-X_2	2.19	0.00	0.00
LE-Z_1	0.00	0.00	1.45
LE-Z_2	0.00	0.00	0.73
LE-Y	0.00	0.07	0.00
PL-Y	0.00	-4.51	0.00
CT0-Z	0.00	0.00	-0.08
CT1-Y	0.00	0.01	0.00
CT0-X	0.03	0.00	0.00
[Ag_6CPy]+0			
PL-X	-4.53	0.00	0.00
CT0-X	-0.05	0.00	0.00

Table S7: Transition Dipole Moments for the Diabatic States for the [Ag_{20}VPy]+0 and [Ag_{20}SPy]+0 systems.

[Ag_{20}VPy]+0			
Diabatic State	X Component	Y Component	Z Component
PL_1	0.00	-0.02	4.25
PL_2	3.88	2.52	0.01
PL_3	-2.51	3.86	0.01
$CT0_3$	-0.01	0.00	0.00
$CT1_2$	0.02	0.01	0.00
$CT0_3$	-0.01	0.02	0.12
$CT0_1$	0.03	0.02	0.01
$CT0_2$	0.01	-0.01	0.00
[Ag_{20}SPy]+0			
PL_1	-0.17	0.00	-5.32
PL_2	4.66	0.00	-0.17
PL_3	0.00	-4.72	0.00
$CT0_3$	0.00	-0.08	0.00
$CT1_2$	0.00	-0.02	0.00
$CT0_3$	0.02	0.00	-0.22
$CT0_1$	0.00	0.00	-0.23
$CT0_2$	0.02	0.00	0.20

6 Natural Transition Orbitals

6.1 Natural Transition Orbitals for [Ag_6P,C]

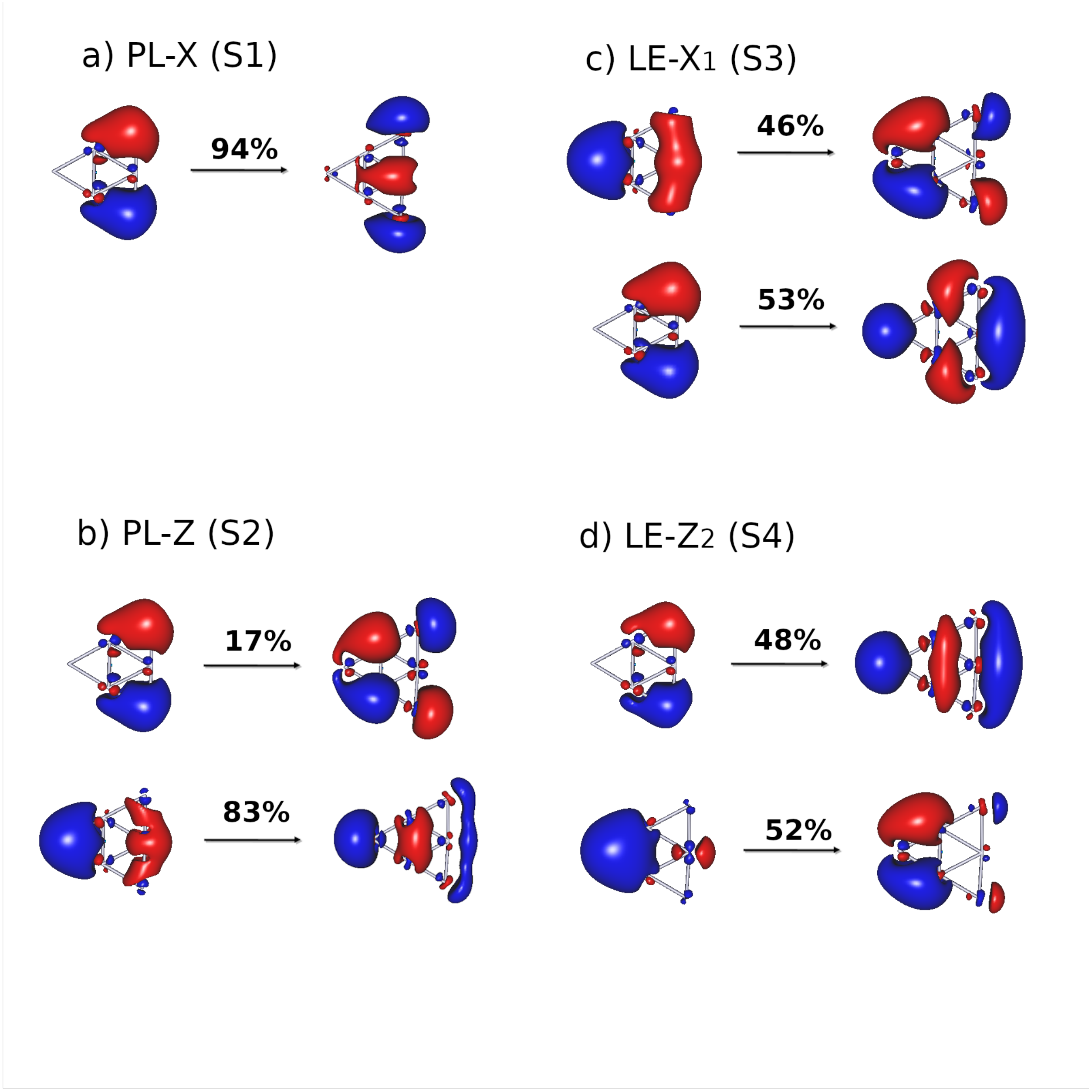

Figure S12: Most relevant Natural Transition Orbitals for PL-X (S1) (a), PL-Z (S2) (b), LE-X_1 (S3) (c) and LE-Z_2 (S4) (d) for the [Ag_6] moiety in the ground state minimum of the [Ag_6PPy] complex, CAM-B3LYP/LANL2DZ/PCM(H_2O) Equilibrium level of theory, surface isovalue of 0.0018.

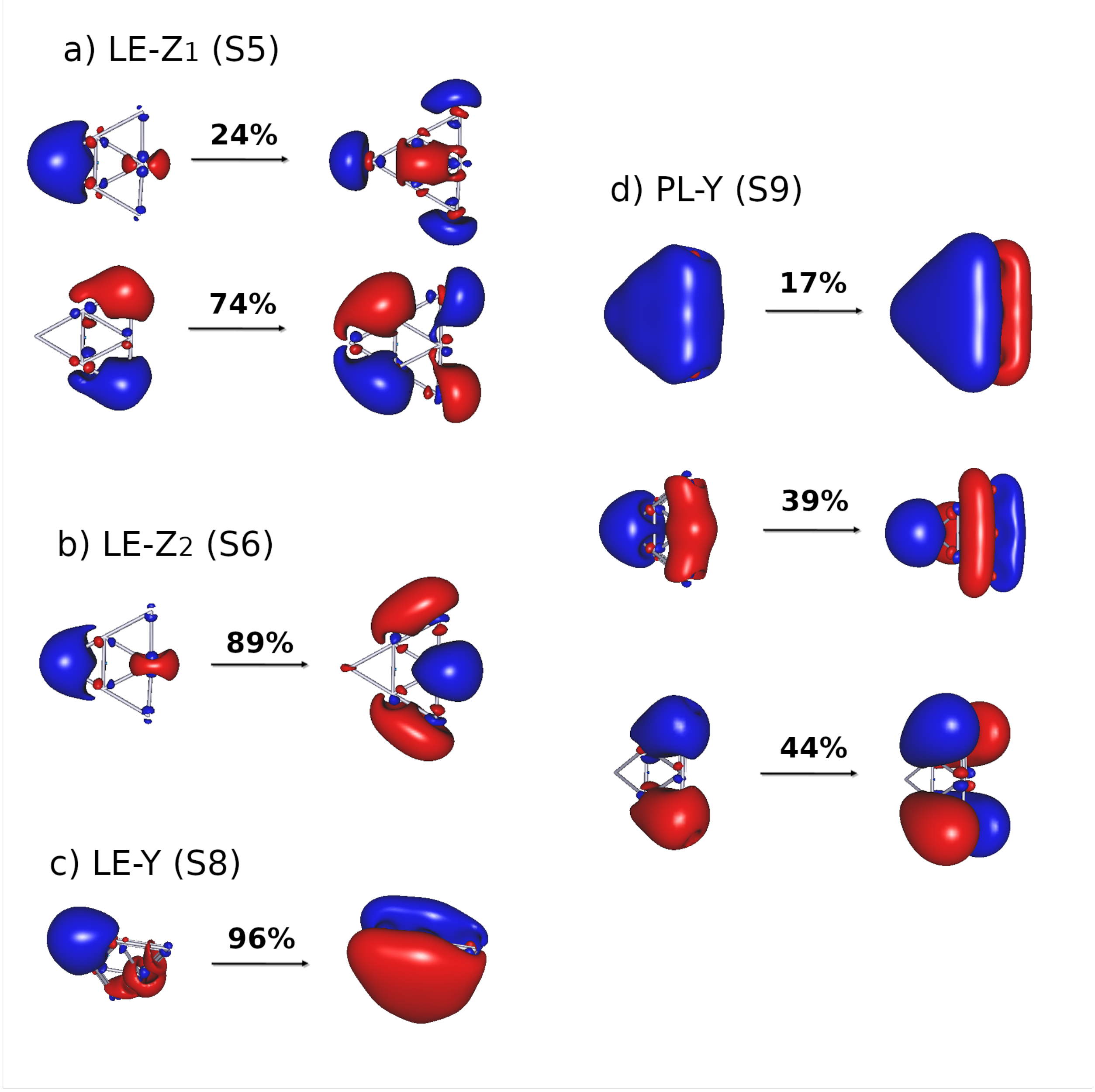

Figure S13: Most relevant Natural Transition Orbitals for LE-Z_1 (S5) (a), LE-Z_2 (S6) (b), LE-Y (S8) (c) and PL-Y (S9) (d) for the [Ag_6] moiety in the ground state minimum of the [Ag_6PPy] complex, CAM-B3LYP/LANL2DZ/PCM(H_2O) level of theory, surface isovalue of 0.0018.

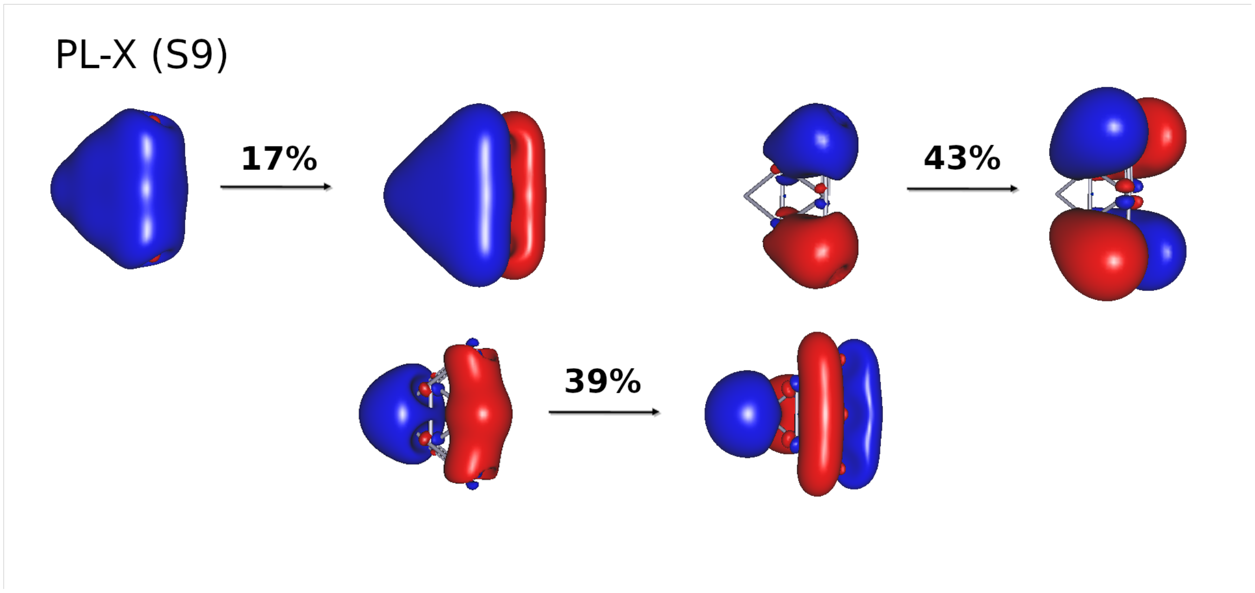

Figure S14: Most relevant Natural Transition Orbitals for PL-X (S9) for the [Ag_6] moiety in the ground state minimum of [Ag_6CPy] complex, CAM-B3LYP/LANL2DZ/PCM(H_2O) level of theory, surface isovalue of 0.0018.

6.2 Natural Transition Orbitals for [Ag_{20}V,S]

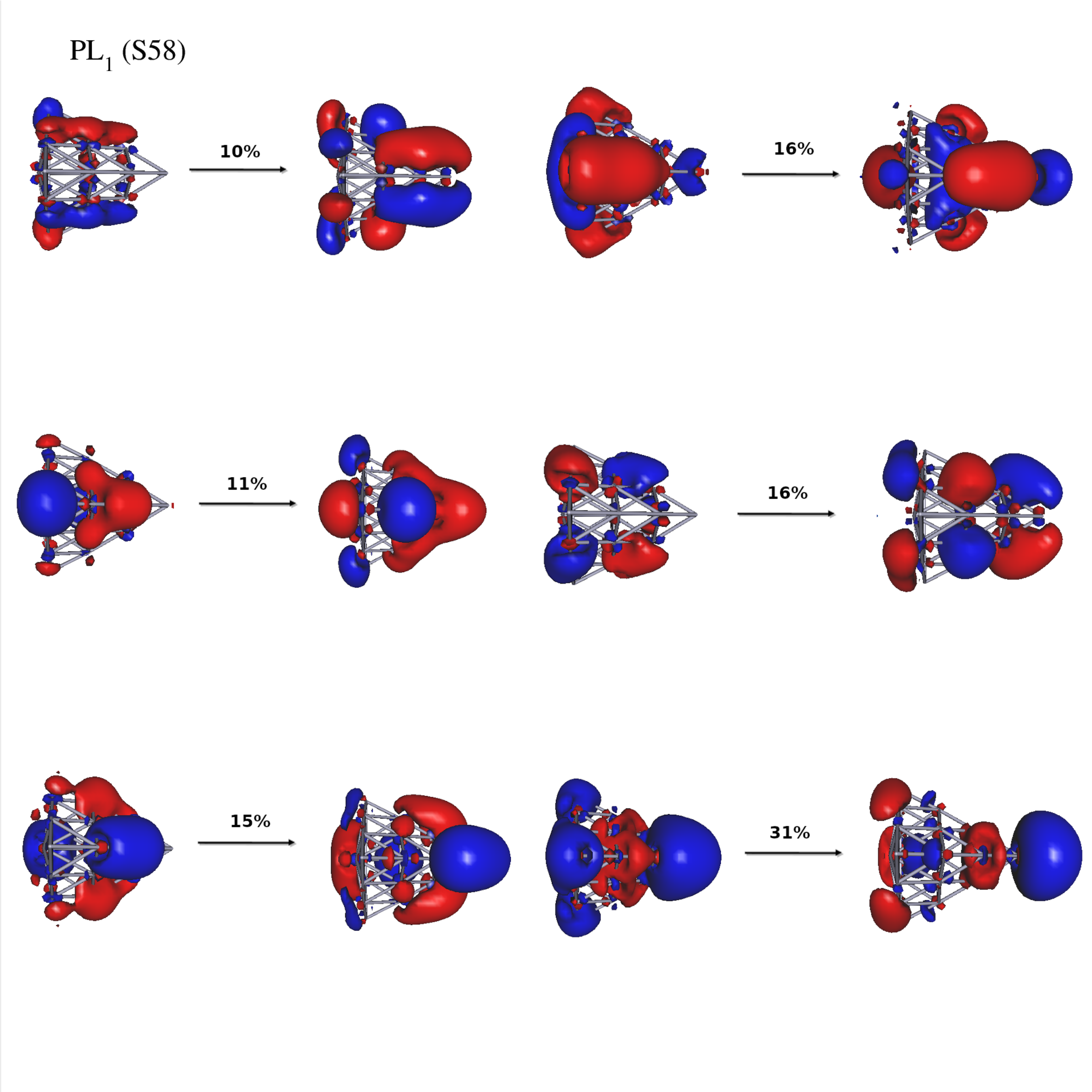

Figure S15: Most relevant Natural Transition Orbitals for PL_1 (S58) for the [Ag_{20}] moiety in the ground state structure of the [Ag_{20}SPy] complex, CAM-B3LYP/LANL2DZ level of theory, surface isovalue of 0.0016.

PL$_2$ (S59)

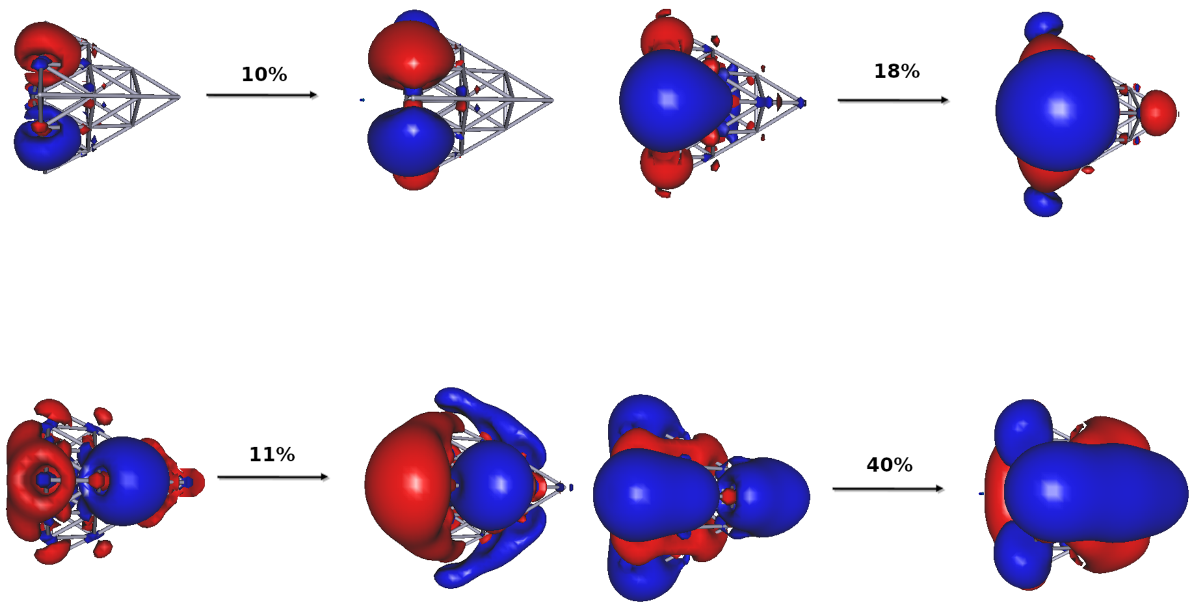

Figure S16: Most relevant Natural Transition Orbitals for PL$_2$ (S59) for the [Ag$_{20}$S] moiety in the ground state structure of the [Ag$_{20}$SPy] complex, CAM-B3LYP/LANL2DZ level of theory, surface isovalue of 0.0016.

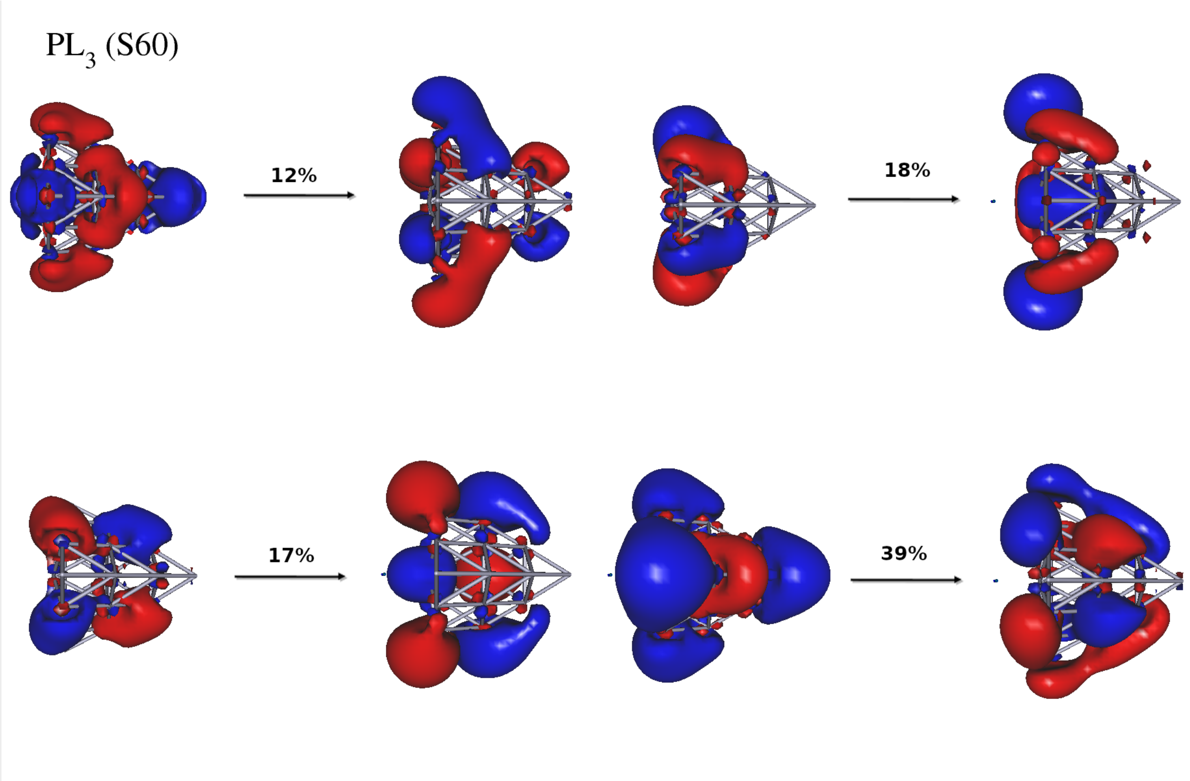

Figure S17: Most relevant Natural Transition Orbitals for PL_3 (S60) for the [$Ag_{20}S$] moiety in the ground state structure of the [$Ag_{20}SPy$] complex, CAM-B3LYP/LANL2DZ level of theory, surface isovalue of 0.0016.

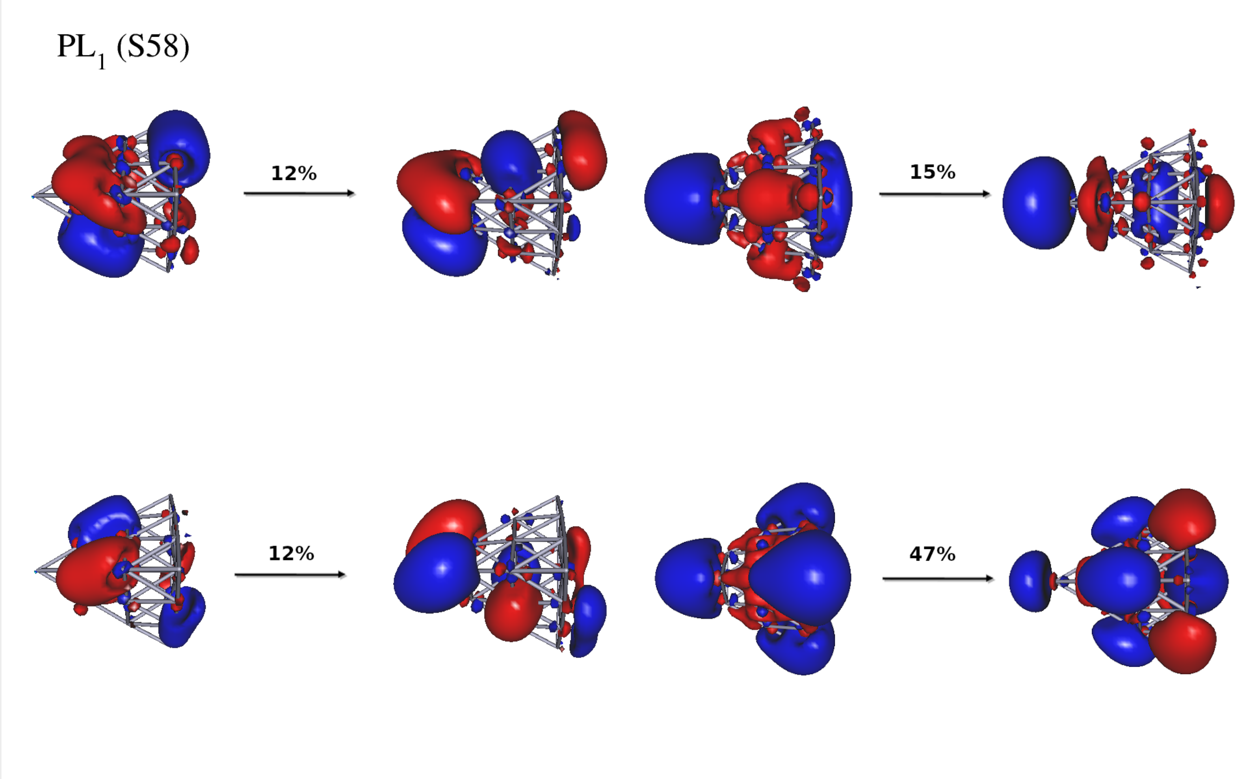

Figure S18: Most relevant Natural Transition Orbitals for PL_1 (S58) for the [Ag_{20}] moiety in the ground state structure of the [Ag_{20}VPy] complex, CAM-B3LYP/LANL2DZ level of theory, surface isovalue of 0.0016.

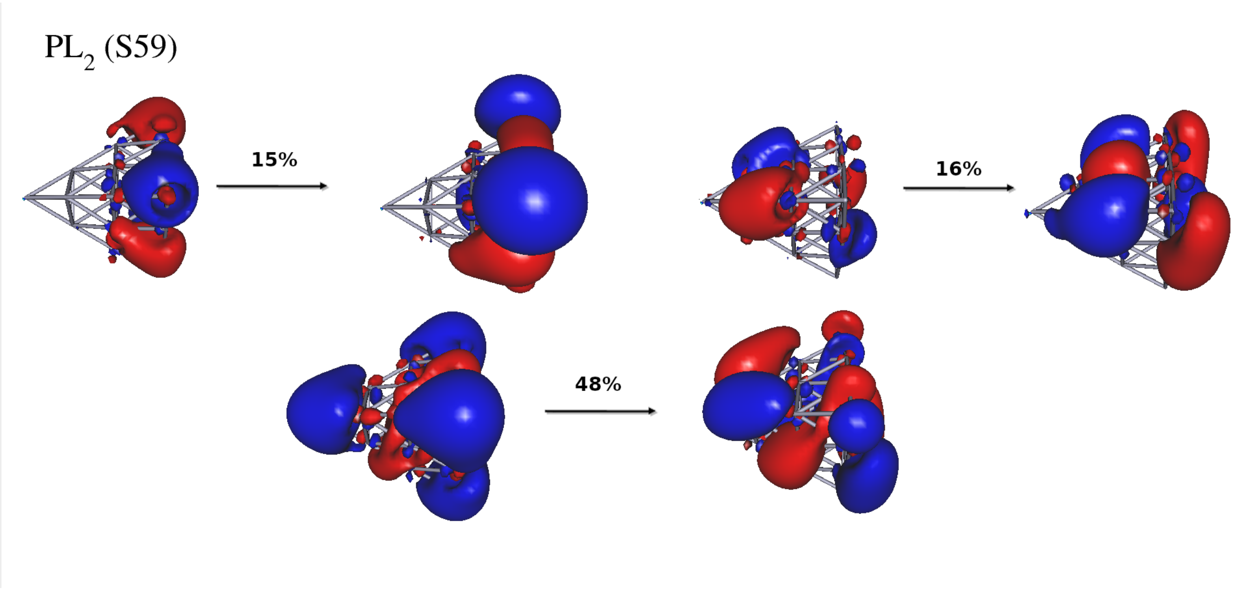

Figure S19: Most relevant Natural Transition Orbitals for PL_2 (S59) for the [Ag_{20}V] moiety in the ground state structure of the [Ag_{20}Py] complex, CAM-B3LYP/LANL2DZ level of theory, surface isovalue of 0.0016.

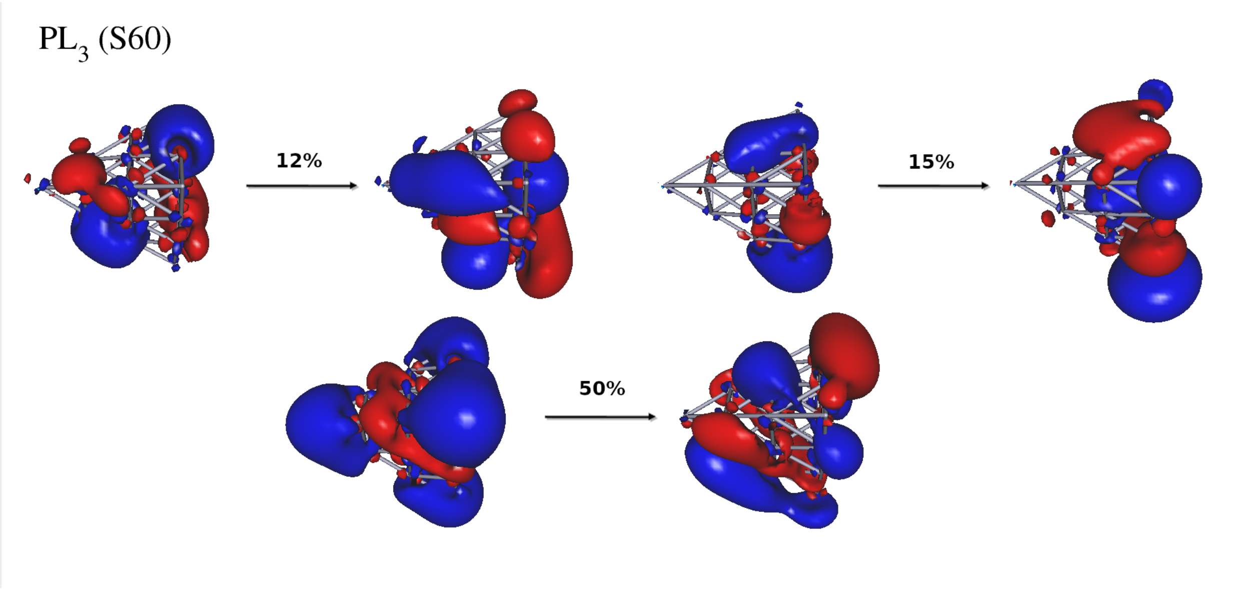

Figure S20: Most relevant Natural Transition Orbitals for PL_3 (S60) for the [Ag_{20}] moiety in the ground state structure of the [Ag_{20}VPy] complex, CAM-B3LYP/LANL2DZ level of theory, surface isovalue of 0.0016.

7 Dependence of the LVC Hamiltonian with the Electric Field

7.1 PL, LE and CT Diagonal terms

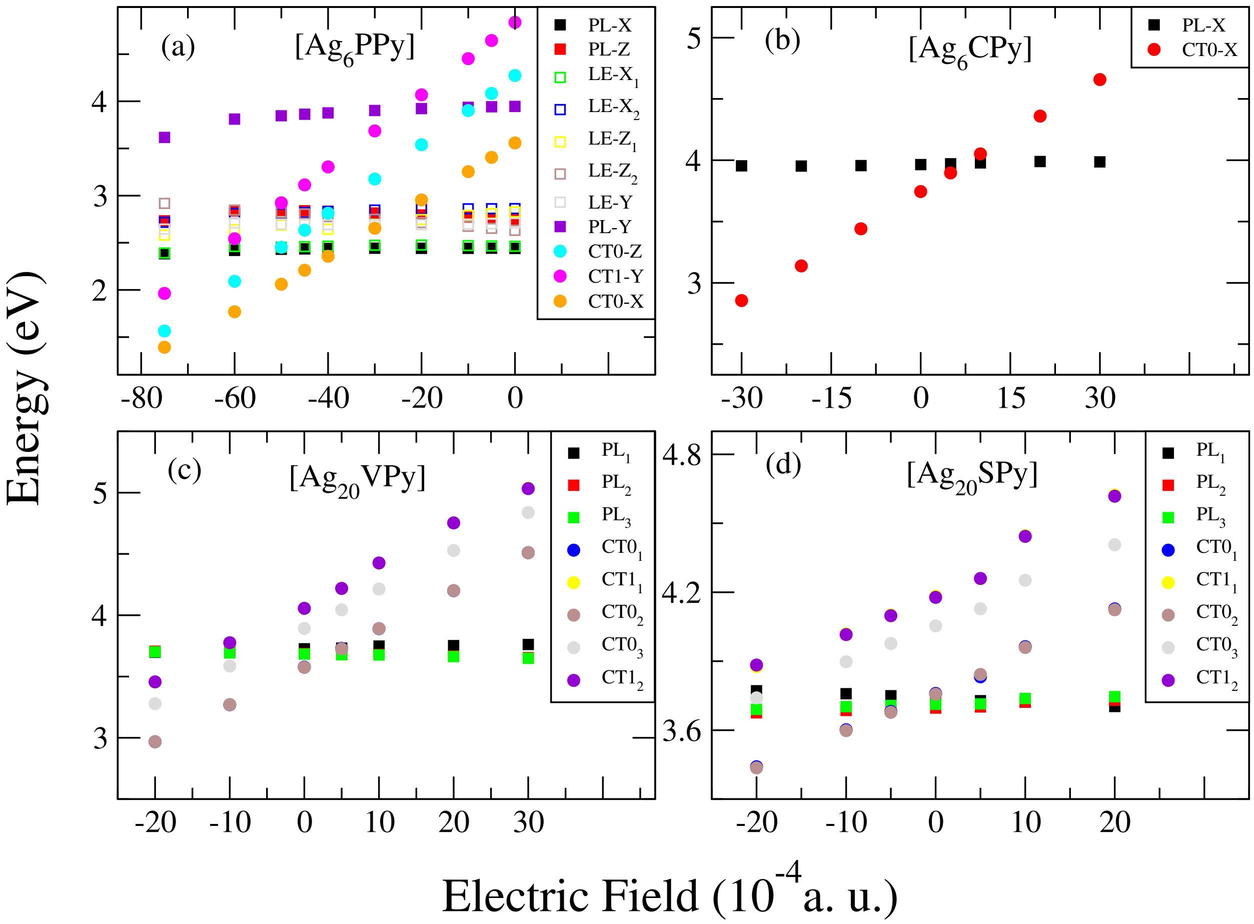

Figure S21: Diagonal E_{ii}^{0} terms for all PL, LE and CT states for all systems.

7.2 PL/CT and LE/CT constant off-diagonal terms

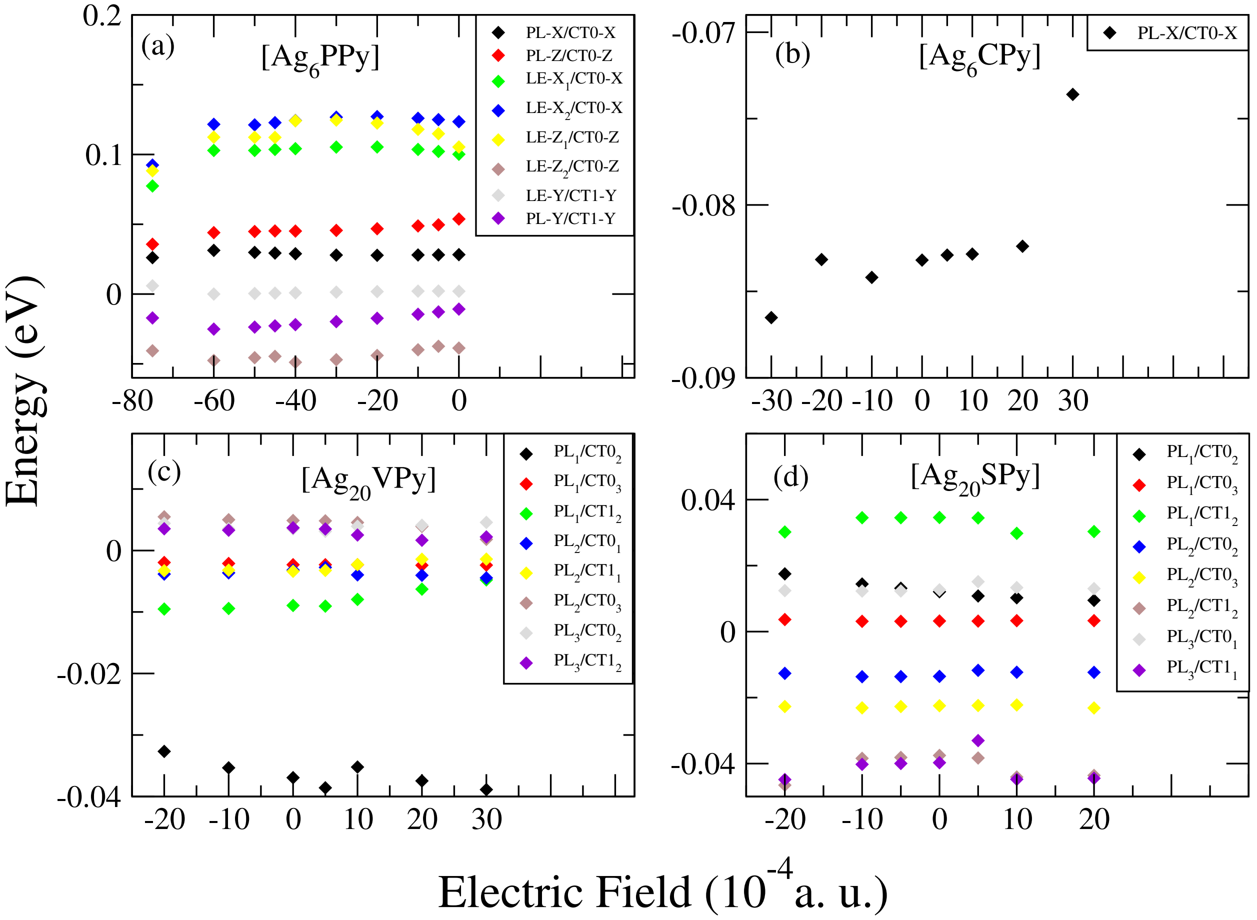

Figure S22: Off-Diagonal E^0_{ij} terms between CT and PL states for all systems. Non-represented couplings for [Ag_6P,CPy] are zero, and a threshold of 0.001 eV has been established for [Ag_{20}V,SPy] systems.

7.3 PL/PL, PL/LE and LE/LE constant off-diagonal terms

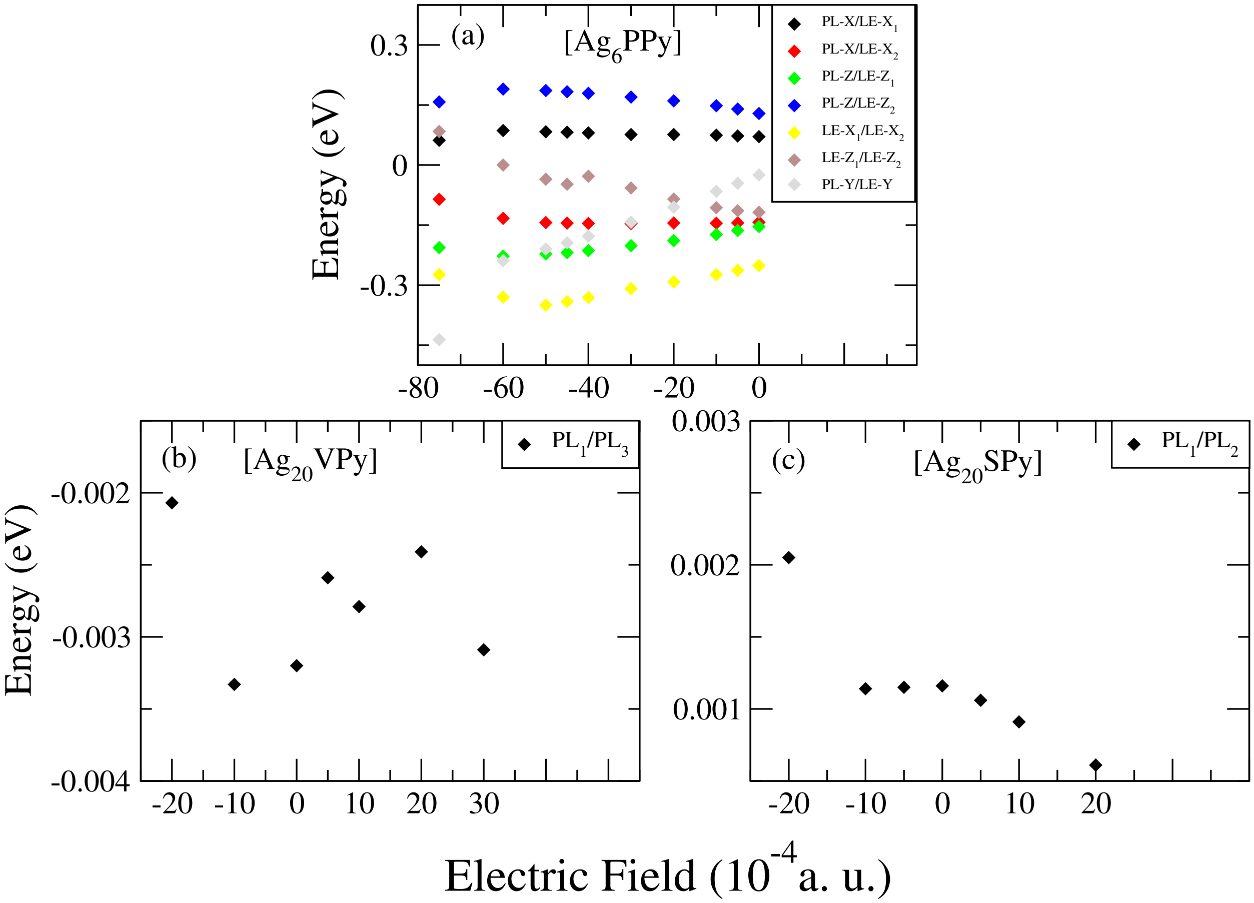

Figure S23: Off-Diagonal E^0_{ij} terms between different PL and LE states for all systems. For [Ag_6PPy] all the terms are zero and have been omitted while for [Ag_6CPy] complexes there is only a PL states and these terms do not exists. For [Ag_{20}V,SPy] systems only those with values larger than 0.001 eV are represented.

7.4 CT-CT constant off-diagonal terms

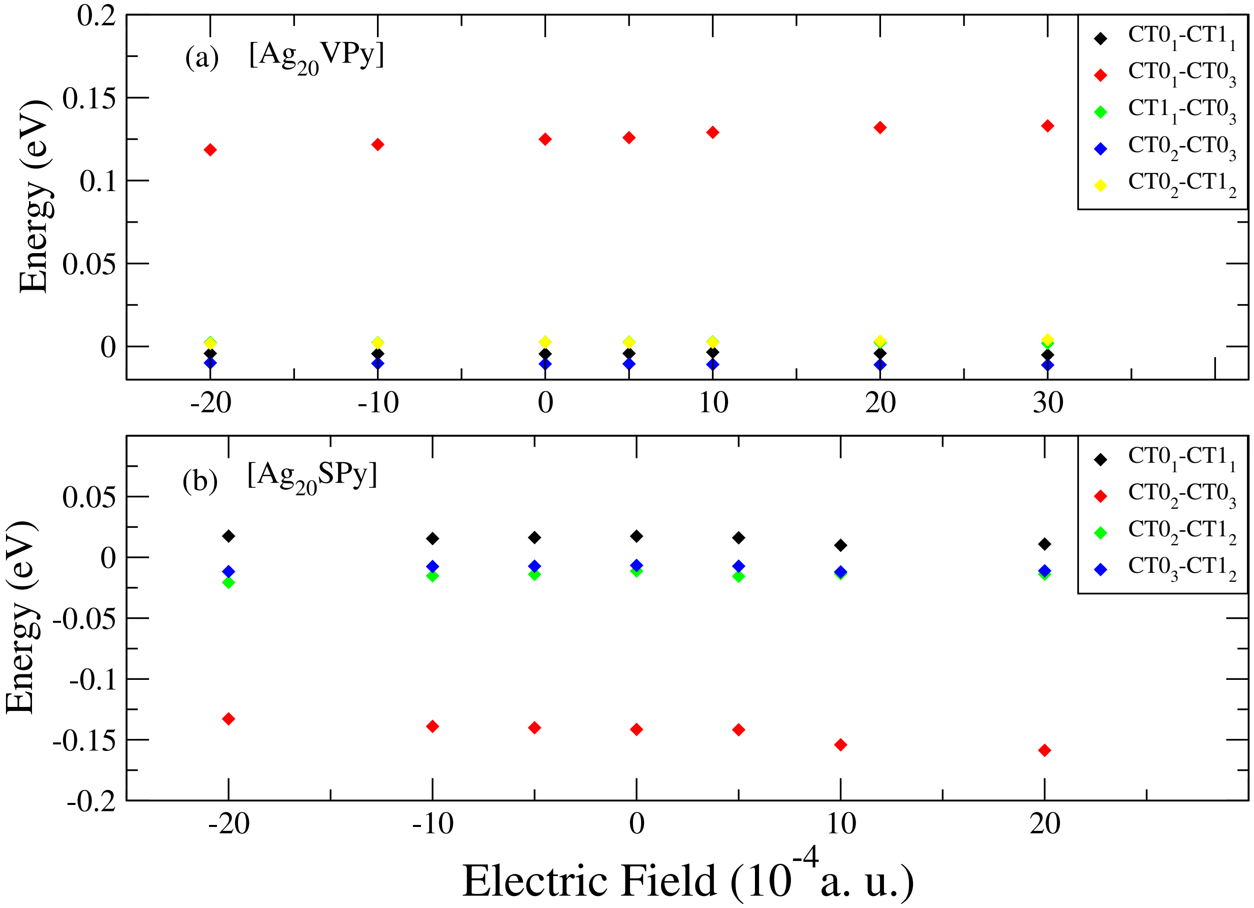

Figure S24: Off-Diagonal terms E^0_{ij} between different CT states for all systems. For [Ag_6P,CPy] systems these terms do not exist or are all zero so they have not been represented. For [Ag_{20}V,SPy] systems only those with values larger than 0.001 eV are represented.

8 Populations Dynamics

8.1 $[Ag_6P,CPy]+\vec{E}$ Wavepacket Propagations

Figures S25-S27 shows the time evolution of $[Ag_6PPy]+\vec{E}$ diabatic state populations photoexciting to PL-Z, PL-X and PL-Y, respectively. Due to the strong electronic coupling between states of the same set, the population oscillates rapidly between PL and LE states. As discussed in the main text, the population of states with different polarization (X, Y or Z) is very small for each set. The tuning and detuning process is also observed. Focusing on PL-X photoexcitation (Figure S26), from E=-10 (10^{-4} a. u.), the CT0-X state gains population as its energy starts getting closer to the energy of PL-X, gradually increasing until E=-45 10^{-4} a. u., where the population gets to its maximum. The detuning occurs extremely fast as decreasing the field to E=-50,60 (10^{-4} a. u.) rapidly diminishes the population to about 18% and 4%, respectively, and is practically vanishing as the field becomes more negative due to the energy of the CT state being smaller and out of resonance.

The time-evolution of the electronic populations after an excitation to PL-Y, reported in Figure S25, shows that other states can get populated, specially during the maximum energy overlap at E=-30 (10^{-4} a. u.), where the population of CT1-Y is stable and the wavepacket shifts to other symmetries CT and PL states. This is due to the vibronic coupling between CT0 and CT1, which allows the population to flow from CT1-Y to CT0-X and/or CT0-Z, and from them, to the PL-X and PL-Z states, respectively. Also, it is worth to notice that fast oscillations between LE-Y and PL-Y get more drastic as CT1-Y gets lower and farther in energy, being E=-75 the most extreme shown case, while at E=0 oscillations are much lower, which shows that as PL states get comparatively higher in energy, the photoexcited wavepackets oscillate faster between them, even though their difference in energy is the same.

For $[Ag_6CPy]+\vec{E}$ complexes (Figure S28) the analysis is simpler because there are only two states involved. As for the $[Ag_6PPy]$, the tuning and detuning processes are perfectly observed, in the range E=30 to -20 (10^{-4} a.u.). The population transfer is very effective, achieving values up to 80%-90% with very steep slopes in the range E=10^{-4} to 0 a.u. and the detuning is again very fast, in perfect analogy with the previous case.

Finally, Figure S28 shows that including the Ag modes simply soften the oscillations between PL and LE states after some time, without affecting remarkably to the total population transfer to the CT states. They also allow to populate PL states of different polarization as they mix through vibronic coupling.

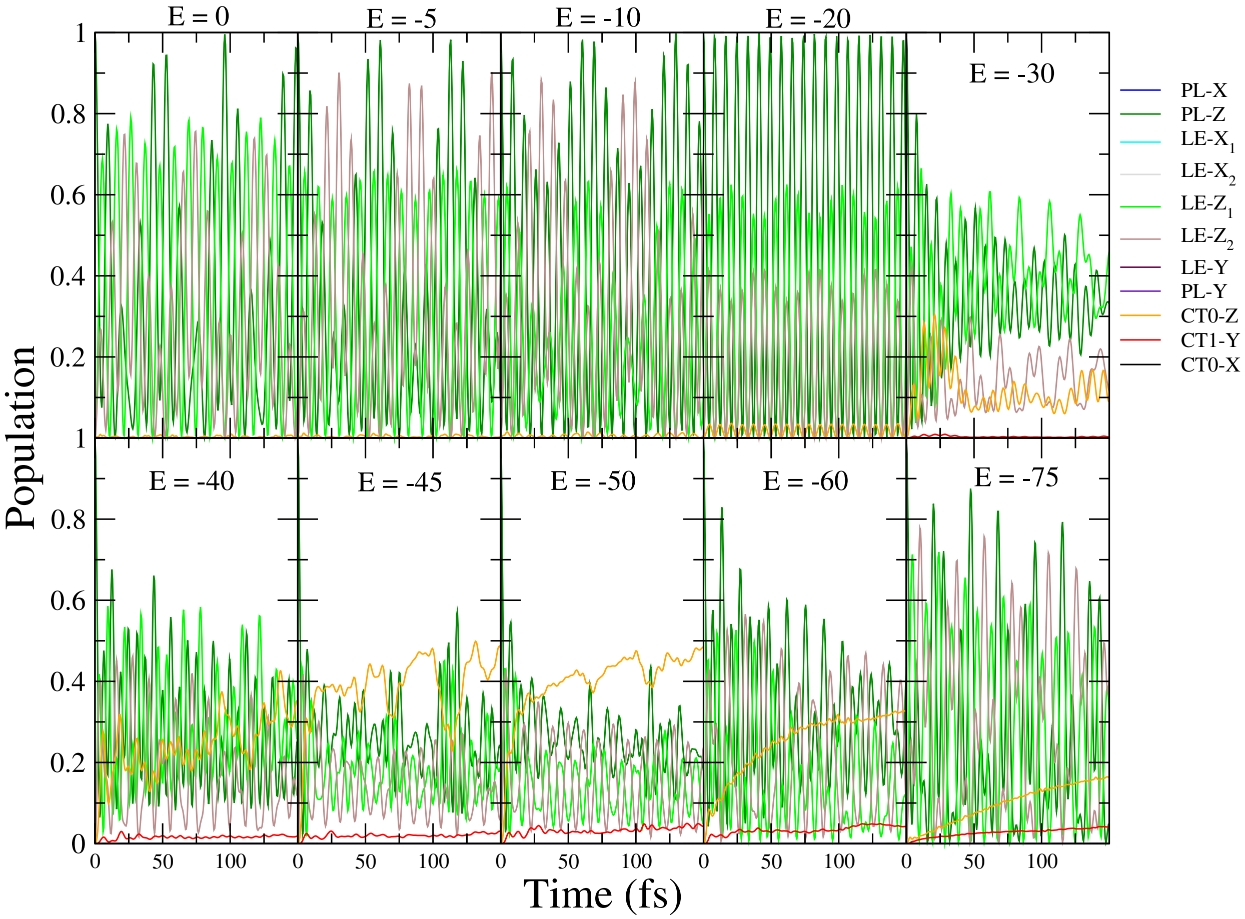

Figure S25: Population dynamics of diabatic states for $[Ag_6PPy]+\vec{E}$ systems photoexciting to state PL-Z. E expressed in 10^{-4} a.u.

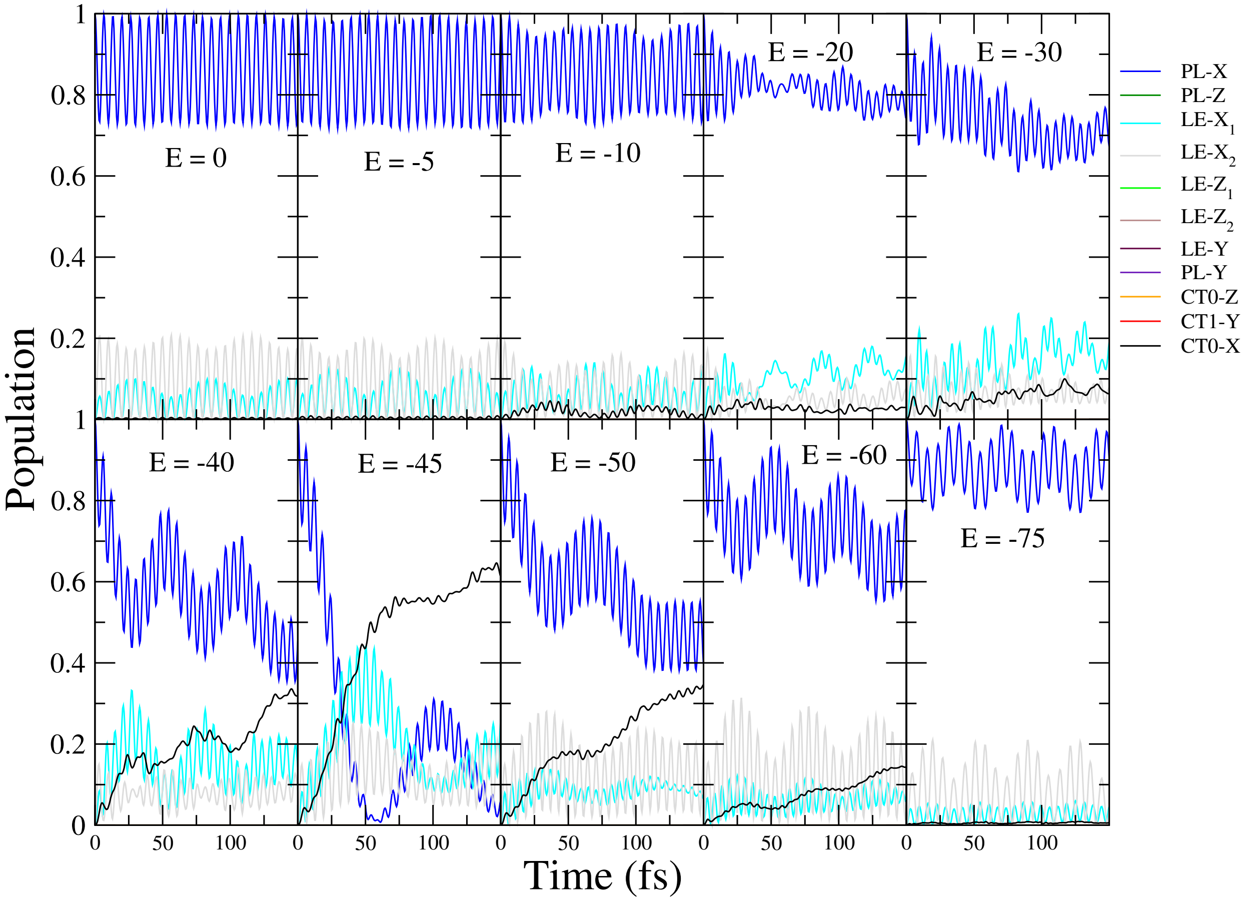

Figure S26: Population dynamics of diabatic states for $[Ag_6PPy]+\vec{E}$ systems photoexciting to state PL-X. E expressed in 10^{-4} a.u.

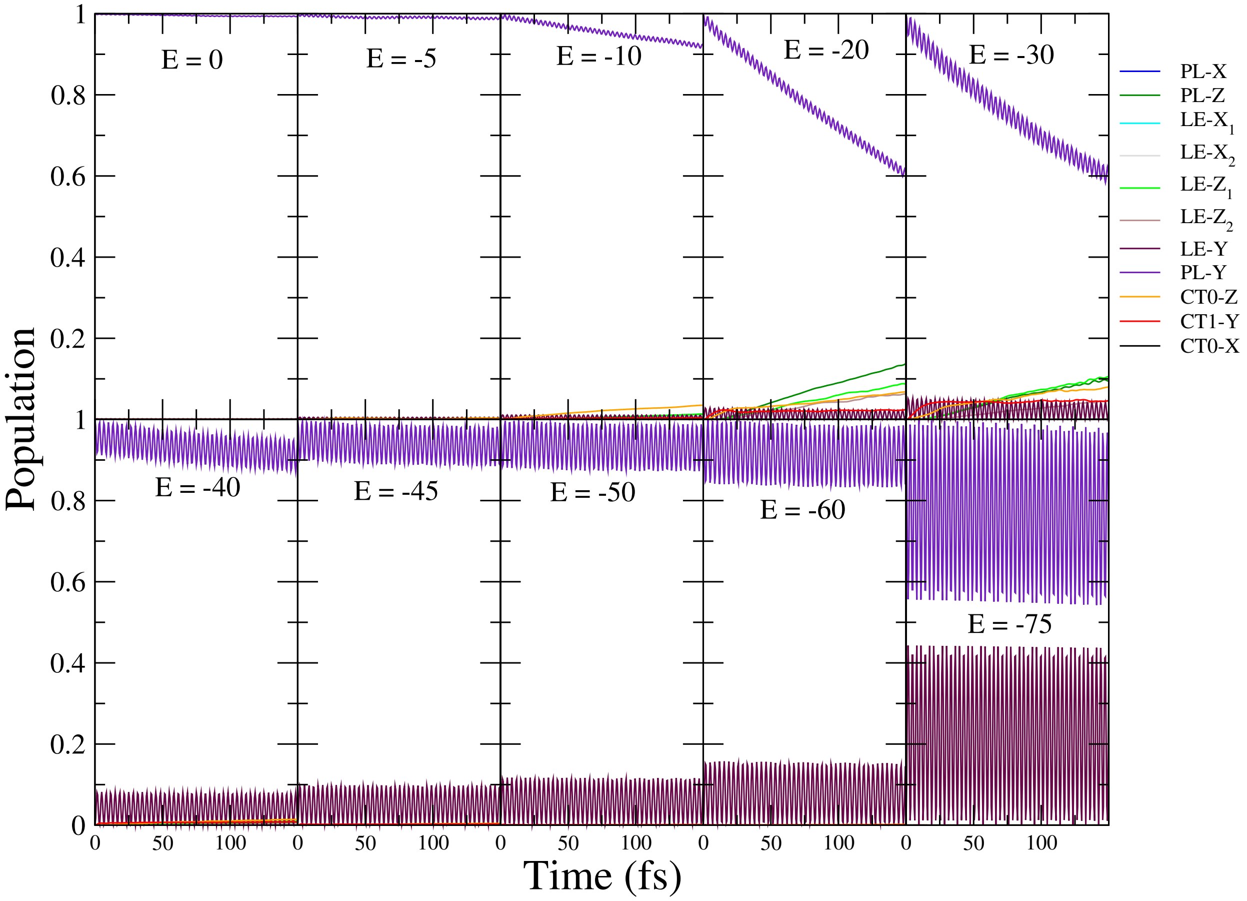

Figure S27: Population dynamics of diabatic states for $[Ag_6PPy]+\vec{E}$ systems photoexciting to state PL-Y. E expressed in 10^{-4} a.u.

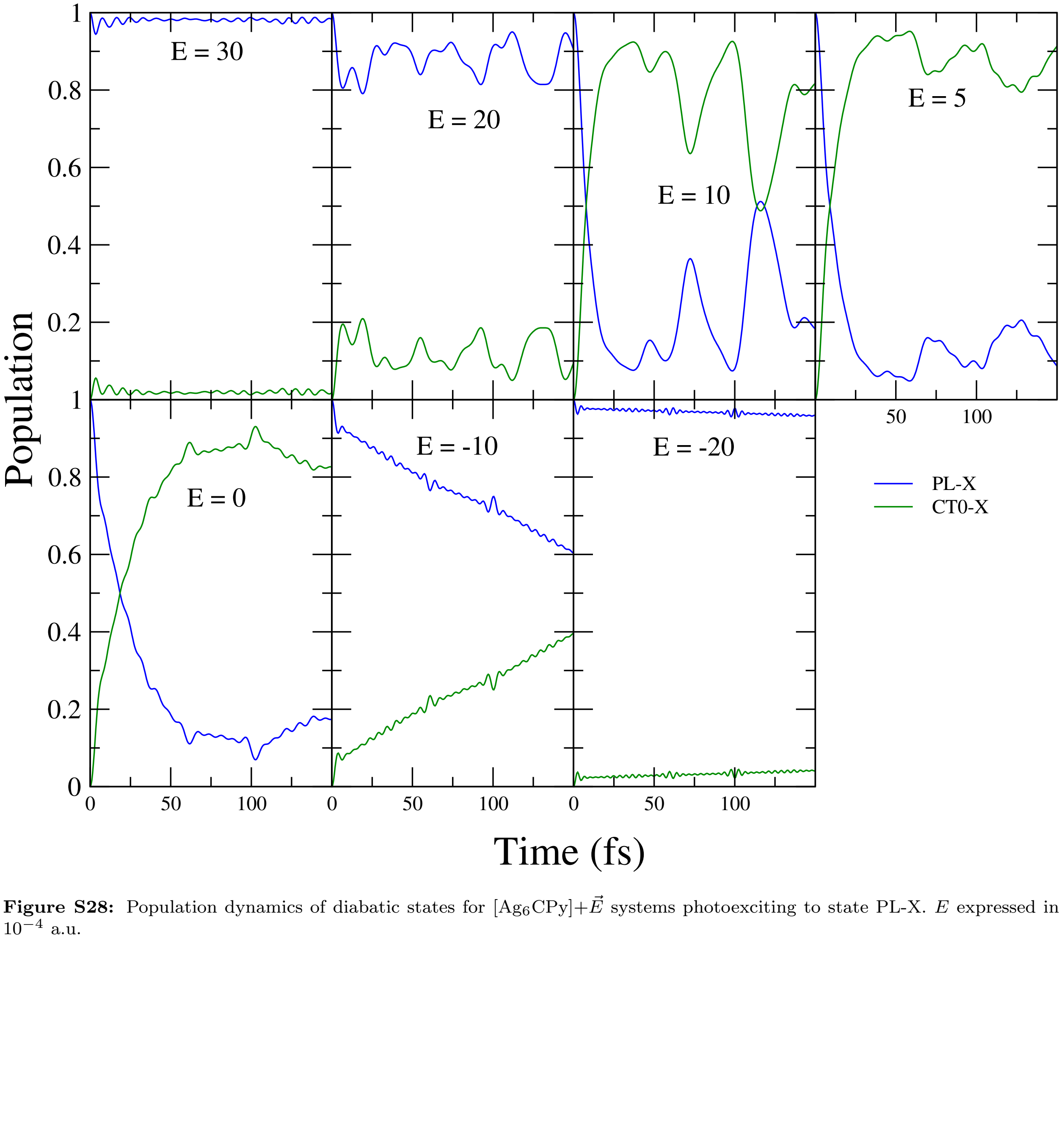

Figure S28: Population dynamics of diabatic states for $[Ag_6CPy]+\vec{E}$ systems photoexciting to state PL-X. E expressed in 10^{-4} a.u.

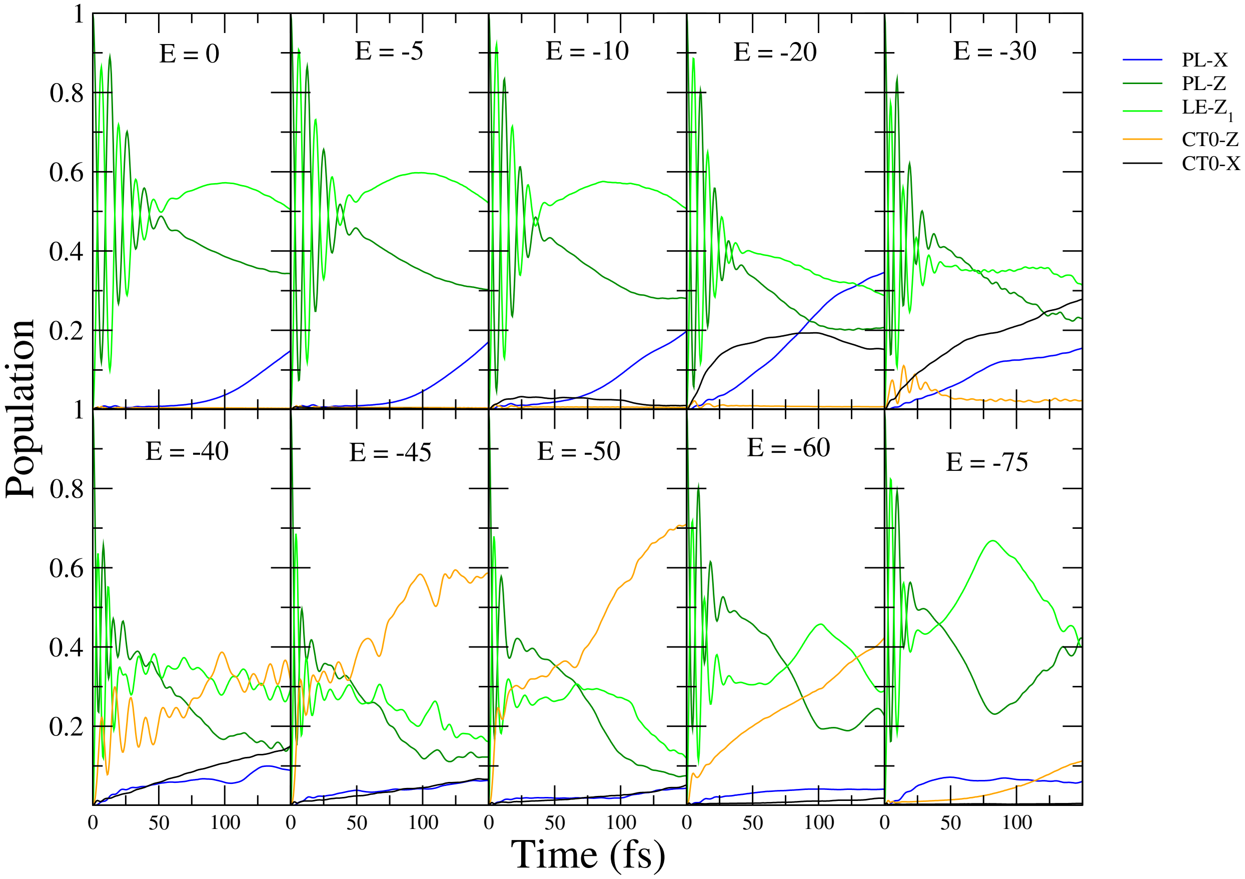

Figure S29: Population dynamics of diabatic states for $[Ag_6PPy]+\vec{E}$ systems photoexciting to state PL-Z and including Normal Modes of the silver cluster. E expressed in 10^{-4} a.u.

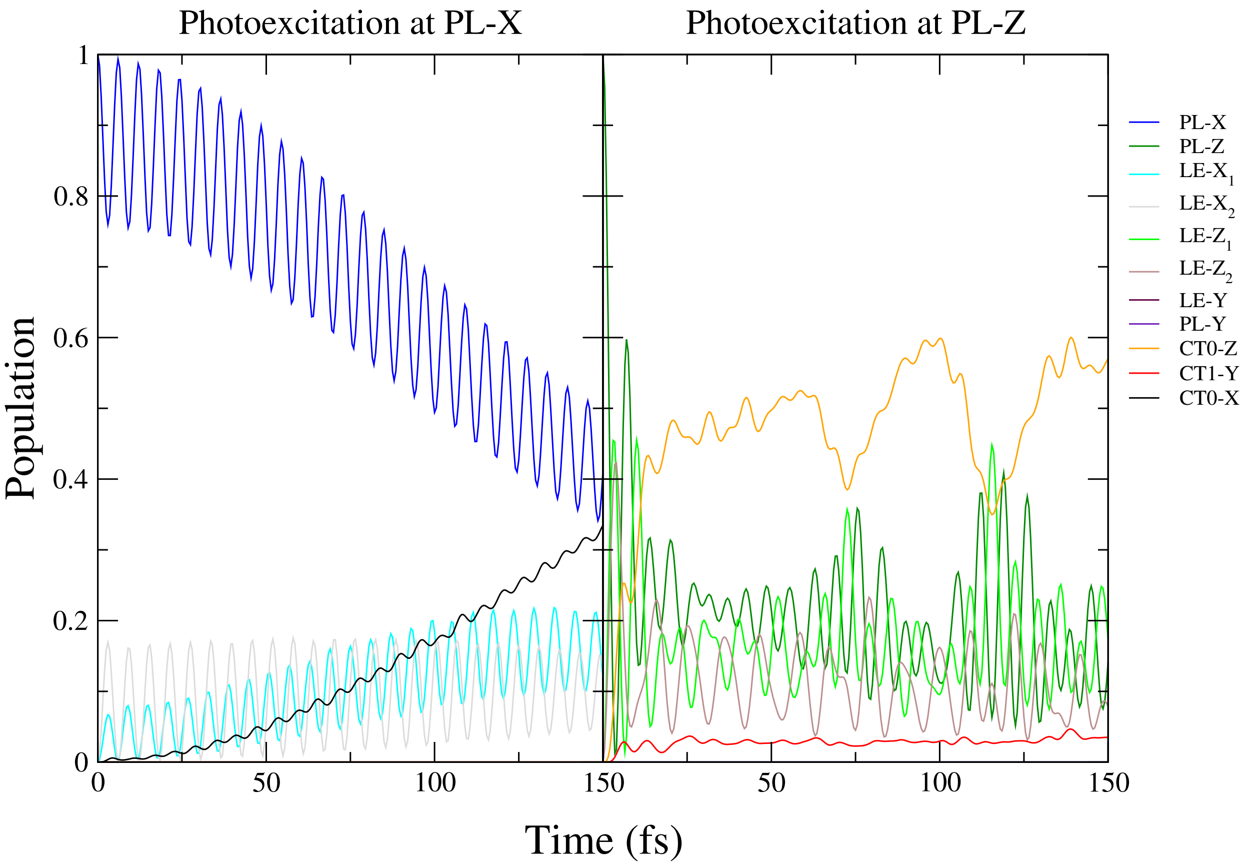

Figure S30: Population dynamics of diabatic states for $[Ag_6PPy]$-45 turning off PL-CT electronic couplings.

8.2 $[Ag_{20}V,SPy]+\vec{E}$ Wavepacket Propagations

In these systems with less symmetry and restrictions, all PL and CT states can interact. However, as shown in Figure S23, the interaction between PL states is negligible. On the contrary, the PL-CT interaction is sizable as shown in Figure S24, therefore, PL and CT are strongly coupled. Furthermore, the PL states are able to interact amongst them through the PL-CT coupling. Finally, the CT-CT constant couplings are not negligible and they are also reinforced by the linear coupling on non-totally symmetric modes for CT0-CT1.

The population dynamics of the diabatic states of $[Ag_{20}VPy]$ (Figures S31-S33) is similar for photoexcitation to any PL states: The maximum population transfer to CT states is found for $E=10^{-4}$ a.u., while it is negligible for more positive E and decays slowly as E becomes smaller. However, the most effective population transfer to any CT state is between PL_1 and $CT0_2$, which achieves values as high as 80-90%, while for other PL states do not become larger than 15%.

Differently, the stronger mixing in $[Ag_{20}SPy]$ allows for the population of CT states on a wide range of E, as shown in Figures S34-S36. The analysis is less straightforward because the population can migrate to other PL states (Figures S31 and S32) or to several CT states that can be populated sequentially. For instance, in Figure S32, most of the population flows first towards $CT0_2$ at larger E, but as E becomes more negative the states $CT0_3$ and $CT1_2$ gain population. Similarly, photoexciting to PL_3 at negative values of E results to the tuning of state $CT1_1$ before detuning $CT0_1$.

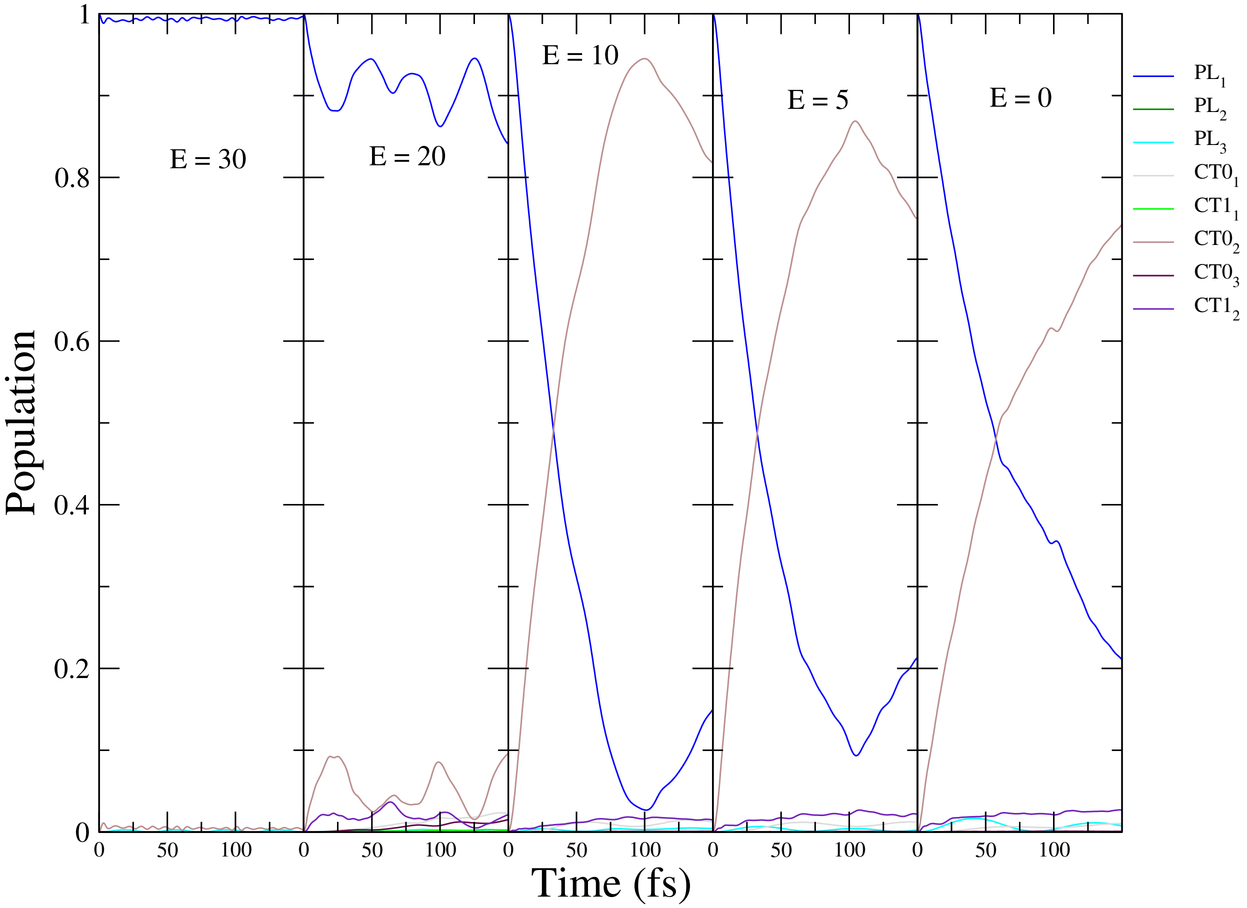

Figure S31: Population dynamics of diabatic states for $[Ag_{20}VPy]+\vec{E}$ systems photoexciting to state PL_1. E expressed in 10^{-4} a.u.

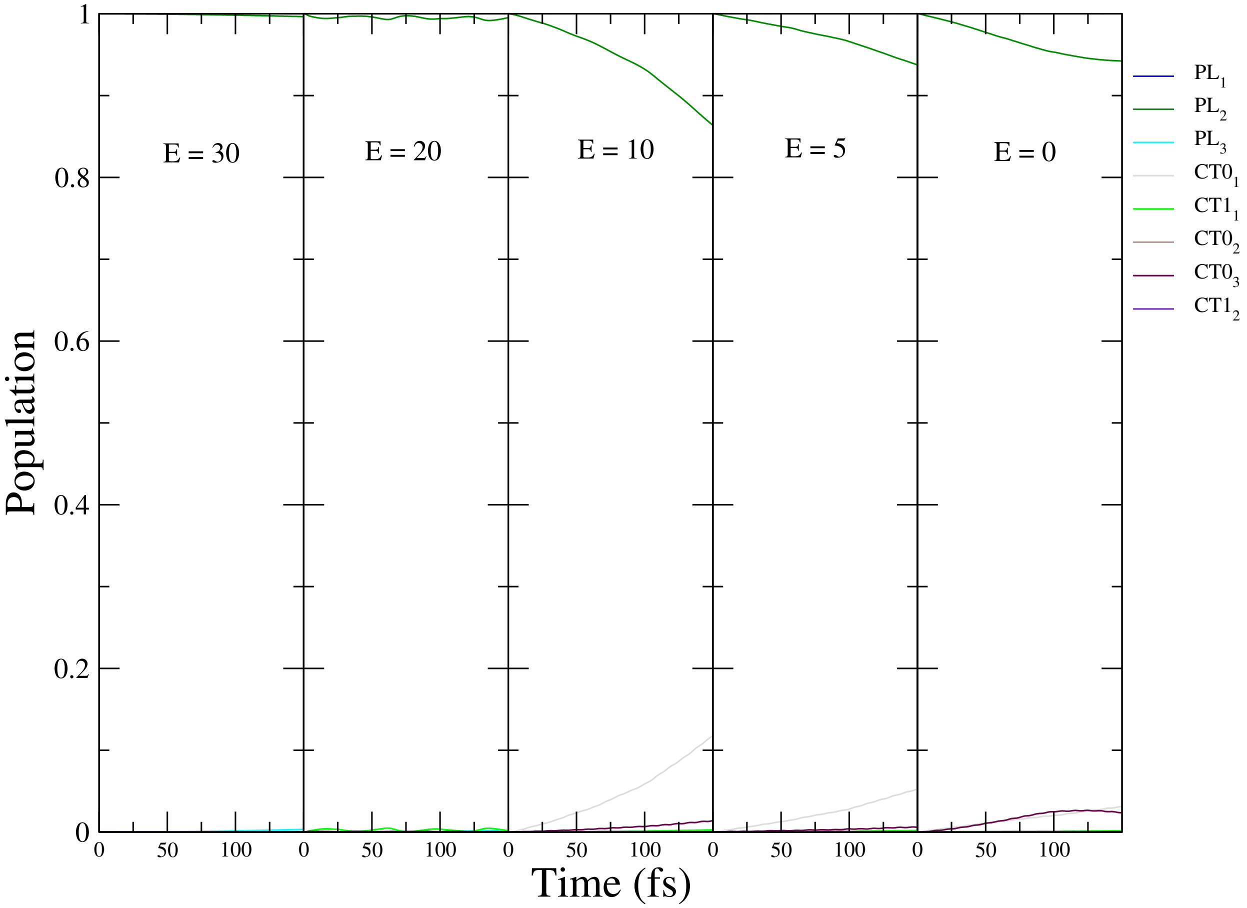

Figure S32: Population dynamics of diabatic states for $[Ag_{20}VPy]+\vec{E}$ systems photoexciting to state PL_2. E expressed in 10^{-4} a.u.

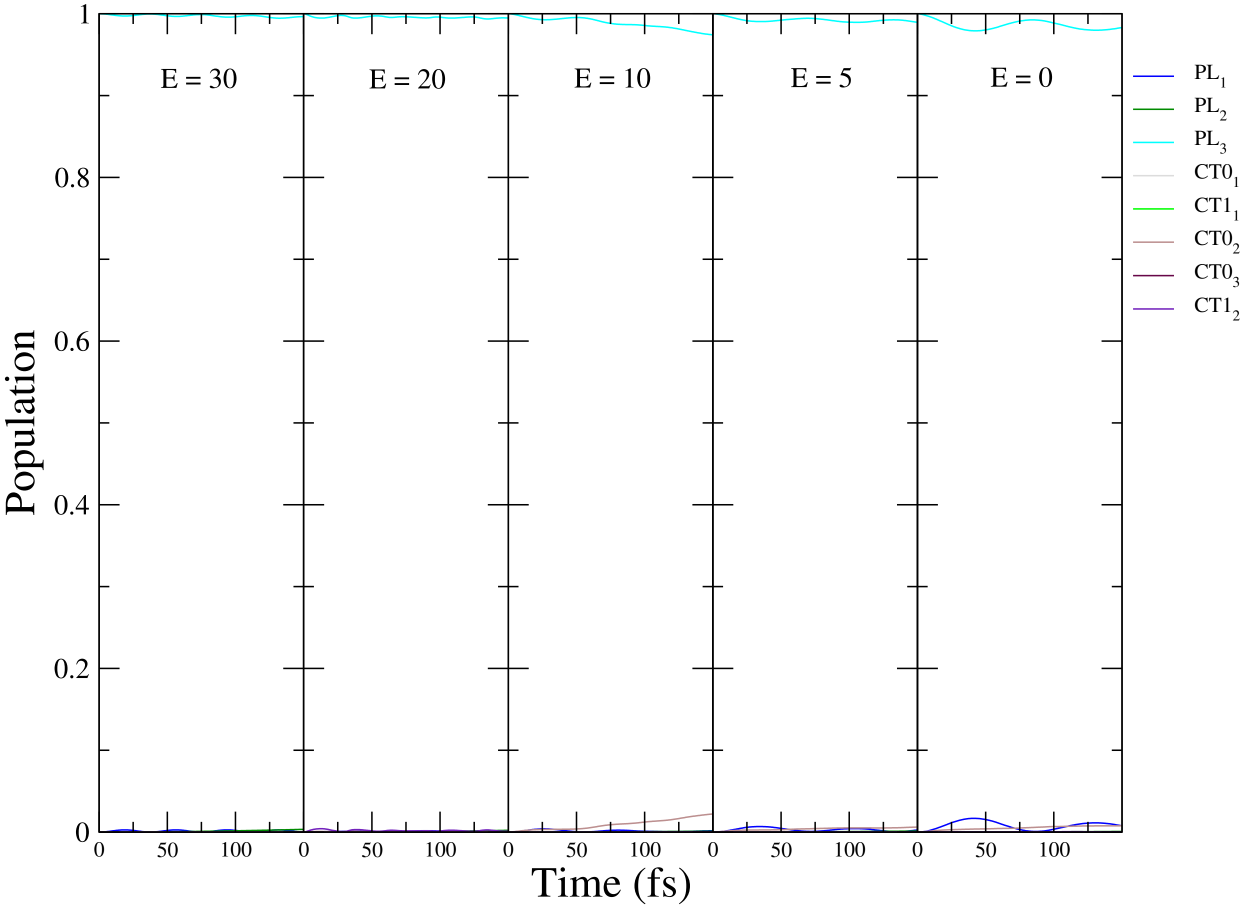

Figure S33: Population dynamics of diabatic states for $[Ag_{20}VPy]+\vec{E}$ systems photoexciting to state PL_3. E expressed in 10^{-4} a.u.

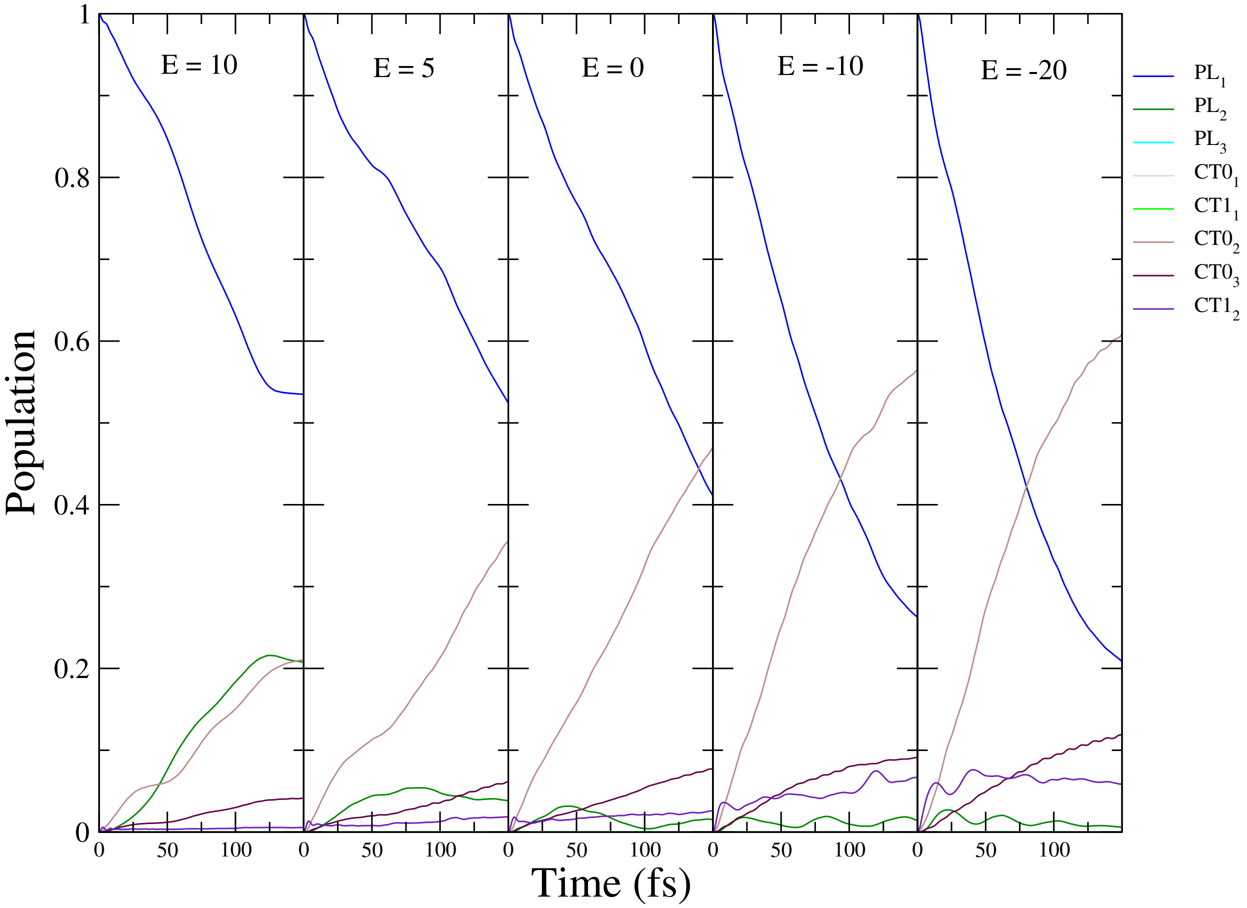

Figure S34: Population dynamics of diabatic states for $[Ag_{20}SPy]+\vec{E}$ systems photoexciting to state PL_1. E expressed in 10^{-4} a.u.

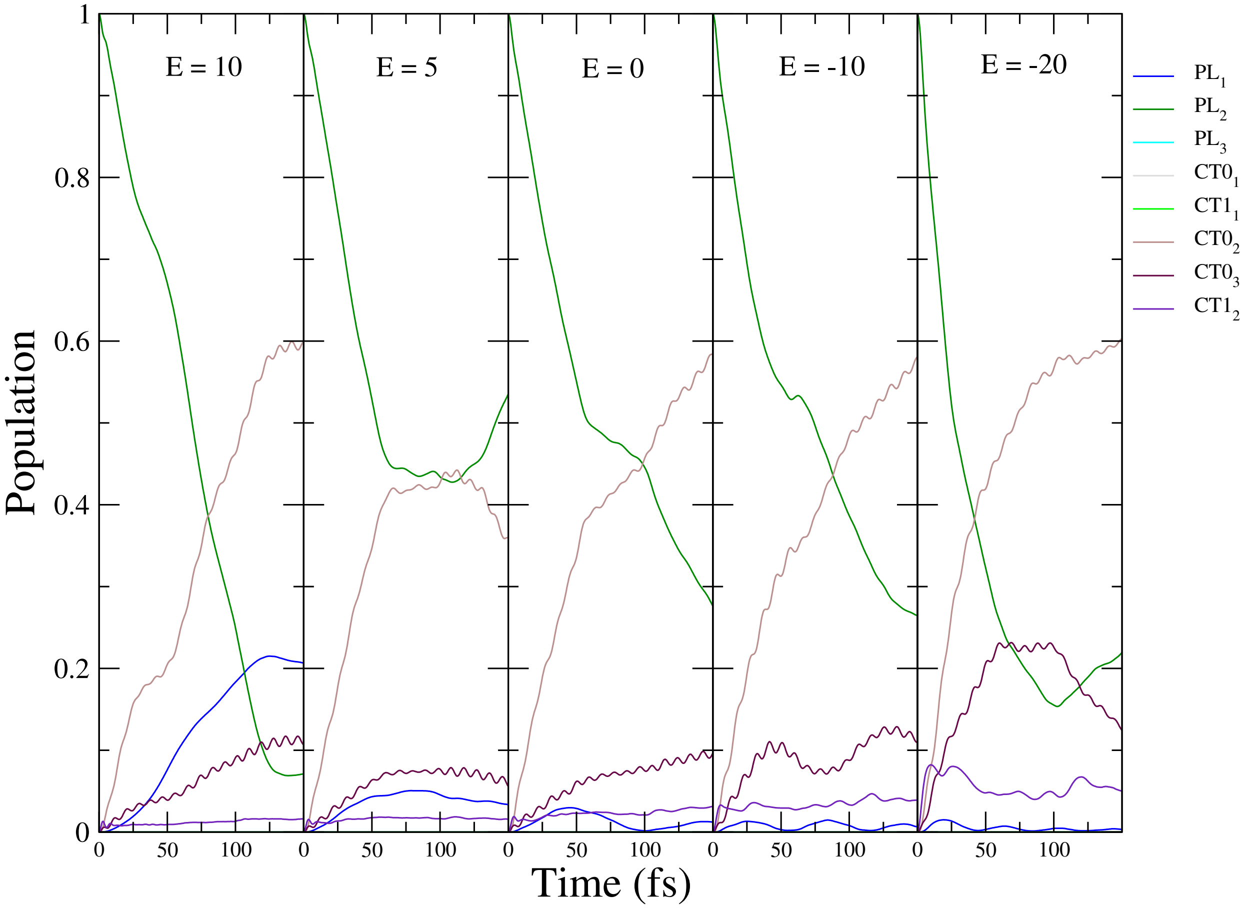

Figure S35: Population dynamics of diabatic states for $[Ag_{20}SPy]+\vec{E}$ systems photoexciting to state PL_2. E expressed in 10^{-4} a.u.

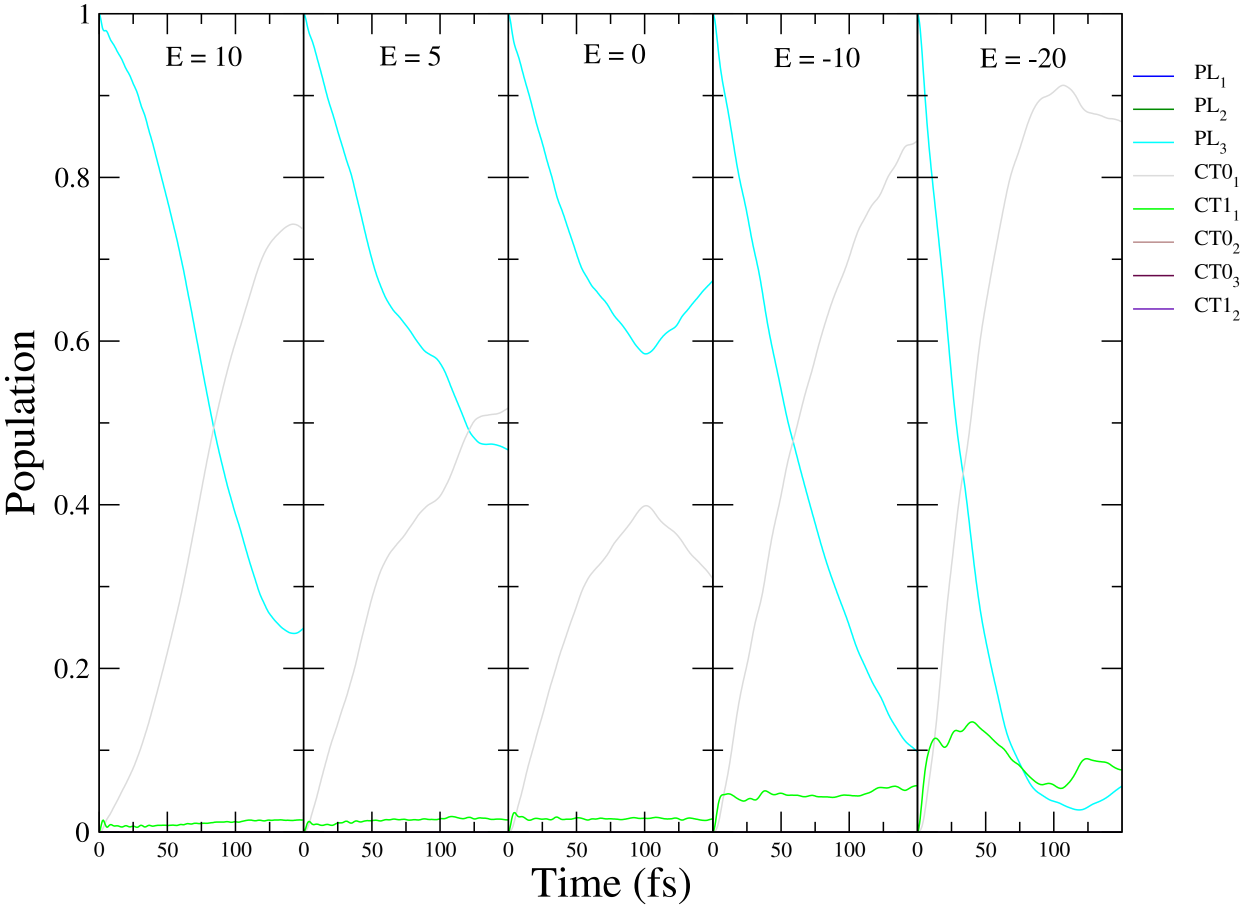

Figure S36: Population dynamics of diabatic states for $[Ag_{20}SPy]+\vec{E}$ systems photoexciting to state PL_3. E expressed in 10^{-4} a.u.

9 Additional Spectra and Excitation Profiles

9.1 Additional Excitation Profiles

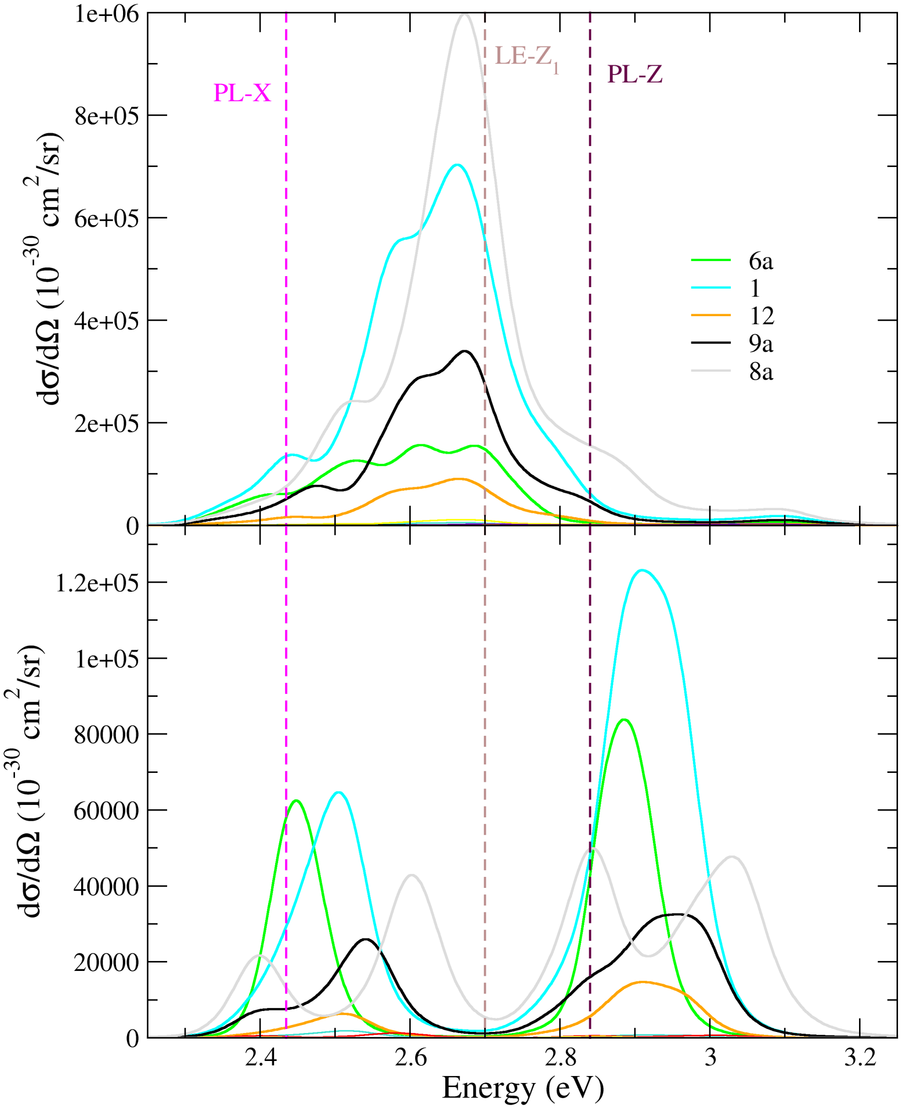

Figure S37: Excitation profile of $[Ag_6PPy]$-45 system including all LVC parameters (top) or turning off the coupling with the LE-Z_1 and LE-Z_2 states (bottom).

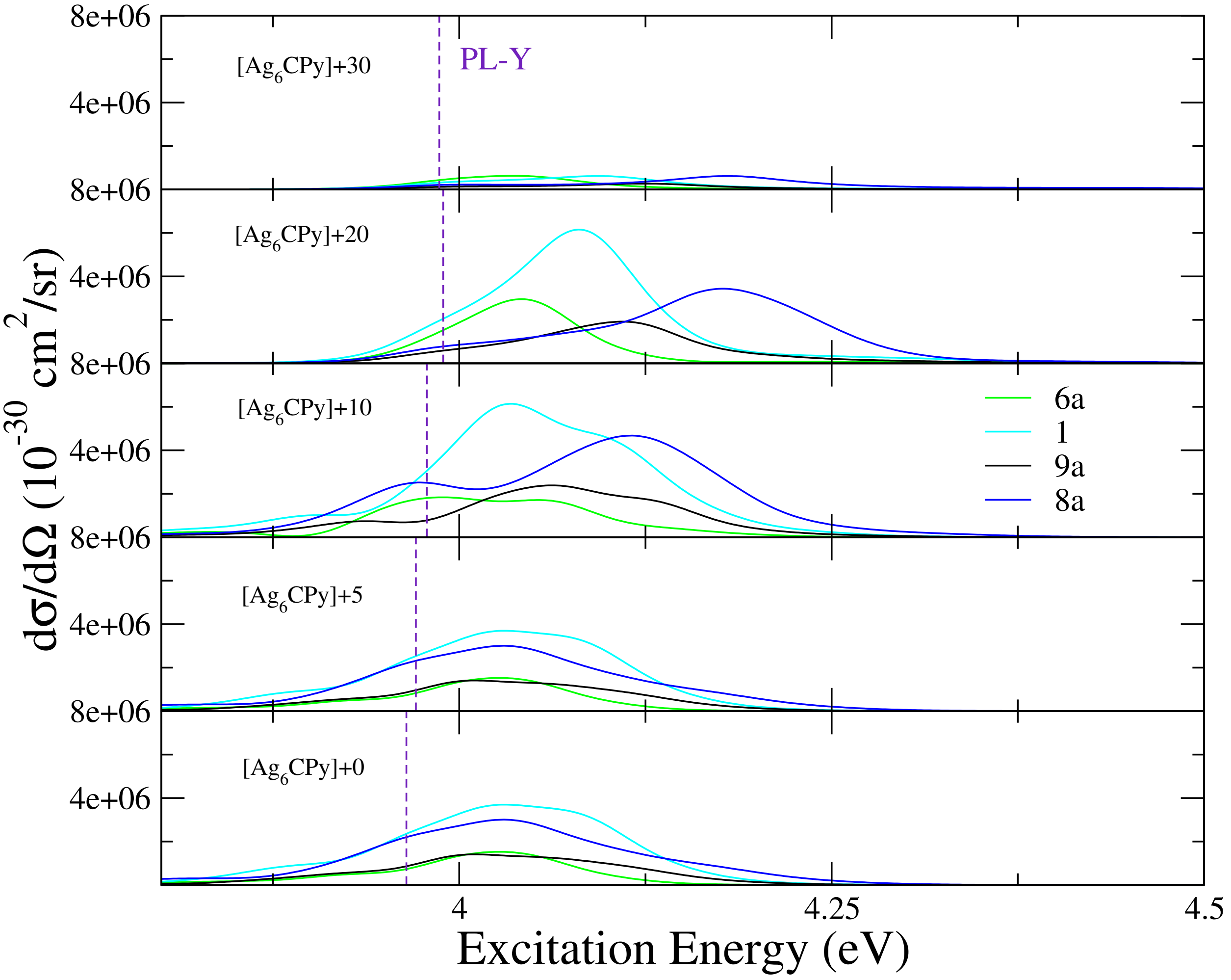

Figure S38: Excitation profiles of $[Ag_6CPy]+\vec{E}$ systems for the most relevant Py normal modes in SERS.

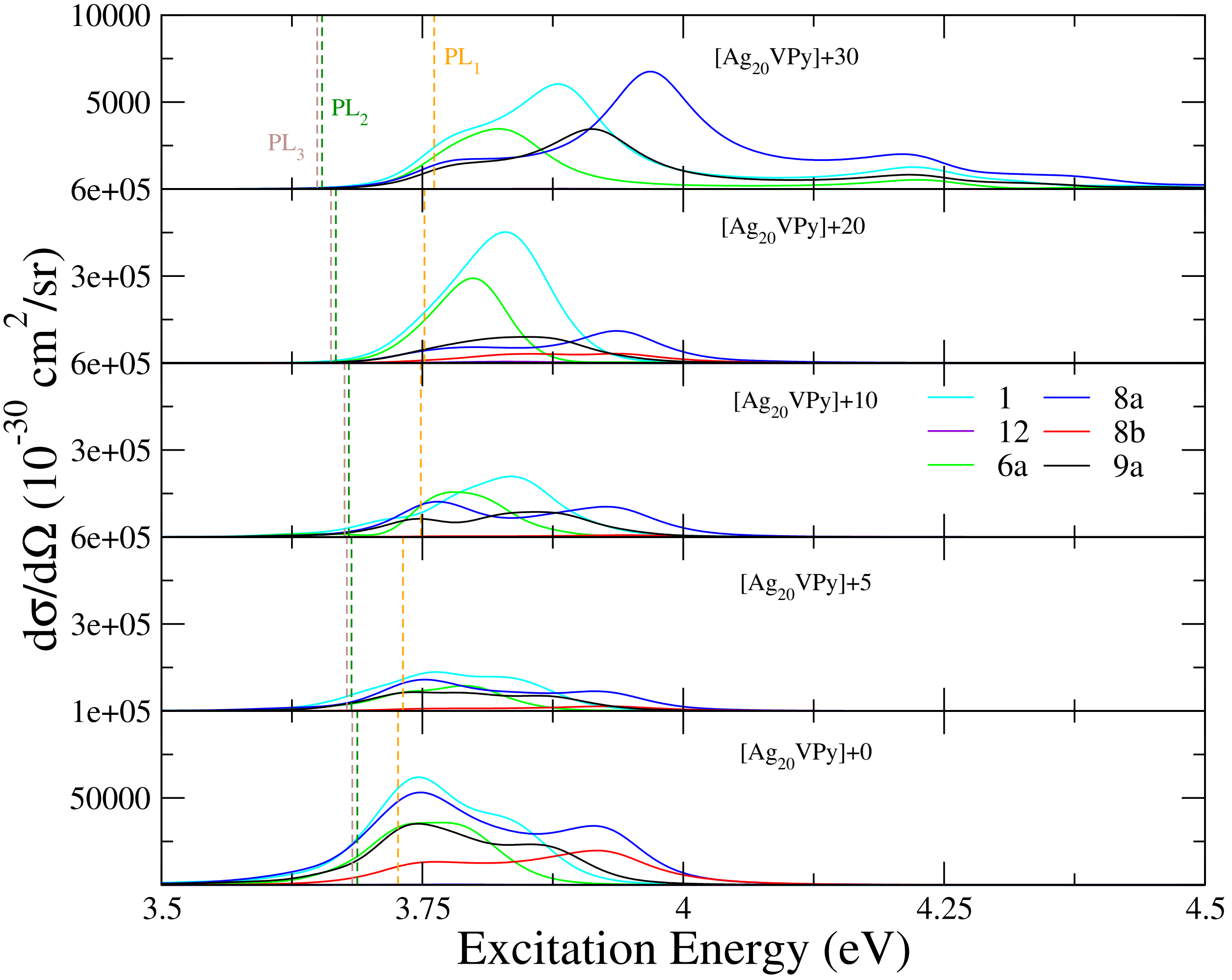

Figure S39: Excitation profiles of $[Ag_{20}VPy]+\vec{E}$ systems for the most relevant Py normal modes in SERS.

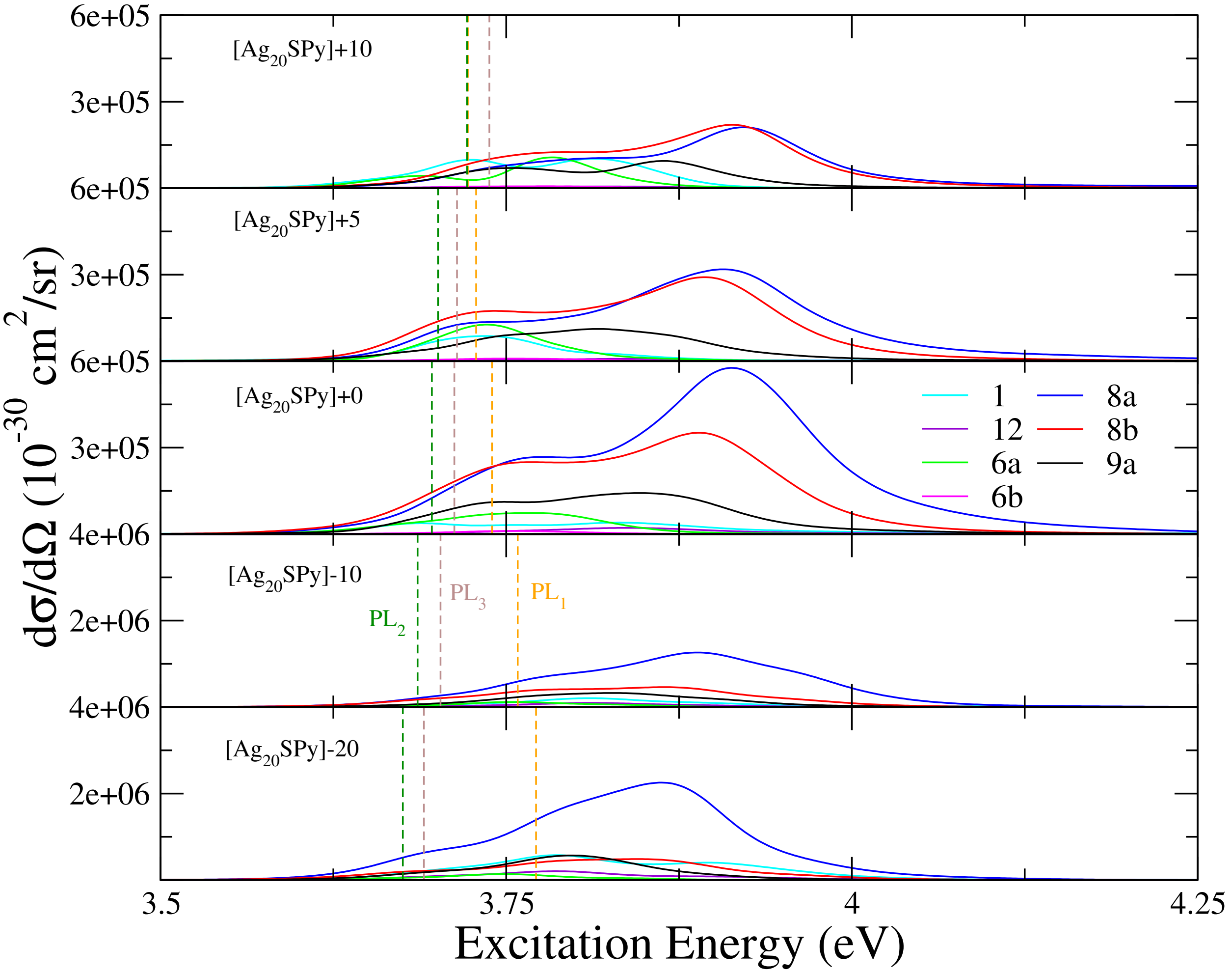

Figure S40: Excitation profiles for $[Ag_{20}SPy]+\vec{E}$ systems for the most relevant Py normal modes in SERS.

9.2 Transition Polarizability Components

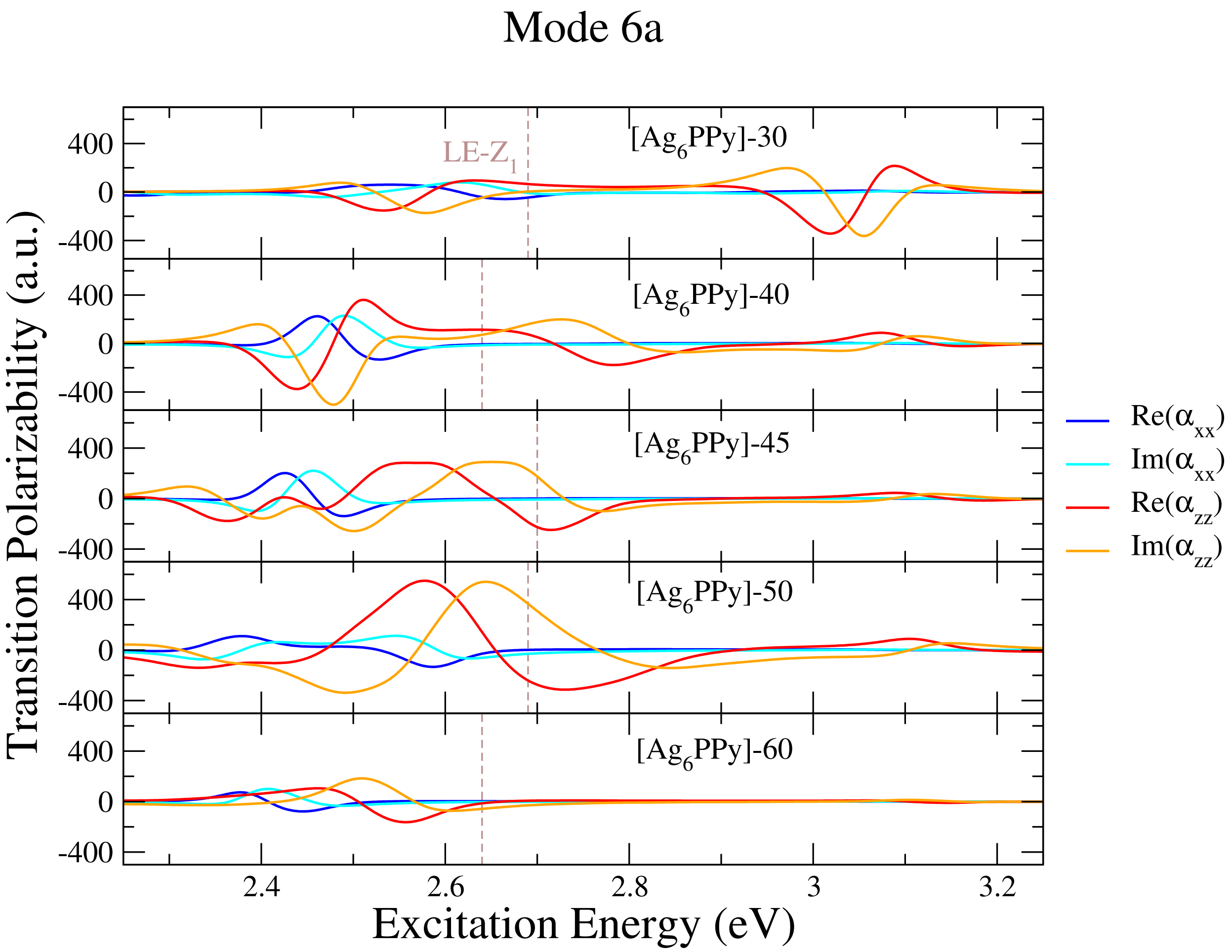

Figure S41: Mode 6a non-zero transition polarizability tensor components for $[Ag_6PPy]+\vec{E}$ systems.

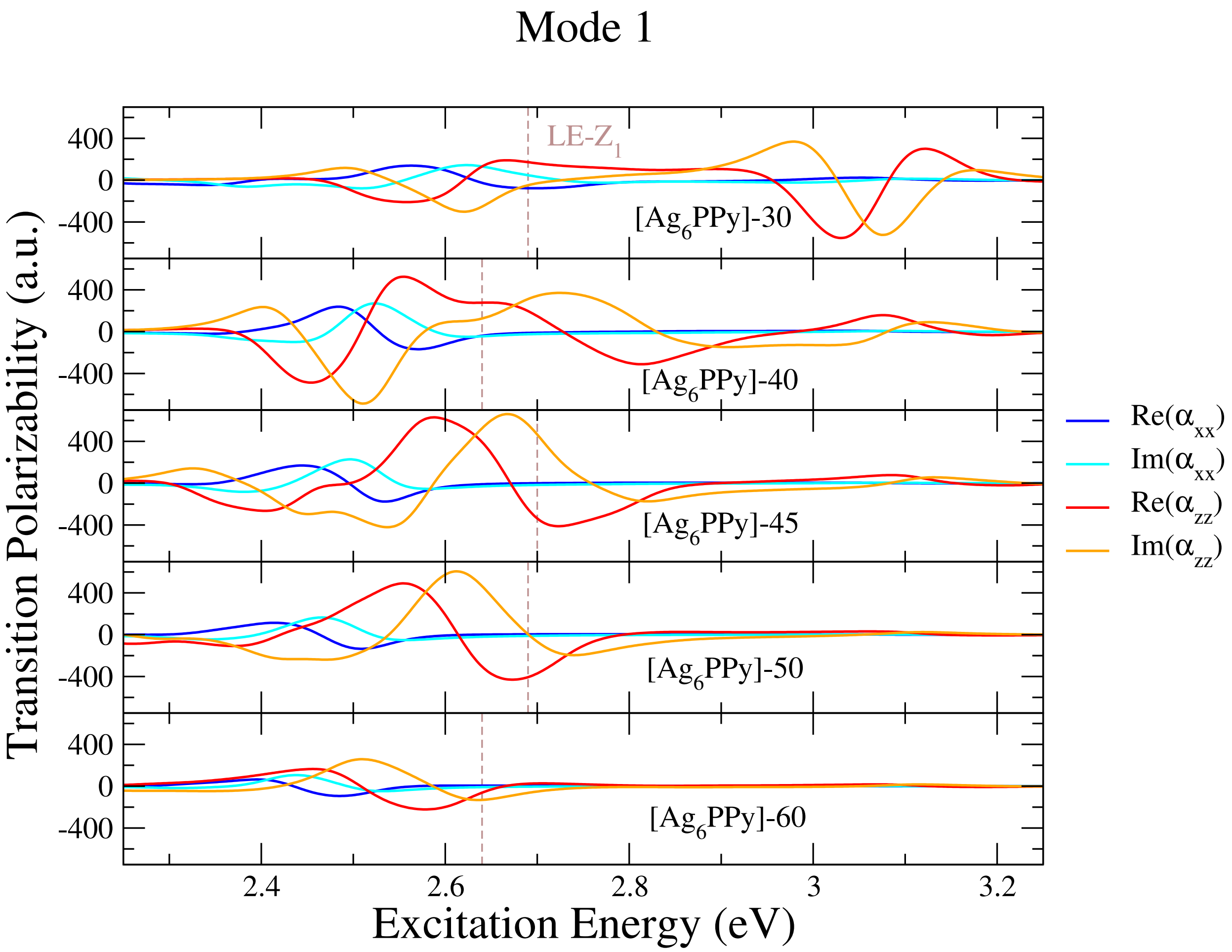

Figure S42: Mode 1 non-zero transition polarizability tensor components for $[Ag_6PPy]+\vec{E}$ systems.

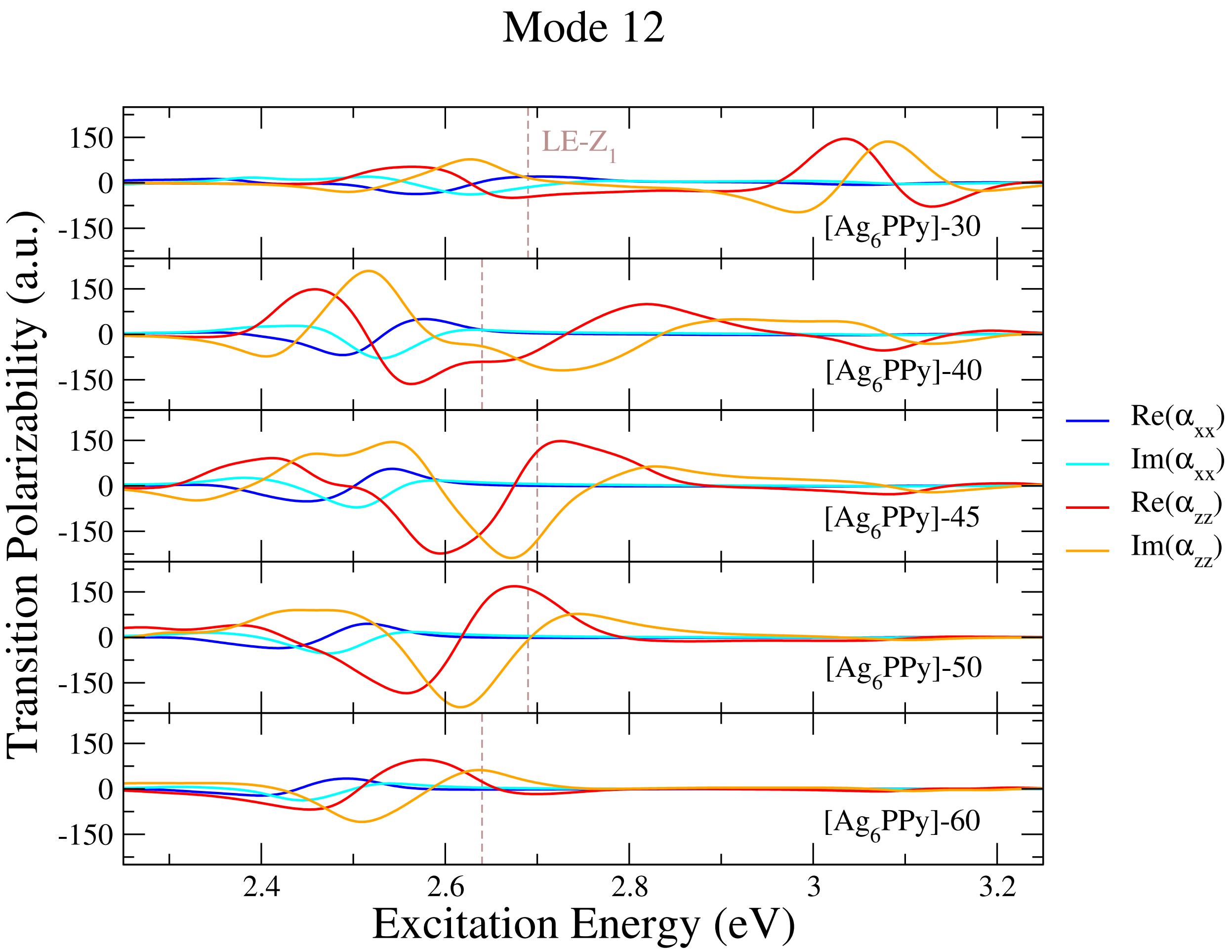

Figure S43: Mode 12 non-zero transition polarizability tensor components for $[Ag_6PPy]+\vec{E}$ systems.

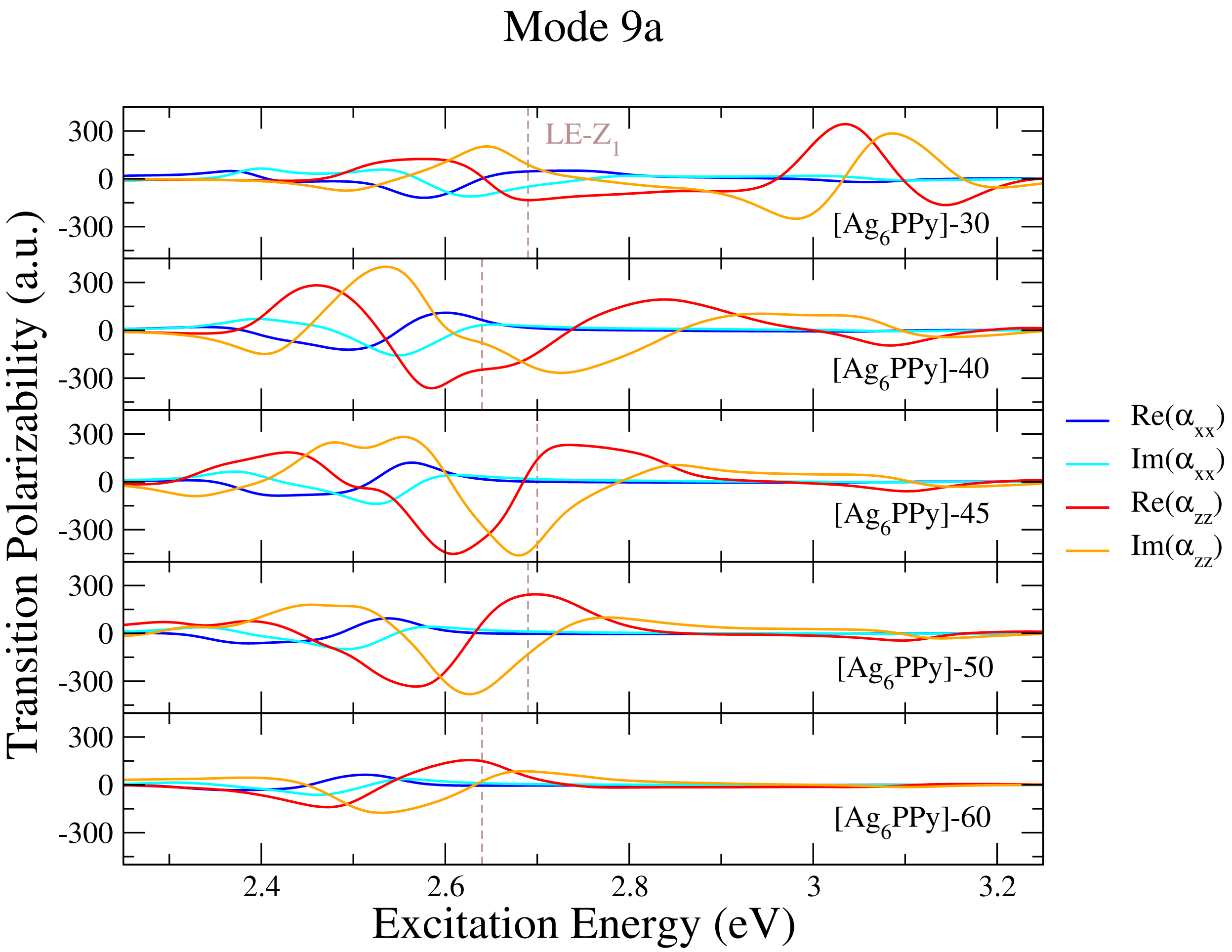

Figure S44: Mode 9a non-zero transition polarizability tensor components for [Ag_6PPy]+$\vec{E}$ systems.

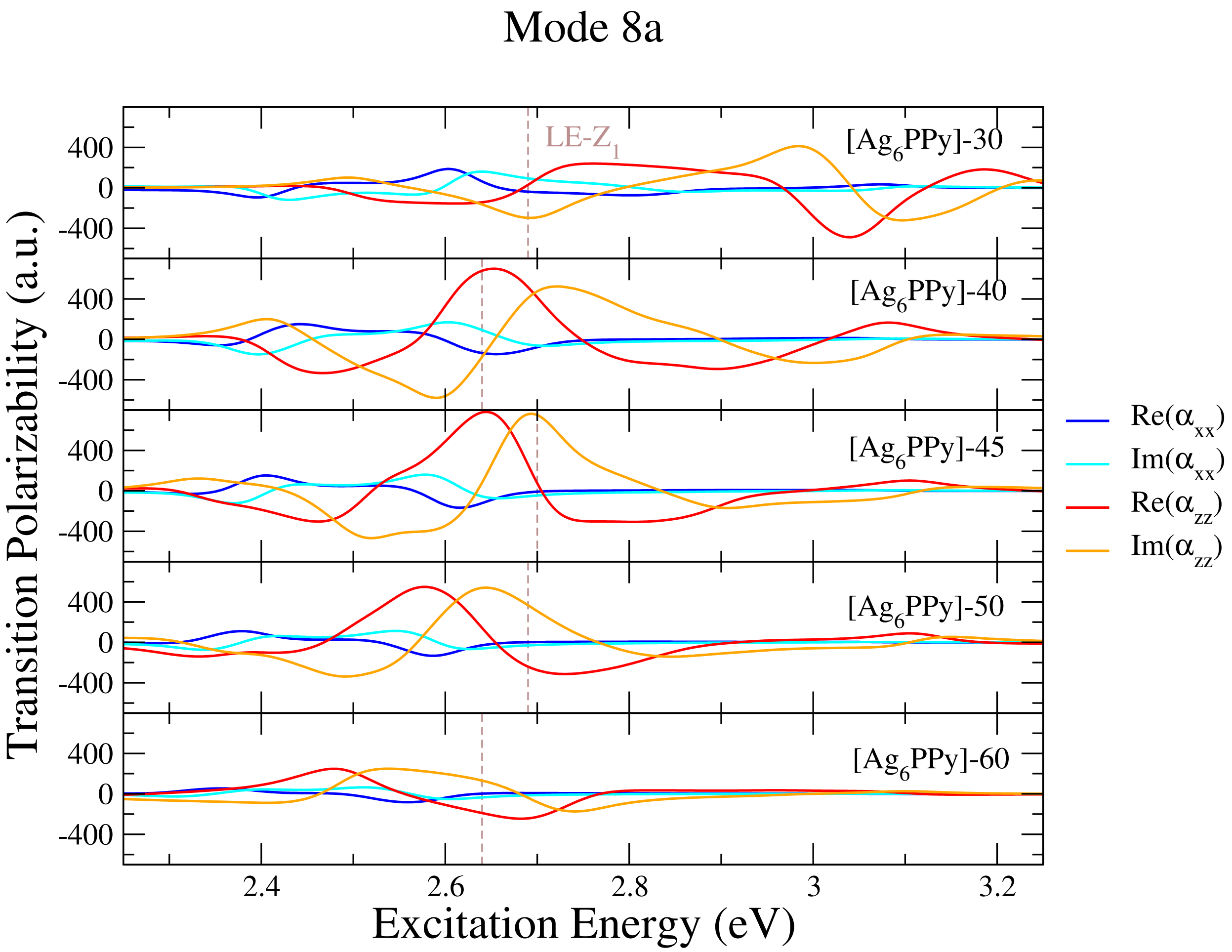

Figure S45: Mode 8a non-zero transition polarizability tensor components for [Ag_6PPy]+$\vec{E}$ systems.

9.3 Experimental Spectra

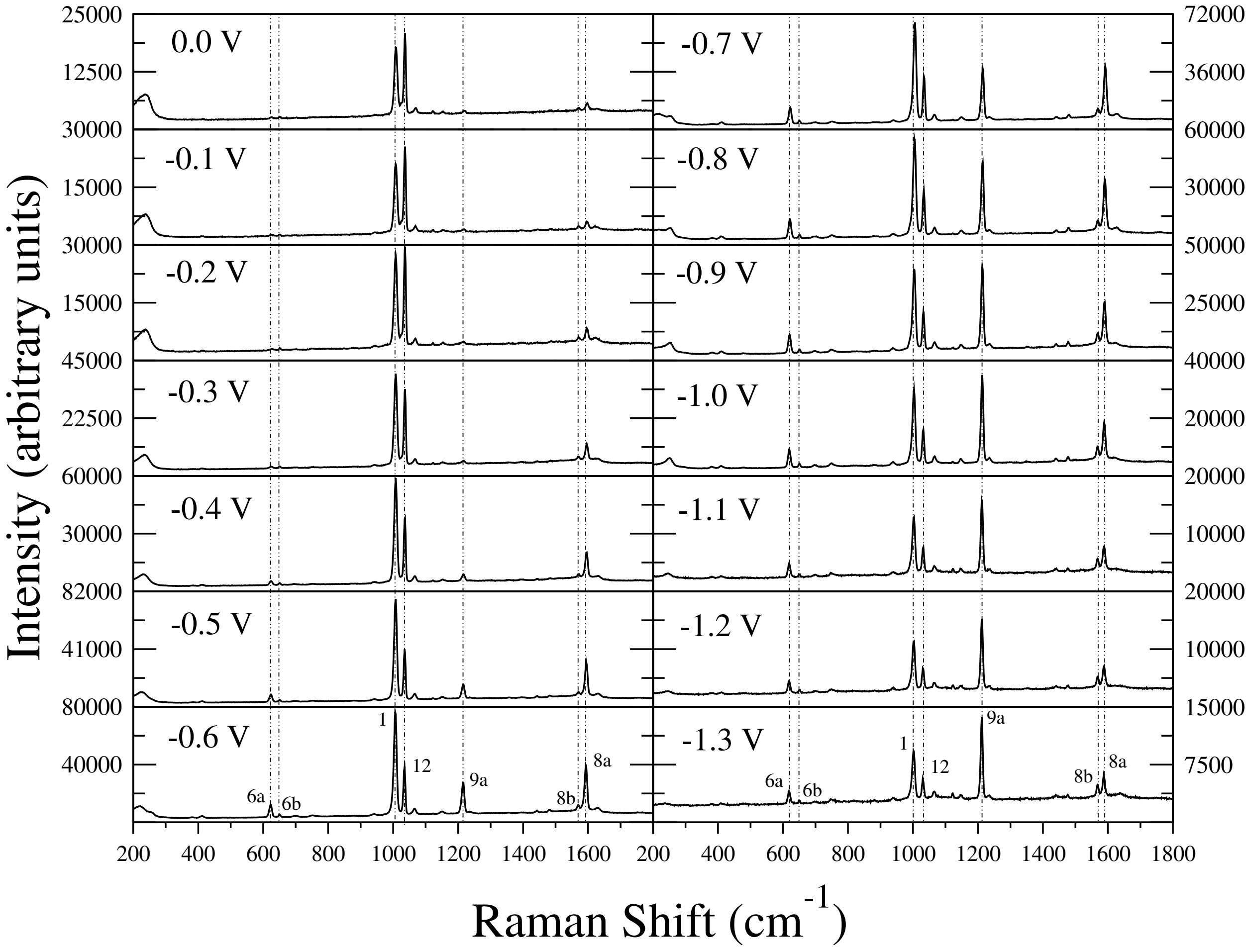

Figure S46: Experimental EC-SERS spectra of Py in water on a roughened silver electrode with an excitation line of 514 nm. Data from Ref. S4.

9.4 [Ag_6P,CPy]+$\vec{E}$ Spectra

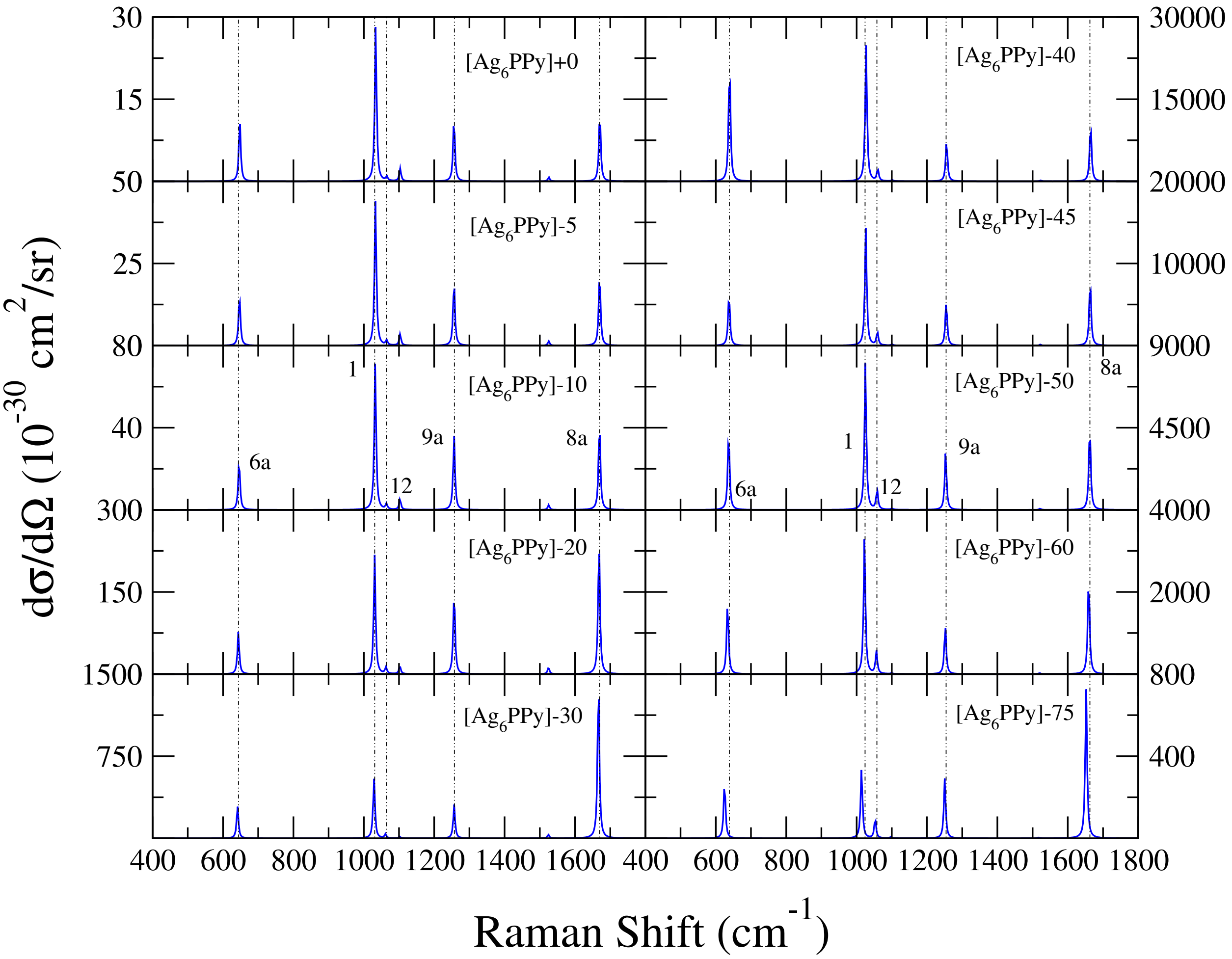

Figure S47: Resonance Raman Spectra for [Ag_6PPy]+$\vec{E}$ systems. The value of $\hbar\omega_I$ was taken identical to the excitation energy of state PL-X for each system. All stick transitions were convoluted with a Lorentzian of half-width at half-maximum of 3 cm^{-1}

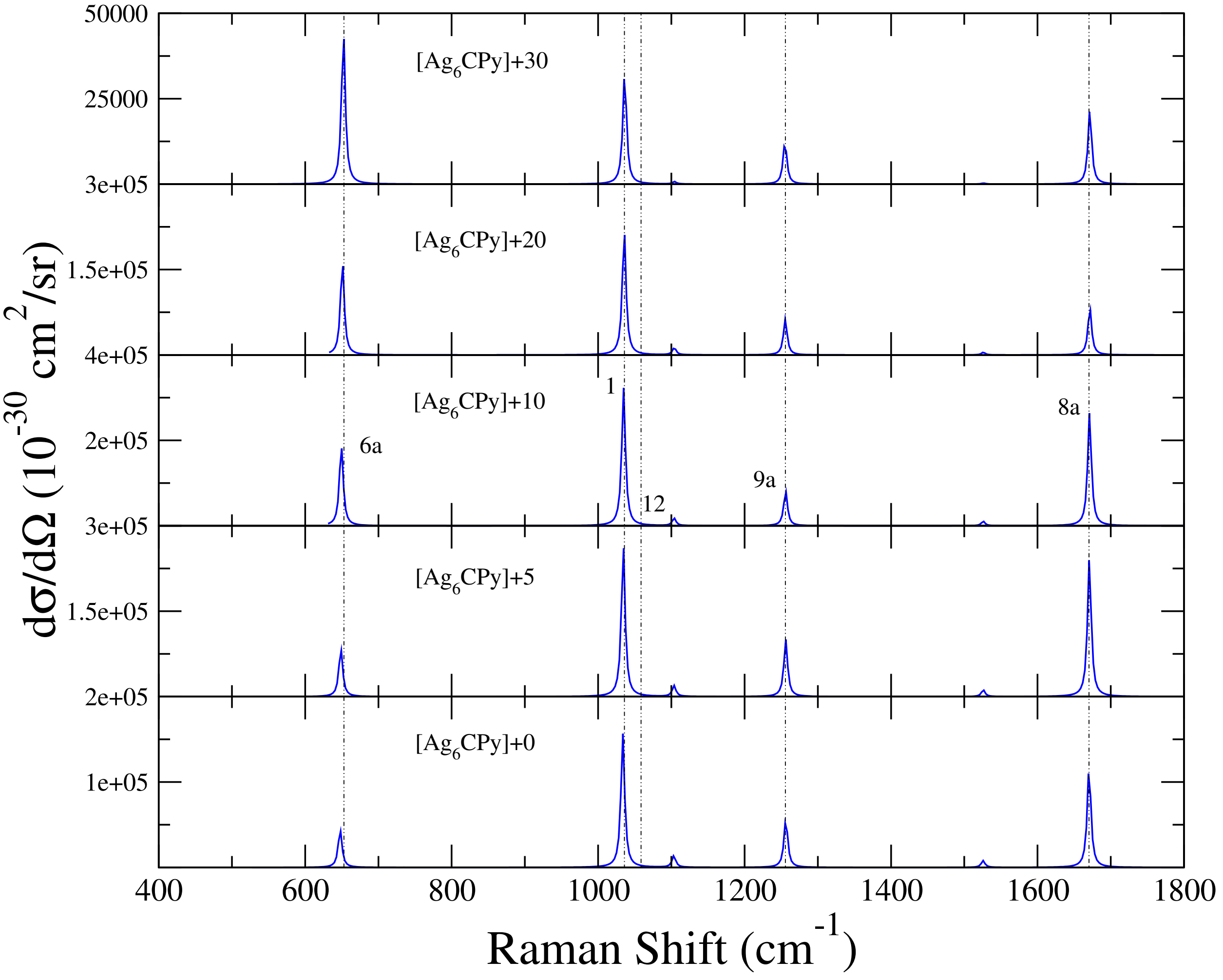

Figure S48: Resonance Raman Spectra for [Ag_6CPy]+$\vec{E}$ systems. The model includes 1 PL and 1 CT states. The value of $\hbar\omega_I$ was taken identical to the excitation energy of state PL-Y of each system. All stick transitions were convoluted with a Lorentzian of half-width at half-maximum of 3 cm^{-1}

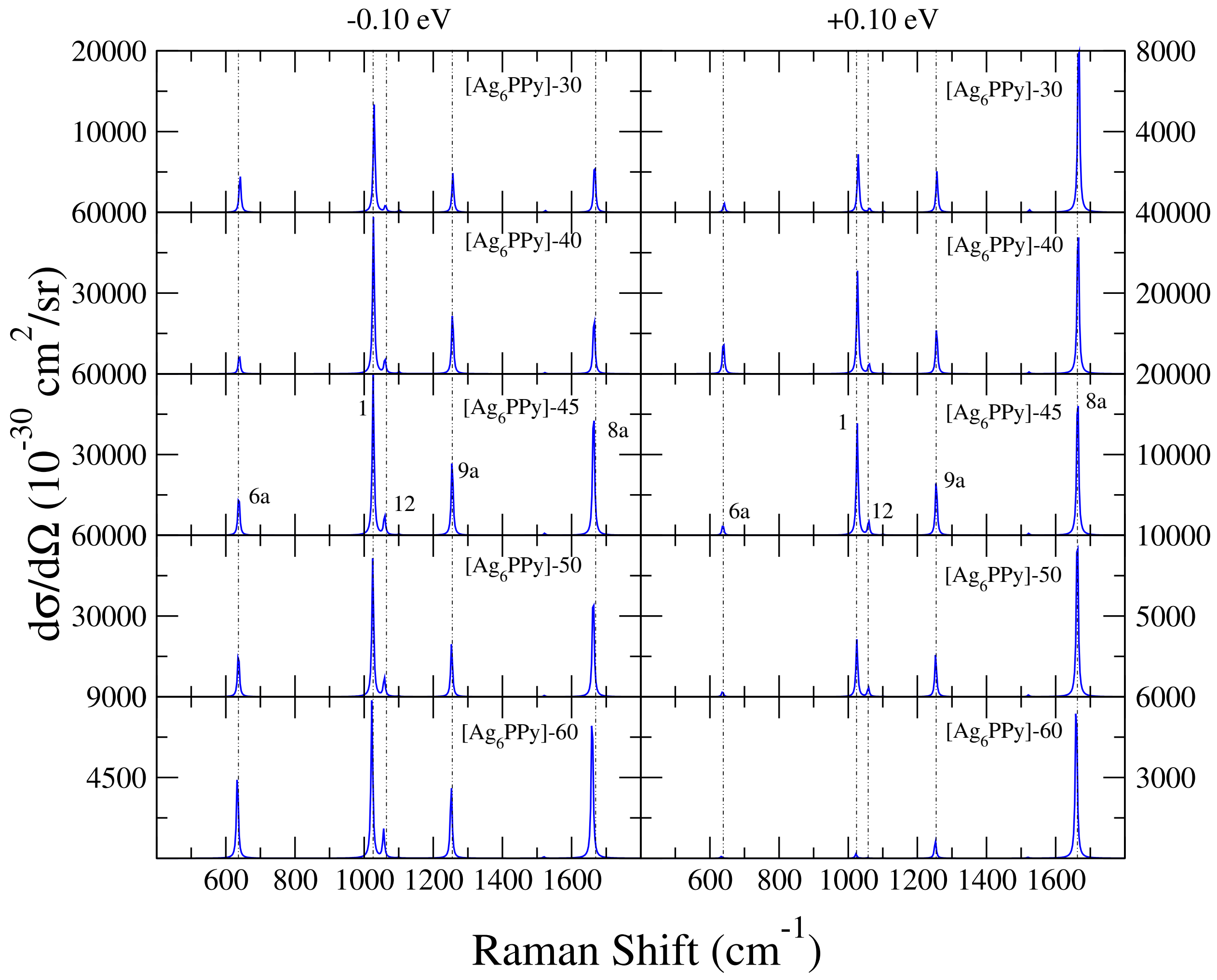

Figure S49: Comparison of Resonance Raman Spectra with excitation energy set at E(LE-Z_1)±0.10 eV for [Ag_6PPy]+$\vec{E}$ systems. All stick transitions were convoluted with a Lorentzian of half-width at half-maximum of 3 cm^{-1}.

9.5 [Ag_{20}V,SPy]+$\vec{E}$ Spectra

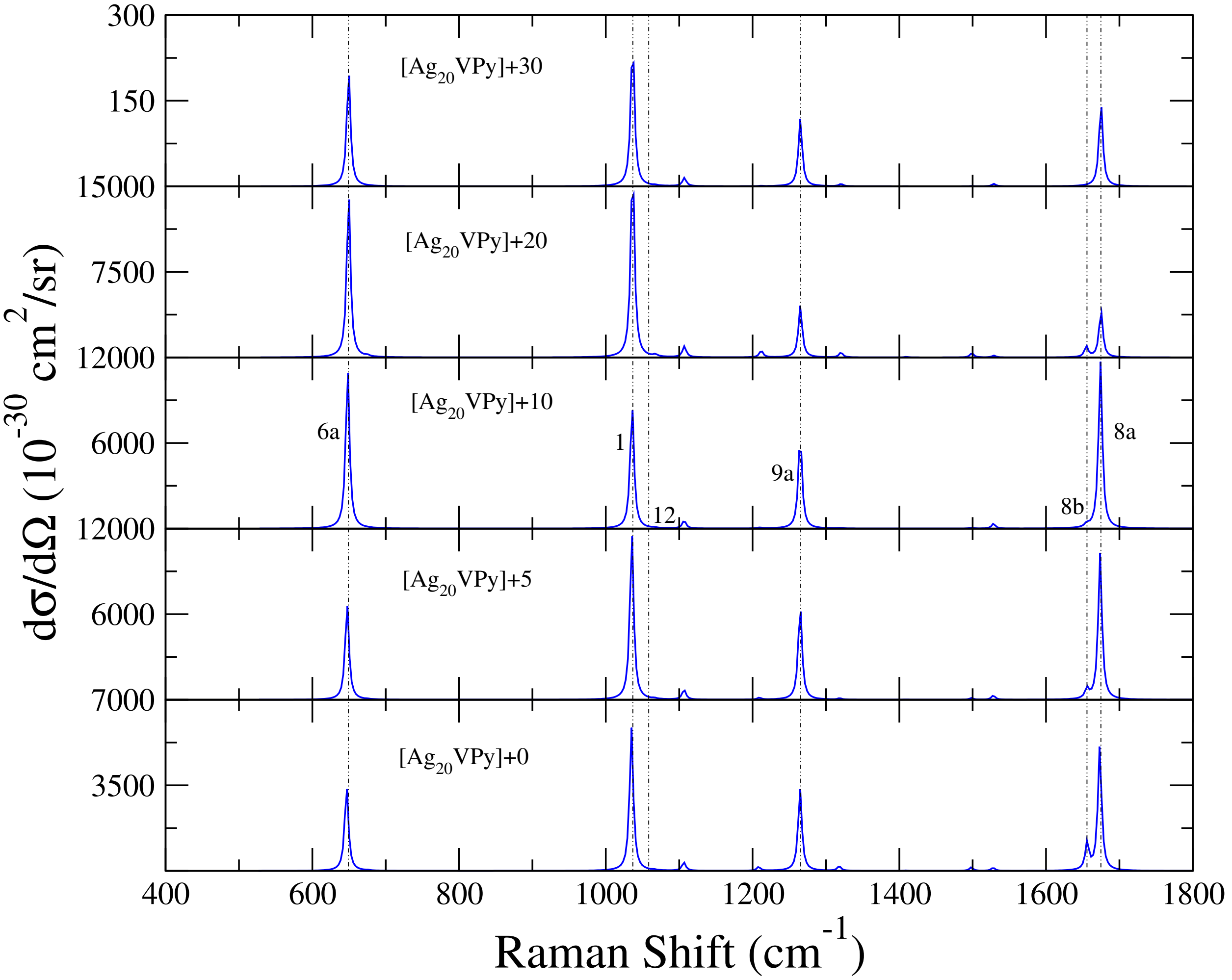

Figure S50: Resonance Raman Spectra for [Ag_{20}VPy]+$\vec{E}$ systems. The model includes 3 PL and 5 CT states. The value of $\hbar\omega_I$ was taken identical to the excitation energy of state PL_1 of each system. All stick transitions were convoluted with a Lorentzian of half-width at half-maximum of 3 cm^{-1}.

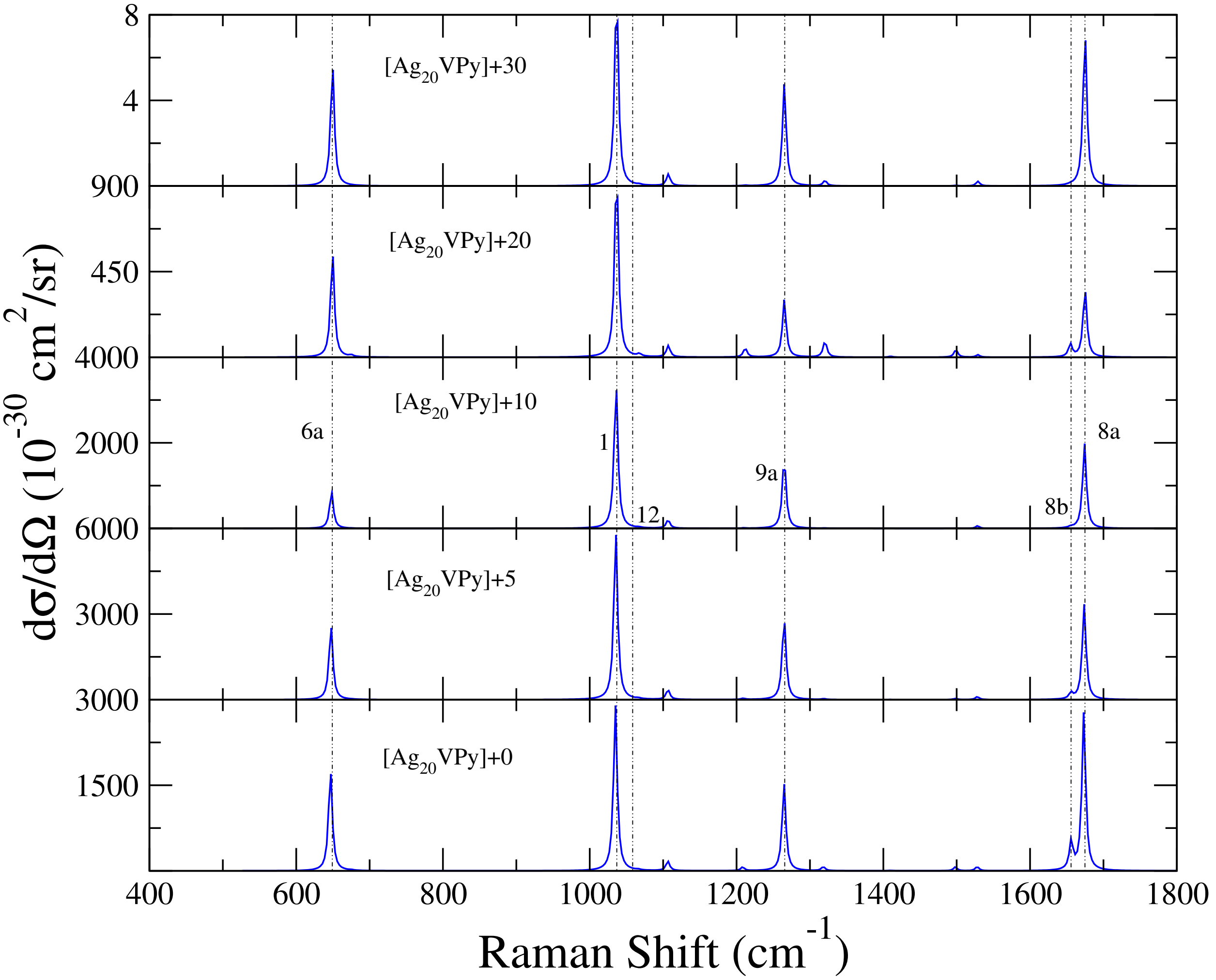

Figure S51: Resonance Raman Spectra for $[Ag_{20}VPy]+\vec{E}$ systems. The model includes 3 PL and 5 CT states. The value of $\hbar\omega_I$ was taken identical to the excitation energy of state PL_2 of each system. All stick transitions were convoluted with a Lorentzian of half-width at half-maximum of 3 cm^{-1}.

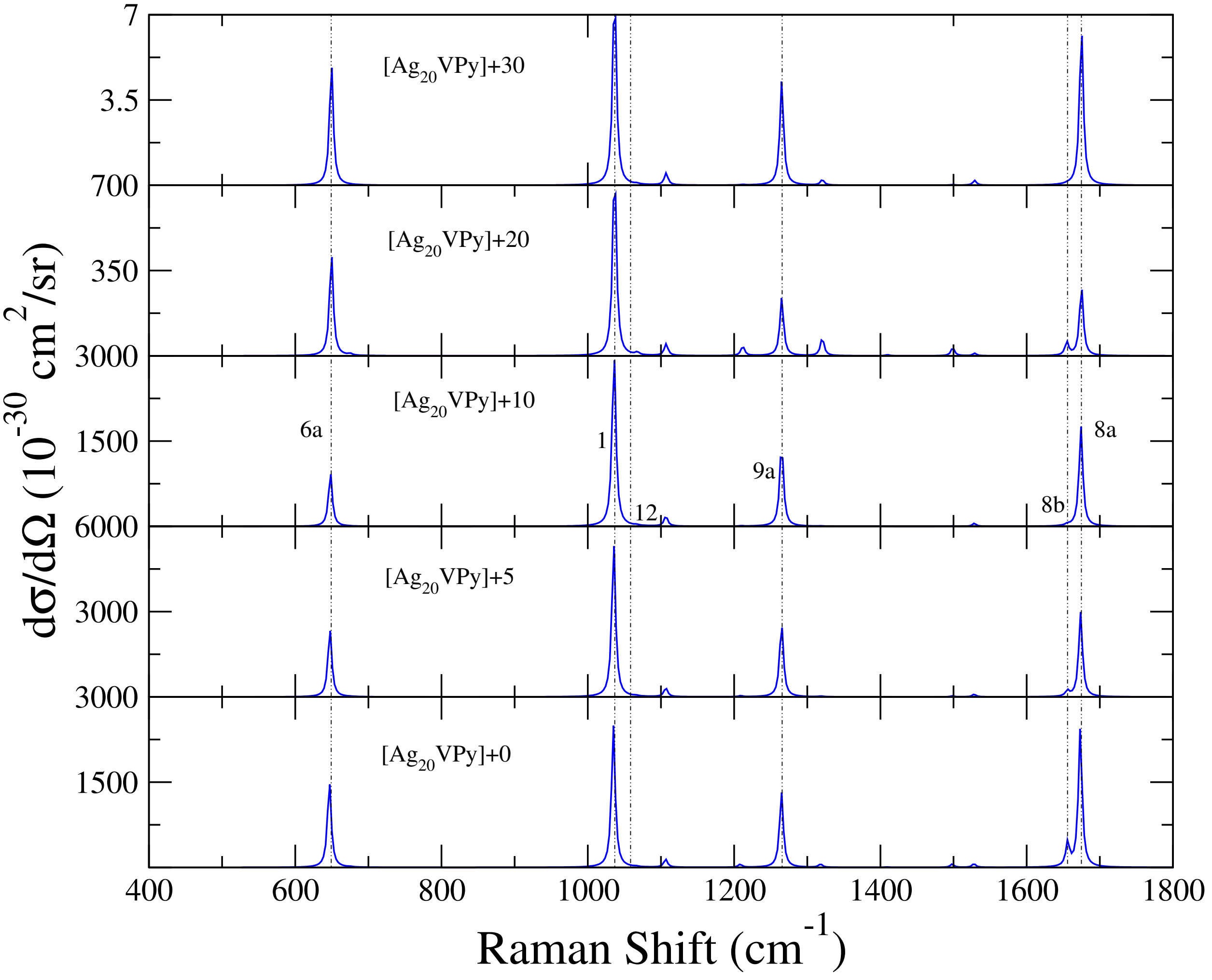

Figure S52: Resonance Raman Spectra for $[Ag_{20}VPy]+\vec{E}$ systems. The model includes 3 PL and 5 CT states. The value of $\hbar\omega_I$ was taken identical to the excitation energy of state PL_3 of each system. All stick transitions were convoluted with a Lorentzian of half-width at half-maximum of 3 cm^{-1}.

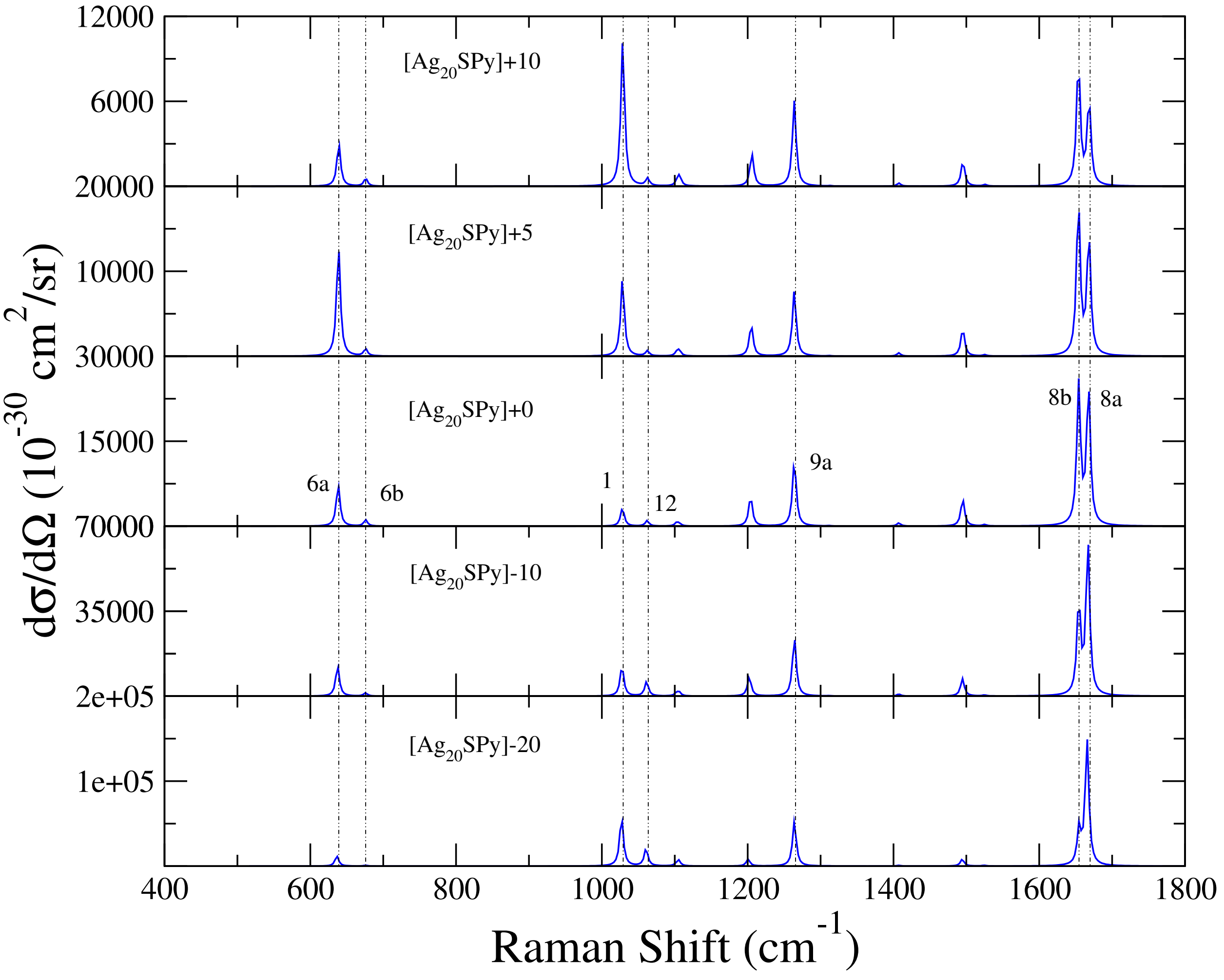

Figure S53: Resonance Raman Spectra for $[Ag_{20}SPy]+\vec{E}$ systems. The model includes 3 PL and 5 CT states. The value of $\hbar\omega_I$ was taken identical to the excitation energy of state PL_1 of each system. All stick transitions were convoluted with a Lorentzian of half-width at half-maximum of 3 cm^{-1}.

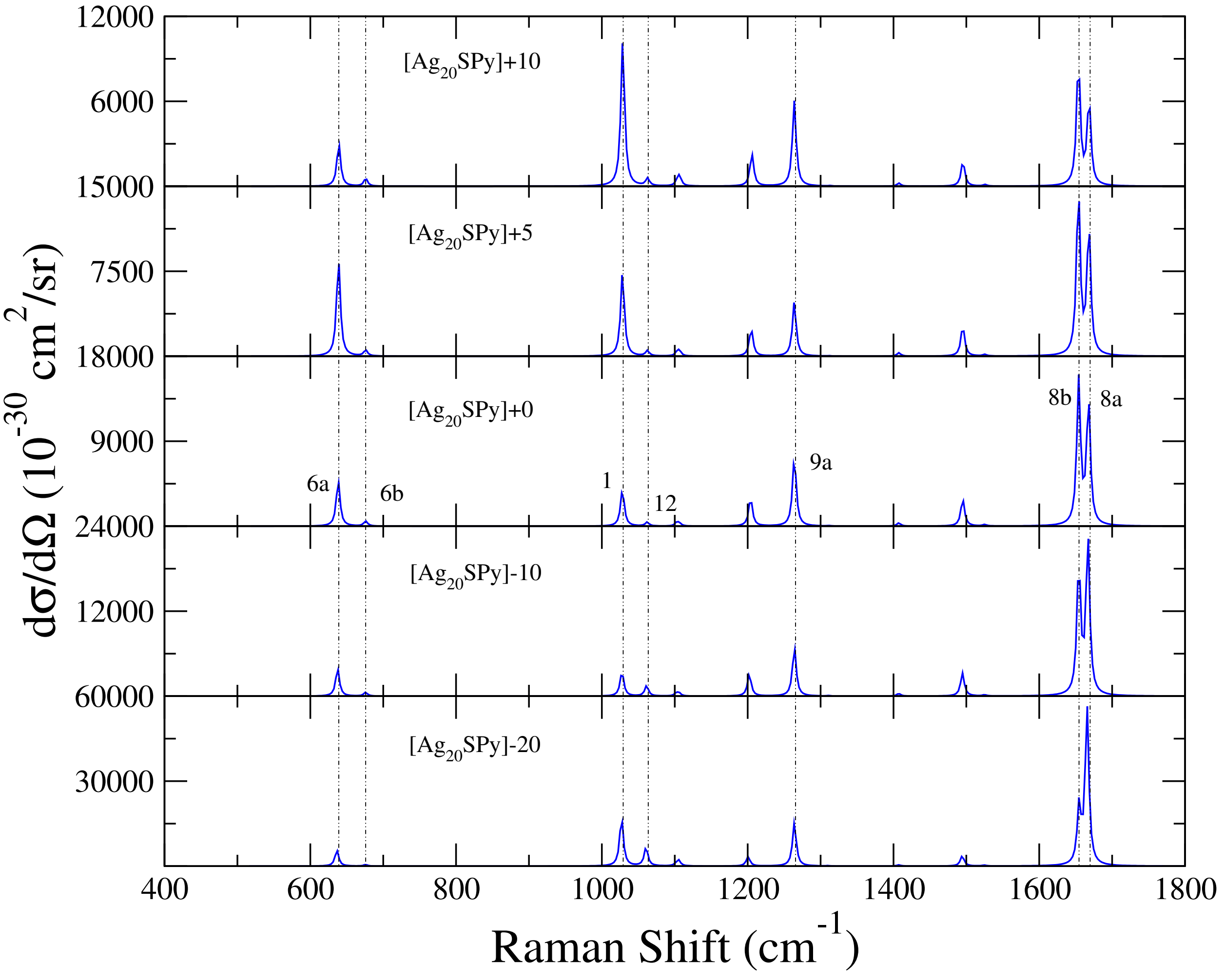

Figure S54: Resonance Raman Spectra for [Ag$_{20}$SPy]+$\vec{E}$ systems. The model includes 3 PL and 5 CT states. The value of $\hbar\omega_I$ was taken identical to the excitation energy of state PL$_2$ of each system. All stick transitions were convoluted with a Lorentzian of half-width at half-maximum of 3 cm^{-1}.

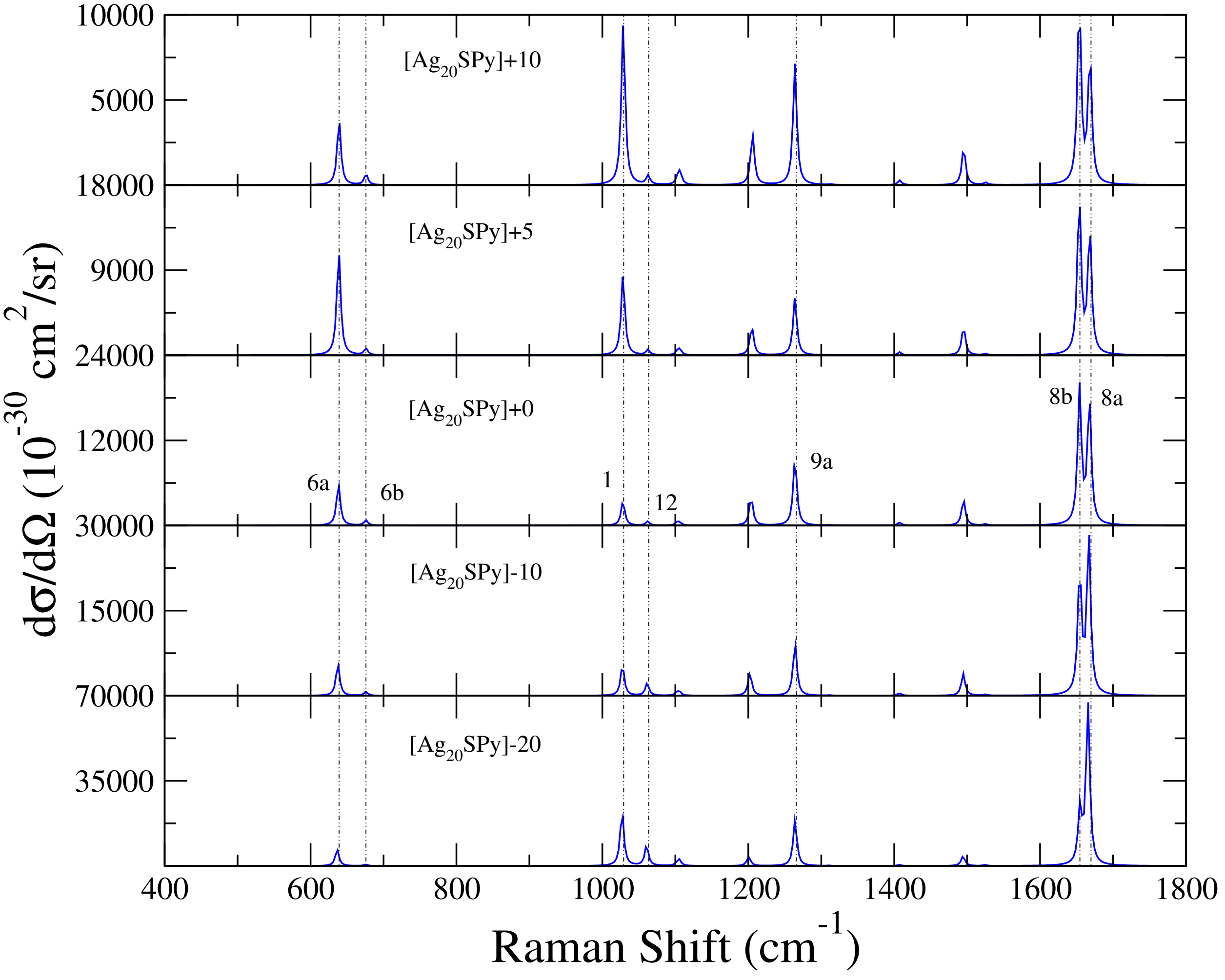

Figure S55: Resonance Raman Spectra for $[Ag_{20}SPy]+\vec{E}$ systems. The model includes 3 PL and 5 CT states. The value of $\hbar\omega_I$ was taken identical to the excitation energy of state PL_3 of each system. All stick transitions were convoluted with a Lorentzian of half-width at half-maximum of 3 cm^{-1}.

9.6 Py Normal Raman Spectrum

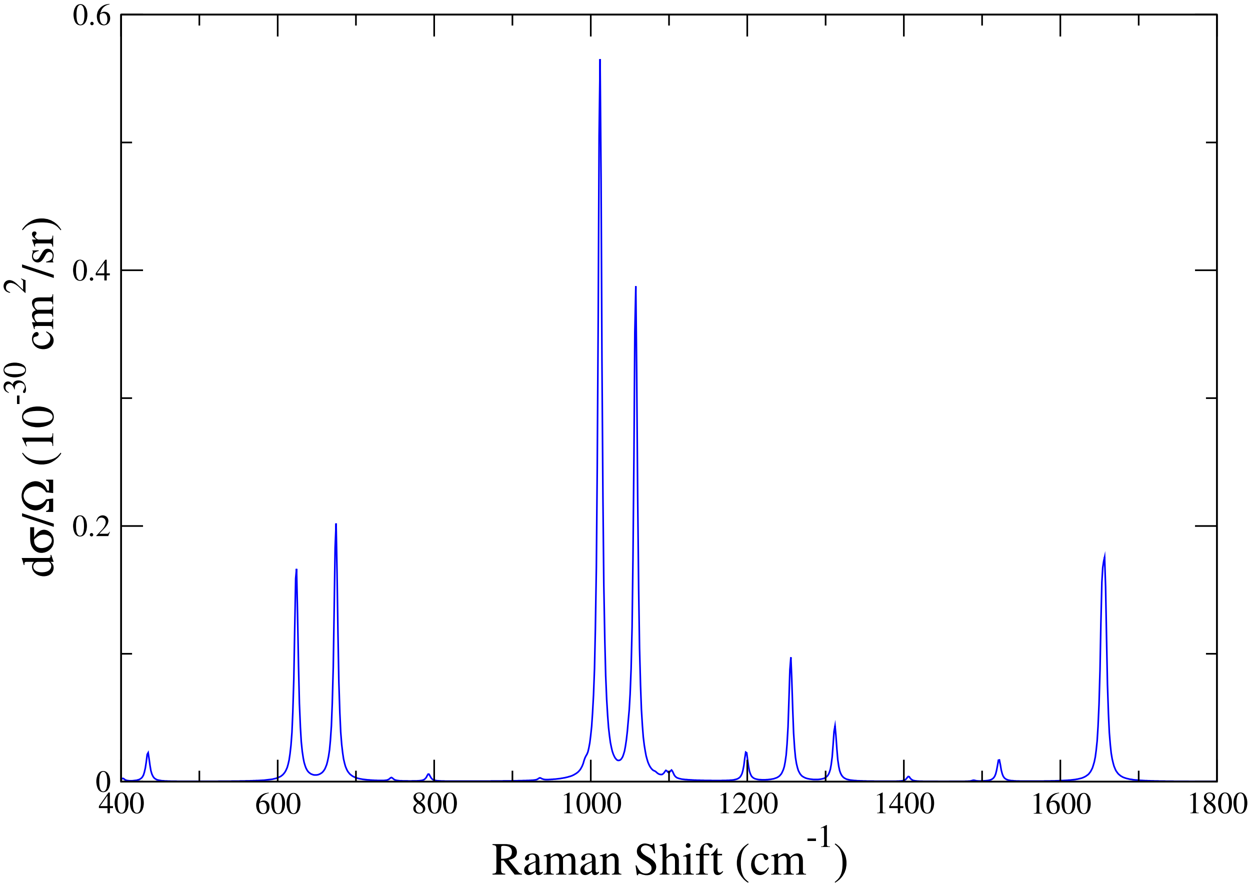

Figure S56: Normal Raman Spectra for Pyridine, LanL2DZ/CAM-B3LYP level of theory. Excitation Energy set at 514.5 nm. All stick transitions were convoluted with a Lorentzian of half-width at half-maximum of 3 cm^{-1}.

9.7 Results with $\gamma = 0.1$ eV

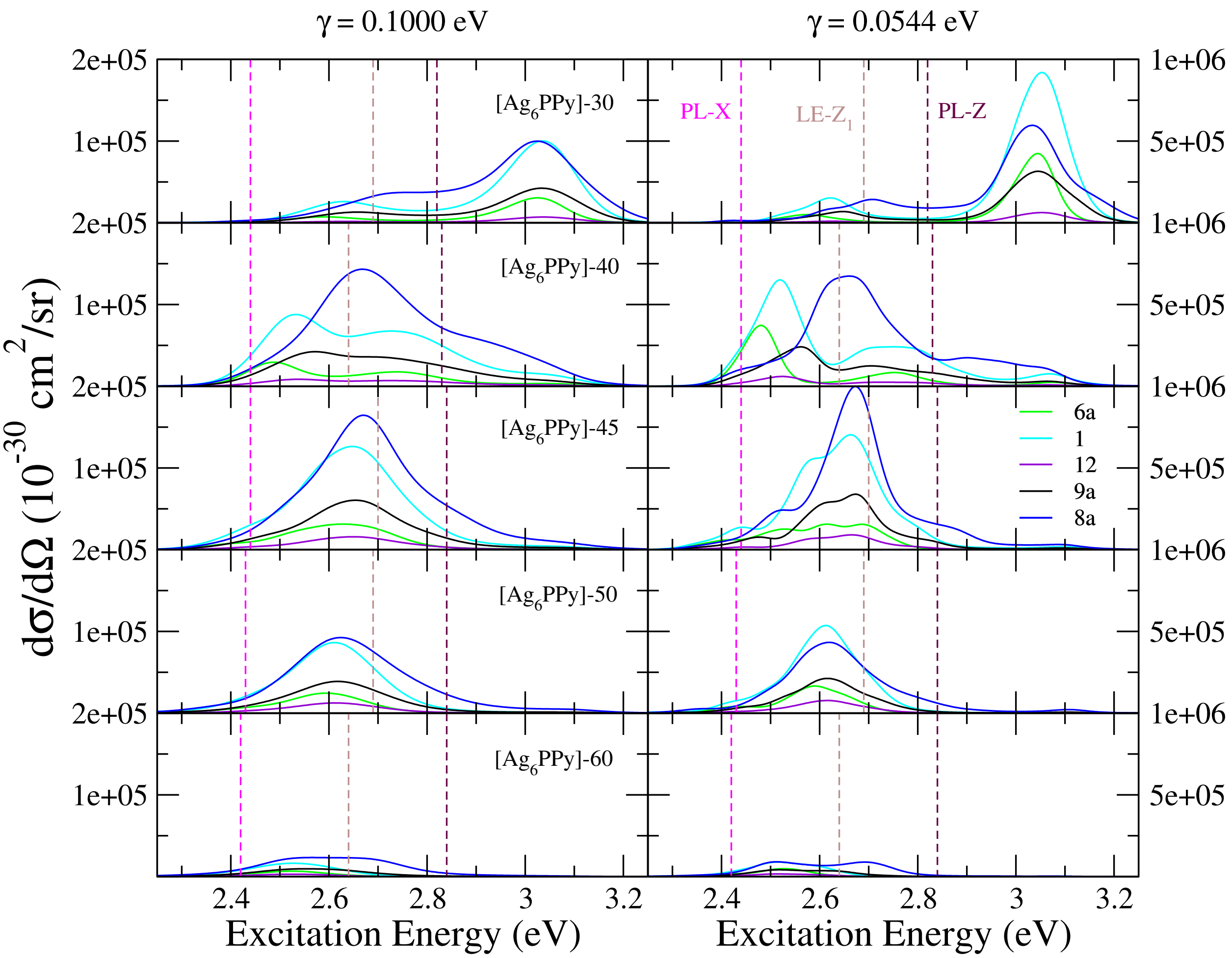

Figure S57: Comparison of Excitation Profiles for the most relevant Py normal modes with $\gamma = 0.1$ eV and $\gamma = 0.0544$ eV for $[Ag_6PPy]+\vec{E}$ systems.

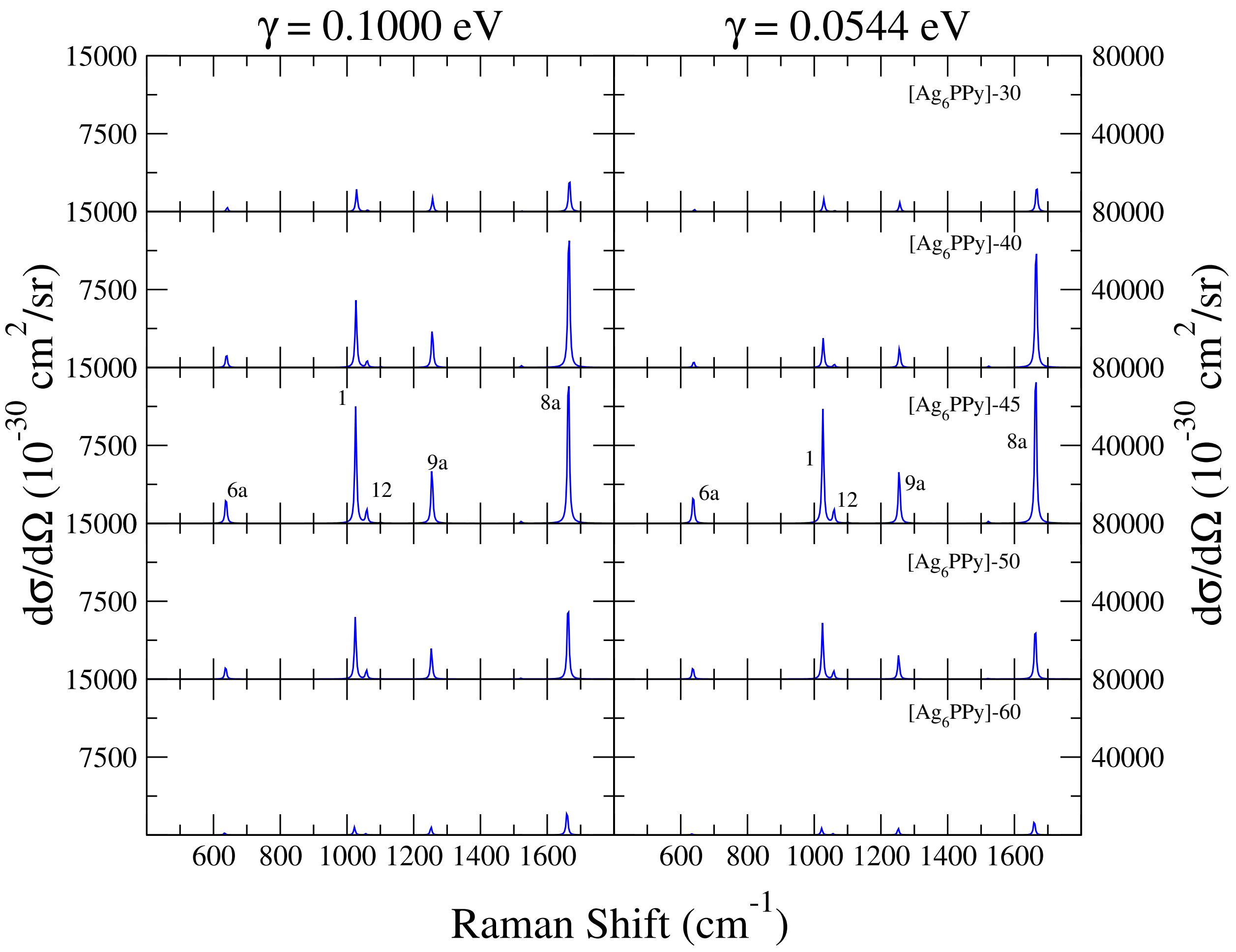

Figure S58: Comparison of Resonance Raman Spectra at excitation energy of the LE-Z_1 state with γ = 0.1 eV and γ = 0.0544 eV for $[Ag_6PPy]+\vec{E}$ systems. All stick transitions for the spectra were convoluted with a Lorentzian of HWHM = 3 cm^{-1}

9.8 Comparison with adiabatic methods

Table S8: Definition of Adiabatic state A-CT0-Z in terms of the combination of the Diabatic states CT0-Z, PL-Z, LE-Z_1 and LE-Z_2 for [Ag_6PPy] system, energy (in eV) and the components of the adiabatic electric transition dipole moment with respect to the ground state (g), μ_σ^{gk}, in atomic units. E expressed in 10^{-4} a.u.

		Weight						
System	Energy	CT0−Z	PL−Z	LE−Z_1	LE−Z_2	μ_x^{gk}	μ_y^{gk}	μ_z^{gk}
[Ag_6PPy]−30	3.22	0.88	0.01	0.08	0.03	0	0	−0.43
[Ag_6PPy]−40	2.85	0.85	0.10	0.05	0	0	0	1.93
[Ag_6PPy]−45	2.70	0.50	0.23	0	0.27	0	0	2.20
[Ag_6PPy]−50	2.35	0.61	0.13	0.23	0.03	0	0	-2.90
[Ag_6PPy]−60	2.05	0.91	0.02	0.06	0.01	0	0	−1.47

Table S9: Definition of Adiabatic state A-PL-Z in terms of the combination of the Diabatic states CT0-Z, PL-Z, LE-Z_1 and LE-Z_2 for [Ag_6PPy] system, energy (in eV) and the components of the adiabatic electric transition dipole moment with respect to the ground state (g), μ_σ^{gk}, in atomic units. E expressed in 10^{-4} a.u.

		Weight						
System	Energy	CT0−Z	PL−Z	LE−Z_1	LE−Z_2	μ_x^{gk}	μ_y^{gk}	μ_z^{gk}
[Ag_6PPy]−30	3.05	0.08	0.55	0.16	0.21	0	0	3.81
[Ag_6PPy]−40	3.08	0.03	0.52	0.19	0.25	0	0	3.75
[Ag_6PPy]−45	3.11	0.01	0.51	0.22	0.26	0	0	3.56
[Ag_6PPy]−50	3.11	0	0.52	0.19	0.28	0	0	3.67
[Ag_6PPy]−60	3.10	0	0.54	0.14	0.32	0	0	3.89

Table S10: Definition of Adiabatic state A-LE-Z_1 in terms of the combination of the Diabatic states CT0-Z, PL-Z, LE-Z_1 and LE-Z_2 for [Ag_6PPy] system, energy (in eV) and the components of the adiabatic electric transition dipole moment with respect to the ground state (g), μ_σ^{gk}, in atomic units. E expressed in 10^{-4} a.u.

		Weight						
System	Energy	CT0−Z	PL−Z	LE−Z_1	LE−Z_2	μ_x^{gk}	μ_y^{gk}	μ_z^{gk}
[Ag_6PPy]−30	2.50	0.04	0.40	0.47	0.10	0	0	3.82
[Ag_6PPy]−40	2.43	0.11	0.30	0.52	0.08	0	0	4.03
[Ag_6PPy]−45	2.44	0.29	0.26	0.38	0.06	0	0	3.88
[Ag_6PPy]−50	2.60	0.38	0.30	0.25	0.07	0	0	3.57
[Ag_6PPy]−60	2.50	0.08	0.34	0.50	0.08	0	0	4.17

Table S11: Definition of Adiabatic state A-LE-Z_2 in terms of the combination of the Diabatic states CT0-Z, PL-Z, LE-Z_1 and LE-Z_2 for [Ag_6PPy] system, energy (in eV) and the components of the adiabatic electric transition dipole moment with respect to the ground state (g), μ_σ^{gk}, in atomic units. E expressed in 10^{-4} a.u.

		Weight						
System	Energy	CT0−Z	PL−Z	LE−Z_1	LE−Z_2	μ_x^{gk}	μ_y^{gk}	μ_z^{gk}
[Ag_6PPy]−30	2.67	0	0.04	0.29	0.67	0	0	0.26
[Ag_6PPy]−40	2.70	0.01	0.08	0.24	0.67	0	0	-0.29
[Ag_6PPy]−45	2.73	0.20	0	0.40	0.40	0	0	1.38
[Ag_6PPy]−50	2.74	0	0.05	0.32	0.62	0	0	0.13
[Ag_6PPy]−60	2.77	0	0.10	0.31	0.60	0	0	-0.38

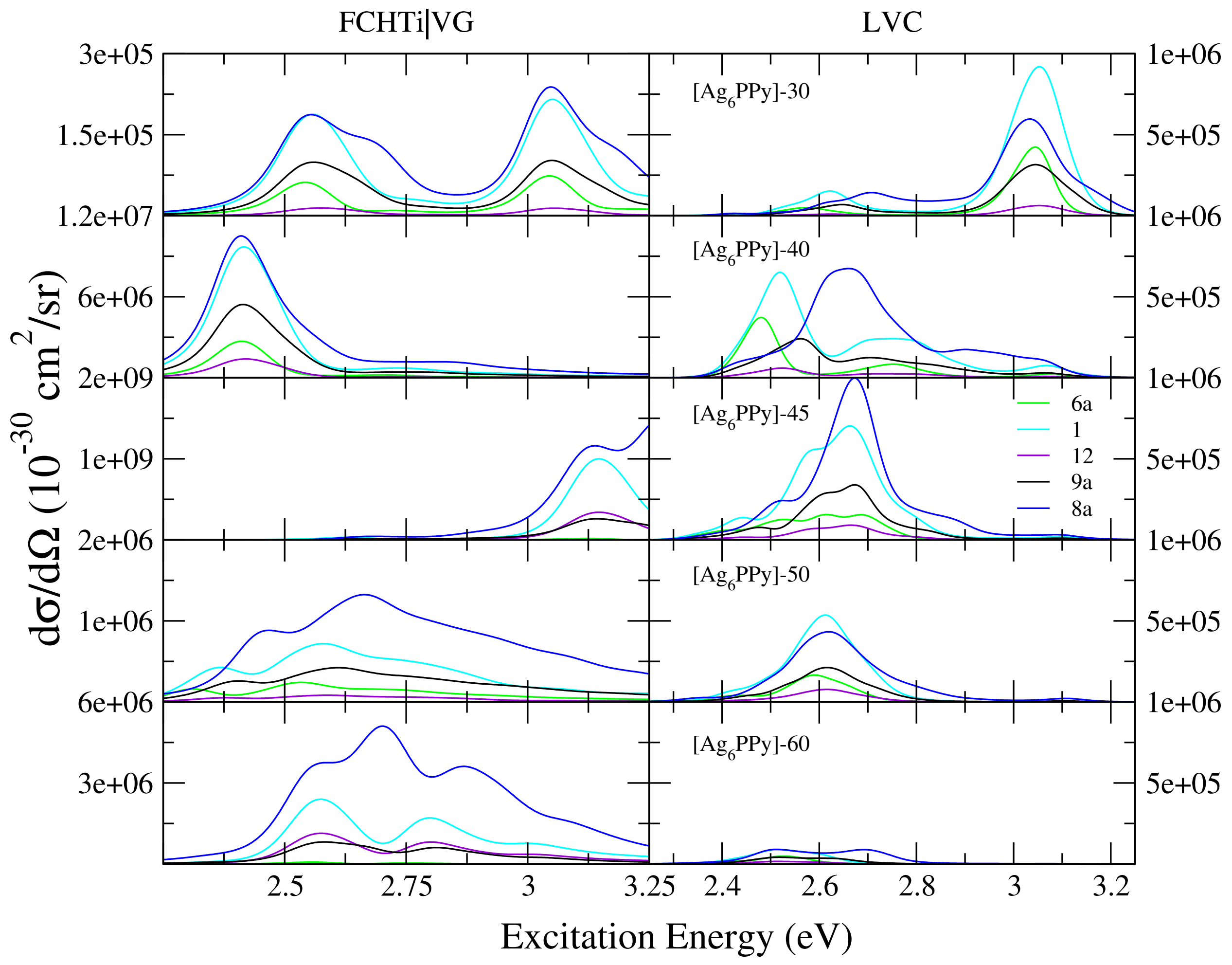

Figure S59: Comparison of FCHTi|VG and LVC excitation profiles for [Ag_6PPy] system. 3 CT adiabatic states were included for FCHTi|VG.

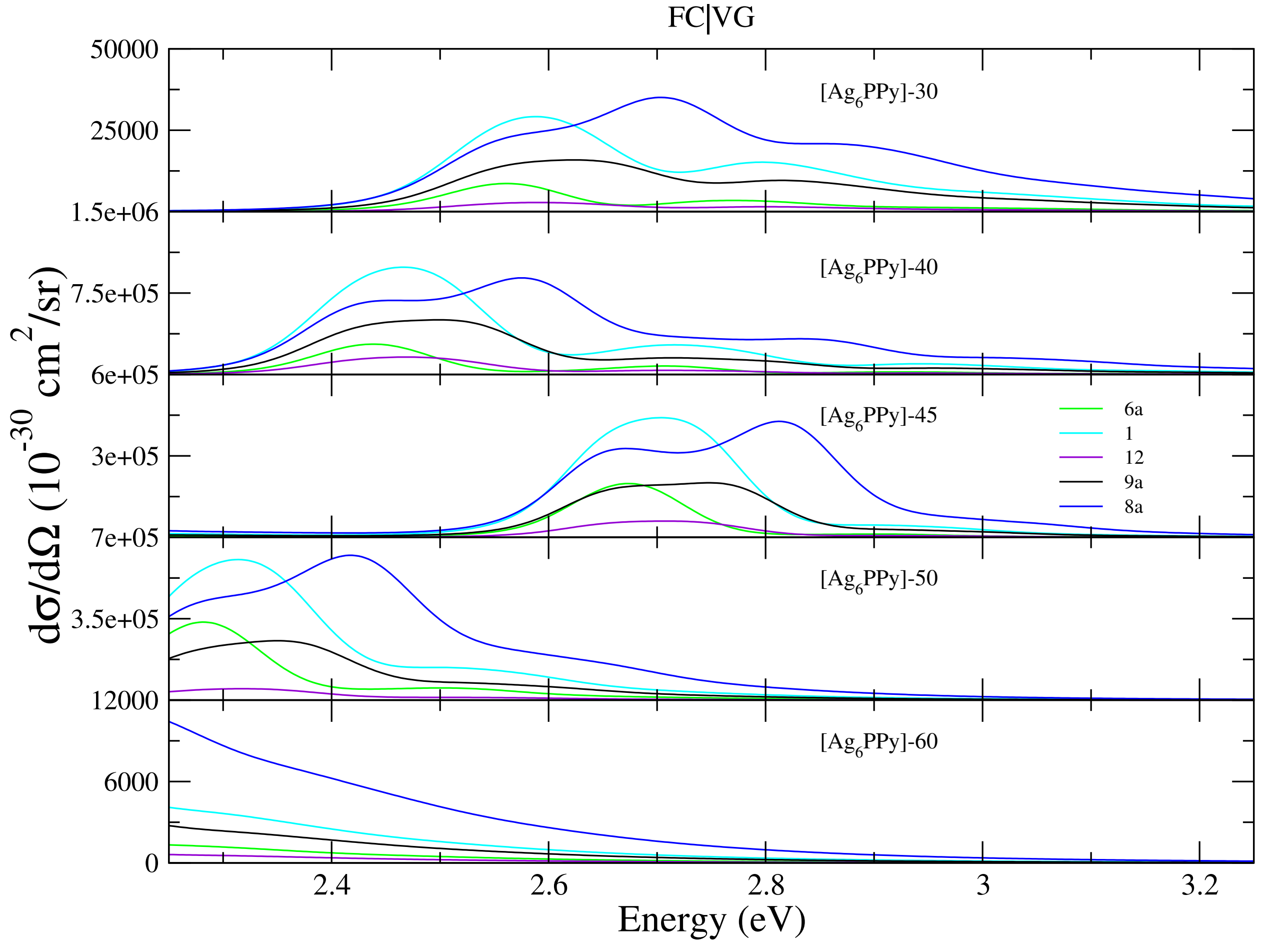

Figure S60: FC|VG excitation profiles for [Ag_6PPy] system. 3 CT adiabatic states were included for FC|VG.

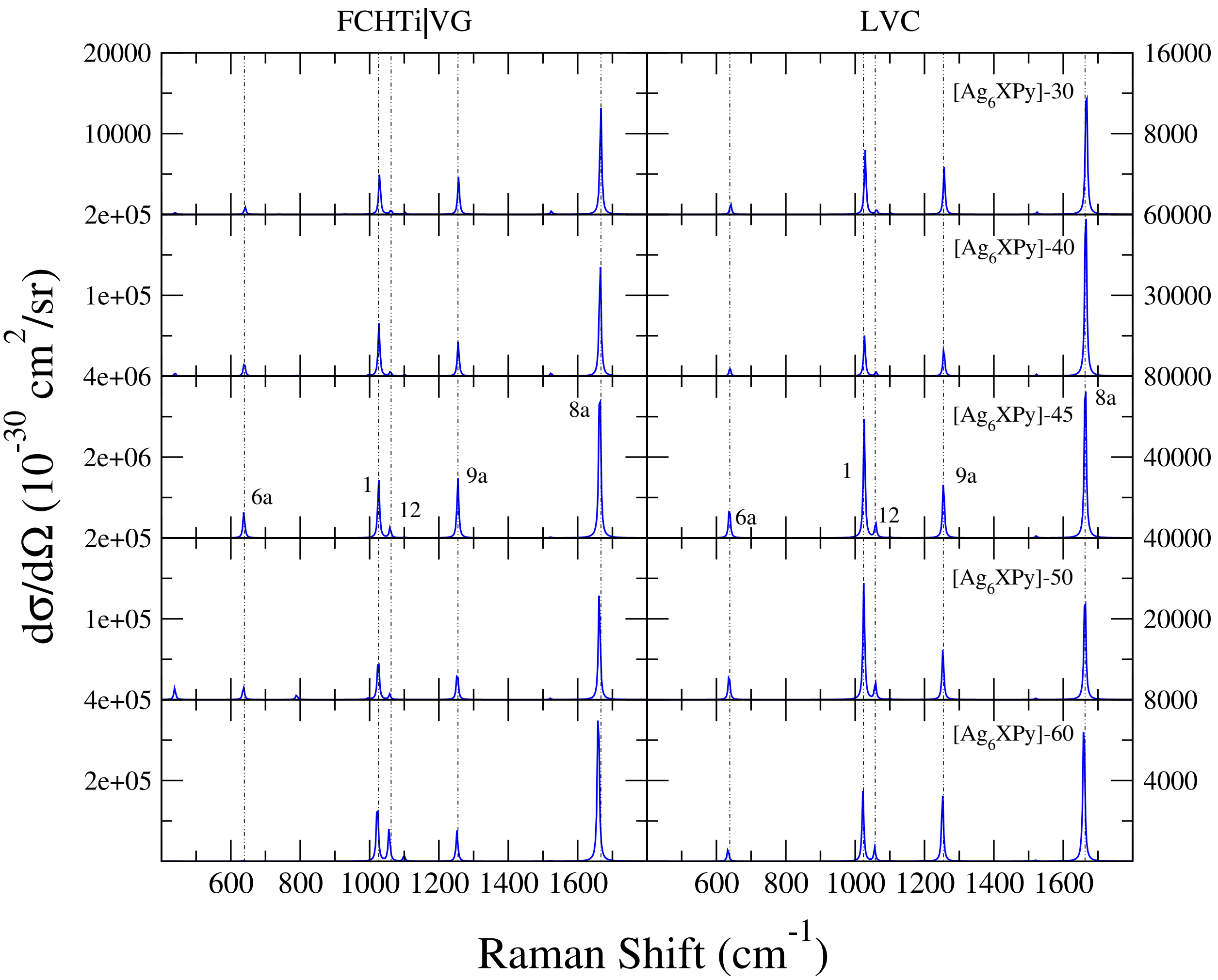

Figure S61: Comparison of FCHTi|VG and LVC spectra for [Ag_6PPy] system. 3 CT adiabatic states were included for FCHTi|VG. All stick transitions were convoluted with a Lorentzian of hal-width at half-maximum of 3 cm^{-1}

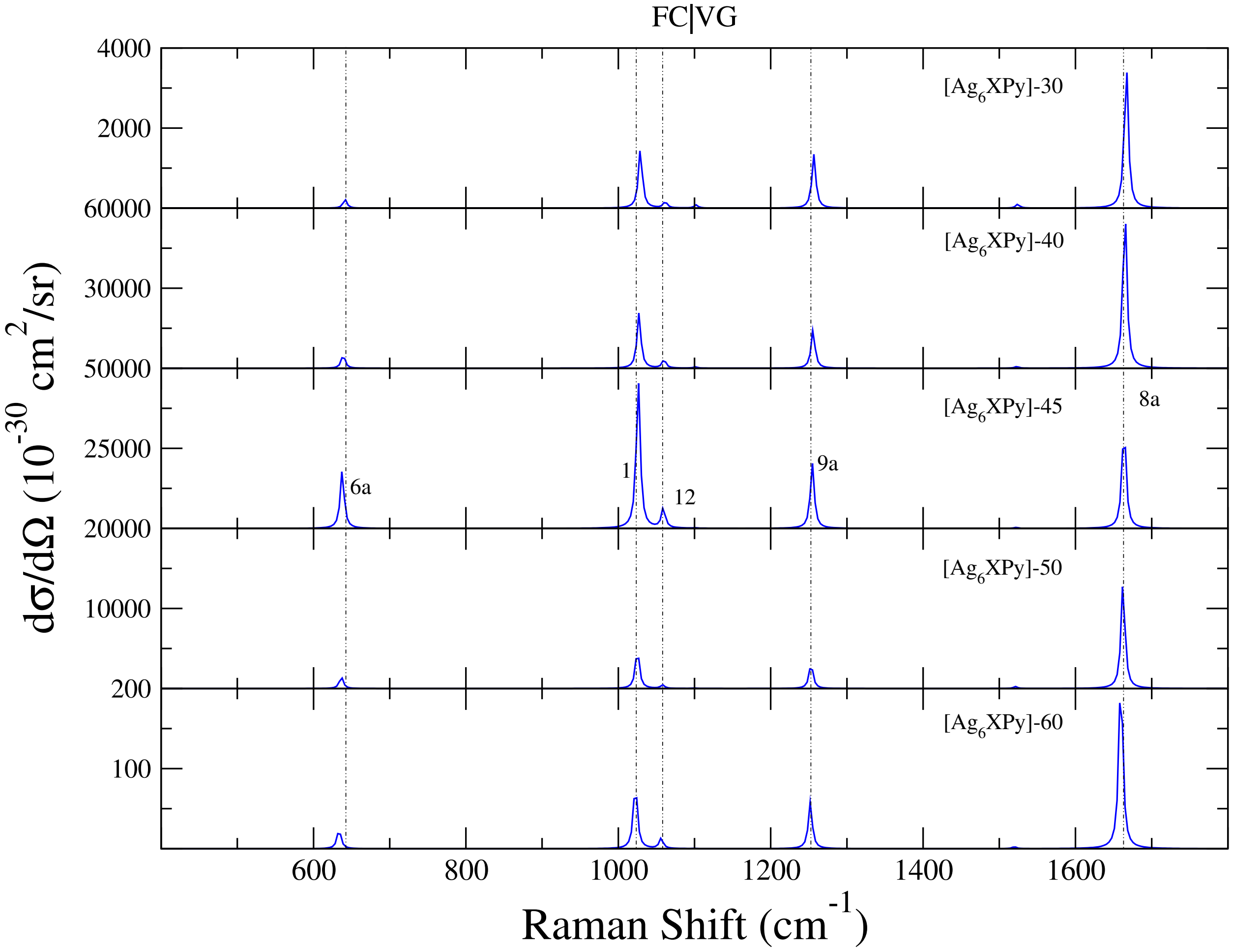

Figure S62: FC|VG spectra for [Ag_6PPy] system. 3 CT adiabatic states were included for FC|VG. All stick transitions were convoluted with a Lorentzian of hal-width at half-maximum of 3 cm^{-1}

References

(S1) Yaghoubi Jouybari, M.; Liu, Y.; Improta, R.; Santoro, F. The ultrafast dynamics of the two lowest bright excited states of cytosine and 1-methyl-cytosine: A quantum dynamical study. *J. Chem. Theory Comput.* **2020**, *16*, 5792–5808.

(S2) Green, J. A.; Yaghoubi Jouybari, M.; Asha, H.; Santoro, F.; Improta, R. Fragment diabatization linear vibronic coupling Model for quantum dynamics of multichromophoric systems: population of the charge-transfer state in the photoexcited guanine–cytosine pair. *J. Chem. Theory Comput.* **2021**, *17*, 4660–4674.

(S3) Xu, Q.; Aranda, D.; Martha, Y. J.; Liu, Y.; Wang, M.; Cerezo, J.; Improta, R.; Santoro, F. Nonadiabatic vibrational resonance Raman spectra from quantum dynamics propagations with LVC models. Application to thymine. *J. Phys. Chem. A* **2022**, *126*, 7468–7479.

(S4) Aranda, D.; Valdivia, S.; Soto, J.; López-Tocón, I.; Avila, F. J.; Otero, J. C. Theoretical Approaches for modeling the effect of the electrode potential in the SERS vibrational wavenumbers of pyridine adsorbed on a charged silver surface. *Front. Chem.* **2019**, *7*, 423.
